# Sediment Cores from White Pond, South Carolina, contain a Platinum Anomaly, Pyrogenic Carbon Peak, and Coprophilous Spore Decline at 12.8 ka

**DOI:** 10.1038/s41598-019-51552-8

**Published:** 2019-10-22

**Authors:** Christopher R. Moore, Mark J. Brooks, Albert C. Goodyear, Terry A. Ferguson, Angelina G. Perrotti, Siddhartha Mitra, Ashlyn M. Listecki, Bailey C. King, David J. Mallinson, Chad S. Lane, Joshua D. Kapp, Allen West, David L. Carlson, Wendy S. Wolbach, Theodore R. Them, M. Scott Harris, Sean Pyne-O’Donnell

**Affiliations:** 1 Savannah River Archaeological Research Program, South Carolina Institute of Archaeology and Anthropology, University of South Carolina, P.O. Box 400, New Ellenton, SC 29809 USA; 2 South Carolina Institute of Archaeology and Anthropology, Columbia, SC 29208 USA; 3 Department of Environmental Studies, Wofford College, 429N Church Street, Spartanburg, SC 29303-3663 USA; 4 University of Wisconsin, Geography Department, 550N Park Street, Madison, WI 53707-1404 USA; 5 Department of Geological Sciences, East Carolina University, Greenville, NC 27858-4353 USA; 6 Department of Biology, East Carolina University, Greenville, NC 27858-4353 USA; 7 Department of Chemistry, East Carolina University, Greenville, NC 27858-4353 USA; 8 Department of Earth and Ocean Sciences, University of North Carolina Wilmington, Wilmington, NC 28411 USA; 9 Department of Ecology and Evolutionary Biology, University of California, Santa Cruz, CA 95064 USA; 10 Comet Research Group, Prescott, AZ USA; 11 Department of Anthropology, Texas A&M University, College Station, TX 77843-4352 USA; 12 Department of Chemistry and Biochemistry, DePaul University, Chicago, IL 60614 USA; 13 Department of Geology and Environmental Sciences, College of Charleston, Charleston, SC 29424 USA; 14 Earth Observatory of Singapore and Asian School of the Environment, Nanyang Technological University, Singapore, Singapore

**Keywords:** Limnology, Limnology

## Abstract

A widespread platinum (Pt) anomaly was recently documented in Greenland ice and 11 North American sedimentary sequences at the onset of the Younger Dryas (YD) event (~12,800 cal yr BP), consistent with the YD Impact Hypothesis. We report high-resolution analyses of a 1-meter section of a lake core from White Pond, South Carolina, USA. After developing a Bayesian age-depth model that brackets the late Pleistocene through early Holocene, we analyzed and quantified the following: (1) Pt and palladium (Pd) abundance, (2) geochemistry of 58 elements, (3) coprophilous spores, (4) sedimentary organic matter (OC and sedaDNA), (5) stable isotopes of C (δ^13^C) and N (δ^15^N), (6) soot, (7) aciniform carbon, (8) cryptotephra, (9) mercury (Hg), and (10) magnetic susceptibility. We identified large Pt and Pt/Pd anomalies within a 2-cm section dated to the YD onset (12,785 ± 58 cal yr BP). These anomalies precede a decline in coprophilous spores and correlate with an abrupt peak in soot and C/OC ratios, indicative of large-scale regional biomass burning. We also observed a relatively large excursion in δ^15^N values, indicating rapid climatic and environmental/hydrological changes at the YD onset. Our results are consistent with the YD Impact Hypothesis and impact-related environmental and ecological changes.

## Introduction

Study of late Quaternary climates and their effect on vegetation dynamics of southeastern North America has been hampered by the limited number of radiocarbon-dated pollen sequences extending from the Last Glacial Maximum (LGM) through the Holocene transition. Early paleoenvironmental reconstructions by Watts^1^ established White Pond in South Carolina as one of the oldest and most complete paleoenvironmental records in southeastern North America, with a basal core date of at least 22,000 calendar years BP. Watts^1^ report on the Pleistocene-Holocene transition at White Pond has served as a benchmark study of paleoclimates and paleoecology for the Atlantic Coastal Plain. Paleoecologists, as well as archaeologists, have a shared interest in the Pleistocene-Holocene transition, particularly the sudden onset of the Younger Dryas (YD) stadial referred to as the Younger Dryas Boundary (YDB). For example, a recent study by Krause *et al*.^2^ reports analysis of a nearly 6-m-long core from White Pond for paleoenvironmental reconstruction, including fossil pollen, macroscopic charcoal, *Sporormiella*, and a paleotemperature reconstruction based on branched glycerol dialkyl tetraethers (brGDGTs).

Interest in lacustrine paleoenvironmental records such as White Pond, has recently increased due to evidence of an extraterrestrial (ET) impact that is proposed to have caused the YD climatic anomaly^3^. In 2015, we worked with the USGS climate study team to obtain a core from White Pond suitable for precise dating and analysis of the lower YDB. Following identification of the YDB through AMS dating (Supplementary Fig. 7), we obtained additional cores in 2016 to test for impact and other environmental proxies. These analyses included Pt, with elevated levels proposed to be associated with an ET impact^4,5^, and analyses to test for coprophilous spores, sedimentary organic material including DNA, pyrogenic carbon, C and N stable isotopes, magnetic susceptibility of bulk sediments, cryptotephra, and comprehensive elemental geochemistry. Pt and Pt/Pd anomalies have been documented as a widespread chronostratigraphic marker associated with the YDB onset within terrestrial sedimentary sequences^4^, and so, White Pond offers the potential for very high-resolution sampling and dating of this boundary in a lacustrine setting.

Petaev *et al*.^5^ report a large Pt anomaly within annual layers of the Greenland Ice Sheet Project (GISP2) ice core that date to the onset of the YD. They conclude that the likely source of the Pt enrichment was from multiple atmospheric injections of platinum-rich dust following an extraterrestrial impact and the subsequent 21-year-long deposition of platinum within interannual layers of ice. Following this discovery, an independent contribution by Moore *et al*.^4^ reported a widespread Pt anomaly at 11 geographically-separated and diverse terrestrial sedimentary sequences across North America. These results are consistent with those previously reported by Petaev *et al*.^5^ and indicate the Pt anomaly is a robust chronostratigraphic marker or datum for the chronostratigraphic position of the YD onset in terrestrial sedimentary sequences. These results are also consistent with the Younger Dryas Impact Hypothesis (YDIH), which proposed that an extraterrestrial source contributed Pt along with other reported impact proxies (e.g., microspherules, nanodiamonds, soot, etc.) over large portions of the Northern Hemisphere and likely over the entire globe (e.g., Firestone *et al*.^3^; Kennett *et al*.^6^; Melott *et al*.^7^; Bunch *et al*.^8^; Israde-Alcántara *et al*.^9^; Wittke *et al*.^10^; Andronikov *et al*.^11^; Kinzie *et al*.^12^; Andronikov *et al*.^13^; Andronikov and Andronikova^14–16^). Recent studies by Wolbach *et al*.^17,18^ have provided additional evidence in the form of multiple proxies for large-scale biomass-burning and a brief impact winter triggered at ~12,800 years ago. Those studies were based on analyses of large numbers of terrestrial, lacustrine, marine, and ice core records with peaks in biomass-burning proxies such as charcoal, pyrogenic carbon (soot and aciniform carbon, as described in Wolbach *et al*.^18^), and combustion aerosols, such as ammonium. These previous studies are significant in that they point to rapid global climate change and ecological disruptions/reorganizations that occurred at the beginning of the YD.

Most recently, Kjaer *et al*.^19^ report the discovery of a massive (31 km) impact crater of possible YD-age under the Hiawatha Glacier in northwestern Greenland. Although yet to be dated, the impact crater exhibits evidence of a geologically young age, including the absence of pre-YD ice within the crater, evidence of residual heat, and a minimally eroded crater rim. Furthermore, Kjaer *et al*.^19^ report platinum group elements (PGE) anomalies similar to those reported from the GISP2 ice core by Petaev *et al*.^5^.

We acknowledge that numerous papers have been published that criticize or take issue with one or more purported impact proxies suggested to be evidence of an extraterrestrial impact at the YD onset as originally set forth by Firestone *et al*.^3^. A review of the major themes of these papers was recently included in Pino *et al*.^20^. For a short discussion of these critical papers see Supplementary Information; “Critical Overview of YDIH”.

Here we report the results of analyses of cores collected from White Pond in South Carolina. We investigate whether the White Pond record contains any of the previously reported impact proxies that are predicted by the YDIH. These include analyses of coprophilous spores, sedimentary organic material including ancient DNA, stable isotope composition, pyrogenic carbon (soot and aciniform carbon), cryptotephra, mercury (Hg), magnetic susceptibility, and elemental geochemistry, including quantification of Pt and Pd. These cores are particularly well-suited for this objective because they contain sediments that bracket the Pleistocene-Holocene transition and include a well-dated (~10 cm-thick) YD onset sequence (modeled age: 12,835–12,735 cal yr BP at 95% confidence interval)^21^.

## Study Site

**Figure 1:**
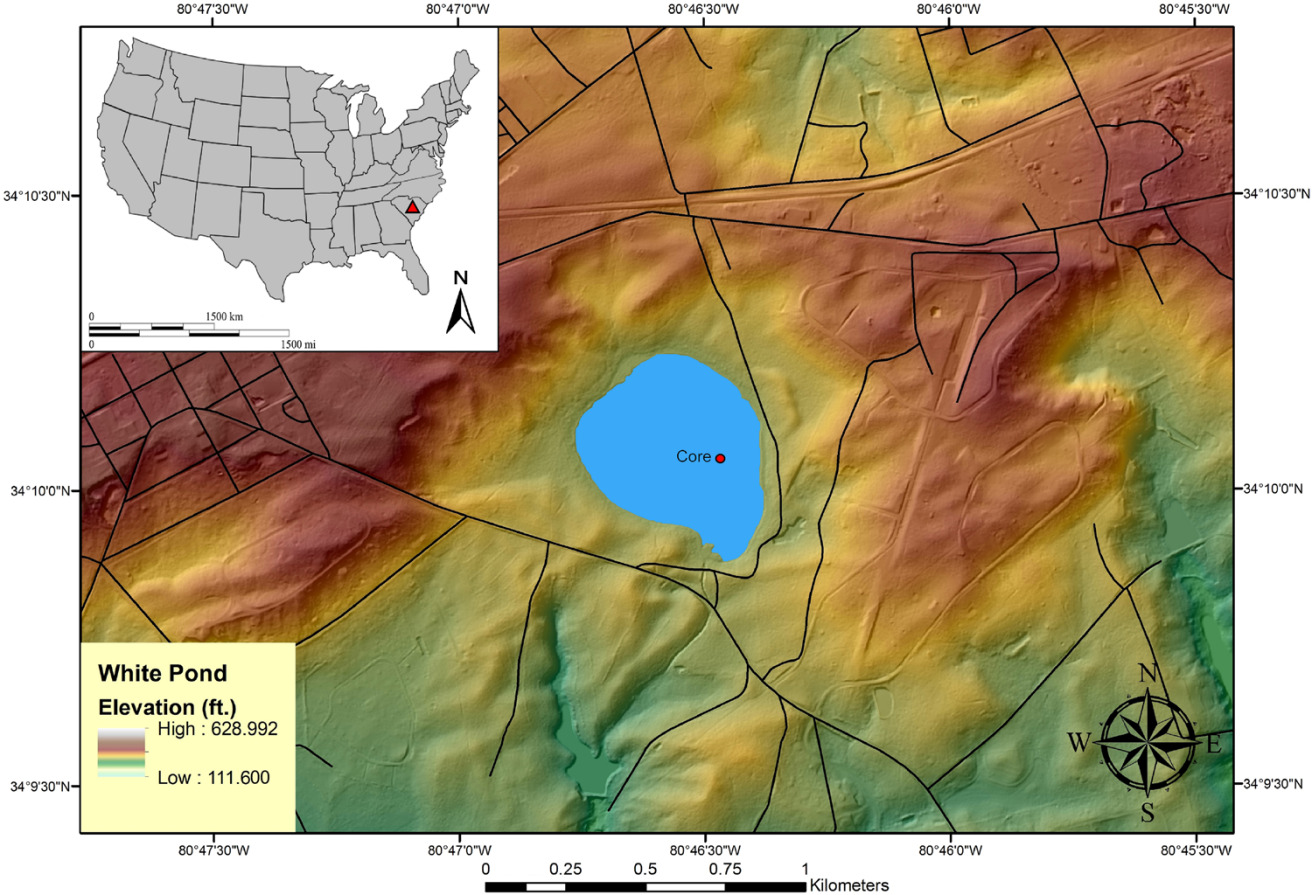
LiDAR image of White Pond near Elgin, South Carolina, showing location of vibracores collected in 2015 and 2016.

White Pond, a natural lake situated along the western edge of the Upper Coastal Plain in central South Carolina (Fig. 1), covers nearly 26 hectares and generally exhibits a shallow water depth of less than 2 m in the deepest portions. Eolian and fluvial sand deposits surround most of the lake and are underlain by heavily weathered Cretaceous and Tertiary clayey sand deposits. Within the lake itself, peat, organic-rich mud, and silt deposits nearly 6-m thick have accumulated since the Last Glacial Maximum (LGM) and possibly earlier based on recent work by Krause *et al*.^2^ who report a basal core date of ca. 31–32 ka at 5.5 m. The lake appears to be within a streamhead depression formed by scour and the downstream blockage of the drainage by large Pleistocene sand dunes on the south end of the lake. Shaping and rounding of the lake have occurred through lacustrine processes common to those involved in the formation of Carolina bays (i.e., directional winds on shallow ponded water; see Moore *et al*.^22^ for an explanation of Carolina bay formation and evolution).

## Results

### Core lithology

**Figure 2:**
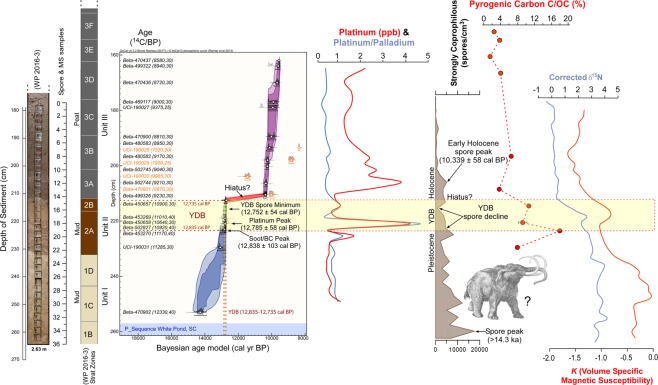
Composite figure for White Pond showing core lithology for the lowermost portion of core 2016-3, depths for stratigraphic units, a Bayesian age/depth model based on 22 AMS dates (Supplementary Tables 1, 2), platinum (Pt) abundance (error = +/−0.1 ppb), the ratio of platinum to palladium (Pt/Pd), strongly coprophilous spore concentration data (spores/cm^3^), pyrogenic carbon [C/OC (%)], bulk sediment δ^15^N, and volume specific magnetic susceptibility. Bayesian modeled age range for the YD onset (12,835–12,735 cal yr BP at 95% Confidence Interval) based on Kennett *et al*.^21^ is shown as a light-yellow zone within stratigraphic Unit II. Spore peaks with modeled ages are shown for Strongly Coprophilous spores along with the YDB spore decline and hiatus. Spore sample 15 (top of Unit IIb and bottom of Unit IIIa) overlaps with the modeled core hiatus between 211–212 cm in Core 2016-3. Increasing spore counts (spores/cm^3^) for this sample at the top of the modeled YDB interval are likely due to inclusion of Early Holocene spores present in the core during or post-hiatus. Data sets used in this figure come from duplicate cores that are correlated using lithostratigraphic Unit II as a common core datum (see Supplementary Figs 8, 9).

White Pond core lithology can be roughly divided into three units (Fig. 2 and Supplementary Figs 7, 8). Depositional units delineated by texture, color, sedimentary structures, and clearly defined boundaries may be discontinuities signaling a change in depositional environment or may be true erosional unconformities. Three distinctive units (I–III) and associated subunits are delineated within the studied portion of the cores and are referenced here to Core 2016-3. The stratigraphy depicted represents only slightly more than the upper half of the basin-fill sequence described by Krause *et al*.^2^. Unit I, the lowermost (≥232 cm; ≥13.2 ka), contains deformed and laminated muds (clay and silt) interspersed with organics, occasional seeds, charcoal, and plant macrofossils. Cores 2016-2 and 2016-3 consist mainly of interbedded gray clay and silt layers at the bottom, gradually replaced by organics and soft sediment deformation toward the top of the unit. Unit II (232 to 213 cm; 13.2 to 12.7 ka) is an organic matter-rich, muddy transition unit with continued evidence of some soft sediment deformation in Unit IIA of Cores 2016-2 and 2016-3. Unit IIB in several cores is visually distinct due to a slight color change indicative of oxidation and possible subaerial exposure. Unit III, the uppermost (≤213 cm; ≤12.7 ka), consists of a silty, very fine, degraded, peat, with the peat becoming coarser with thin (~1.0-1.5 cm), intercalated charcoal lenses towards the top of the core. Magnetic susceptibility measurements made on a section of Core 2016-3 provide a proxy for composition, texture, and depositional history (see Fig. 2 and Supplementary Information).

### Bayesian age/depth model

We conducted Bayesian analysis (Ramsey^23,24^) using OxCal (v.4.3.2) to construct an age/depth model with 22 AMS dates from a 1-meter section of vibracore from White Pond. This corresponds to ~1.63 to 2.63 m below the sediment/water interface referenced to Core 3 (Fig. 2 and Supplementary Tables 1, 2). For construction of the age/depth model, 19 AMS dates are from core 2016-3, 2 AMS dates are from core 2016-2, and 1 AMS date is from core 2016-1 (see Supplementary Fig. 8). Multiple radiocarbon dates and Bayesian modeling confirm the presence of a ~10 cm section of core that was deposited at, or during, the YD onset (ca.12,835 to 12,735 cal yr BP). Sediments dating to the YD onset (YDB) are bracketed by underlying older Pleistocene muds and overlying muddy peats that span the early to middle Holocene. The boundary from organic matter-rich mud to Holocene-age muddy peat is abrupt and may represent an unconformity/hiatus or an interval of very slow sedimentation. Bayesian modeling of AMS dates (Fig. 2) show sediments that date to the YD onset transitioning directly to early Holocene muddy peat with little to no later YD sediments present. In this transition (occurring between 211 to 212 cm in core 2016-3), the Bayesian age/depth model indicates a ca. 2,400-year hiatus or unconformity. A similar situation was observed at terrestrial archaeological sites in the Carolinas along the sandy Coastal Plain, in which Paleoindian and Early Archaic artifacts spanning this temporal interval are stratigraphically conflated^25^. This stratigraphic pattern indicates that the hiatus may signal a regional climate signature of an interval of very slow deposition or erosion/non-deposition beginning during the Younger Dryas and continuing to the early Holocene.

### Platinum geochemistry

A large Pt and Pt/Pd anomaly exists within a single 2-centimeter section of core dated to the YD onset with a Bayesian modeled age of 12,785 ± 58 cal yr BP (Fig. 2, Supplementary Fig. 10, and Supplementary Table 3). The Pt and Pt/Pd anomaly are located within the middle portion of Unit II (Unit IIa), dated to the YD onset. The largest Pt anomaly is in Unit II, associated with organic-stained dark gray clay and silt. Directly above this unit, sediments transition abruptly to a muddy, sapric peat (Unit III) that dates to the early Holocene. A second smaller Pt peak is present in the peat above this transition at the base of Unit III; however, overall background levels for Pt are higher within Holocene peat than the underlying Pleistocene mud and only Unit II has both a large Pt anomaly and a large Pt/Pd anomaly.

### Coprophilous fungi and sedimentary ancient DNA

Coprophilous fungi across the YDB are a potential indicator of megaherbivore presence (e.g., wooly mammoths and mastodons) and relative abundance just before, during, and immediately following YD climate change^26–28^ (Fig. 2 and Supplementary Information). Results from White Pond indicate a decline in the abundance of multiple species of coprophilous fungi within sediments dated to the YD onset (Supplementary Fig. 3). However, estimates for the timing of the final megafaunal extinction are uncertain due to the possible unconformity within the core sediments immediately post-dating the YD onset and lasting into the early Holocene (see Discussion below and Supplementary Fig. 2). An attempt to confirm the presence and/or absence of megafauna using core samples via extraction and characterization of sedimentary ancient DNA from sediment plugs was unsuccessful (see Supplementary Information). Despite our use of targeted enrichment for mammalian DNA, the material was too poorly preserved in this warm and wet environment^29^ for this approach to be successful. There is no independent body of evidence documenting the presence of now-extinct megafauna in the area at the YD onset. Although the evidence may yet be found, recent blood antisera residue analyses of Paleoindian stone points from the Carolinas and Georgia, representing the time interval immediately prior to the YDB, exhibited no evidence of predation on now-extinct megafauna^30^.

### Organic and pyrogenic carbon

Although pyrogenic carbon includes both aciniform and non-aciniform morphologies, we will henceforth refer to these non-aciniform morphologies of pyrogenic origin as “soot”. We analyzed sediments for bulk sedimentary organic matter (OC) and soot carbon concentrations (soot C) and their stable isotopic signatures (δ^13^C_OC_, δ^13^C_soot C_) in core 2016-1 (see Supplemental Fig. 12). Stable carbon isotopic signatures can provide information on the sources of sedimentary organic matter (SOM) and soot C deposited at this site. From the bottom of core 2016-1 proceeding upwards, SOM abundance remains <15% of the dry weight of sediment. δ^13^C_SOM_ systematically decreases from −23.5 to −27.5‰ proceeding up-core until about halfway up the core at a depth of 250 to 213 cm (Bayesian modeled age = ca. 14.2 to 12.7 cal ka). Upward from this point, there is an abrupt increase in the concentration of OC while C/N ratios increase and δ^13^C_OC_ reach some of their lowest values indicating an increase in the contribution of terrestrially-derived organic matter to the White Pond sedimentary organic matter pool.

In core 2016-1, a soot C/OC (%) maximum is present in the same lithologic unit (Unit II) as the Pt and Pt/Pd anomaly in core 2016-3, with a Bayesian modeled age of 12,838 ± 103 cal yr BP (Fig. 2 and Supplementary Fig. 11). In fact, the soot anomaly is the largest in the entire ca. 15,000-year core record. Several other large soot peaks also occur in Unit II that are well above background and correlate with the Pt and Pt/Pd anomalies and pre-Holocene spore minimum. A separate analysis of core samples from White Pond found no evidence for aciniform carbon, a subset of pyrogenic carbon.

### Bulk sedimentary stable carbon and nitrogen isotopes

The carbon and nitrogen stable isotope composition of bulk SOM was analyzed for a subset of samples spanning the YDB in core 2016-3 (Supplementary Fig. 13). We observed a large (~3‰) decrease in δ^15^N values and intermediate δ^13^C values during the YD (Unit II) relative to the remainder of the record indicating rapid climatic and environmental/hydrological changes. In particular, δ^15^N values decrease significantly starting in the middle portion of Unit II immediately above the Pt and Pt/Pd anomaly and soot maximum (Fig. 2).

### Cryptotephra

Commonly called ash or tephra, this is the glassy, explosively-erupted pyroclastic products of a volcanic eruption. High concentrations of Pt have sometimes been reported in tephra and ash from some, but not all volcanic eruptions, including none in tephra from Cascade volcanoes, which represent the closest active volcanoes upwind of White Pond^4^. Investigating whether there was any such association between cryptotephra and Pt at White Pond, we found a few randomly distributed tephra shards, presumably representing typical background atmospheric deposition (see Supplementary Information, “Cryptotephra”), but we found no evidence of volcanic cryptotephra associated with either the YDB layer or with the Pt and Pt/Pd anomalies. This negative finding indicates that the Pt anomaly at White Pond did not originate from terrestrial magmatic sources (see discussion of Potential Sources of YDB Platinum in Supplementary Information from Moore *et al*.^4^).

### Mercury

Mercury (Hg) concentrations of White Pond sediments were analyzed to determine if any perturbation to the Hg cycle occurred across the YDB layer. It is possible to generate sedimentary Hg anomalies through a multitude of natural biotic and abiotic processes, including increased organic matter deposition, volcanism, biomass burning, soil and rock weathering and erosion (detrital input), and local redox changes (see Them *et al*.^31^ and Grasby *et al*.^32^). Mercury contents increase by ~two-fold during the YDB interval (relative to Hg contents directly below this interval) at White Pond but represent the average Hg value across all the intervals analyzed (Supplementary Fig. 17).When normalized to loss-on-ignition (Hg/LOI) and total organic carbon (Hg/TOC), this change in Hg contents is muted (see Supplementary Information and Supplementary Figs 18, 19). Mercury (both Hg and Hg/LOI) also displays some relationship with fine grain sizes, suggesting a potential control of grain size on sedimentary Hg contents (see Supplementary Fig. 20 and Supplementary Table 12). The only anomalous Hg, Hg/LOI, and Hg/TOC values occur well below the YDB layer, and therefore cannot be related to environmental change during the YD. Furthermore, the absence of YD Hg anomalies at White Pond, like the cryptotephra analysis, suggests little to no input of volcanogenic Hg.

## Discussion

### Platinum

Petaev *et al*.^5^ report a large Pt anomaly at the YD onset in the Greenland Ice Sheet Project (GISP2) ice core across an ice interval beginning precisely at the YD onset. They considered multiple potential sources for the Pt anomaly and conclude that the most likely explanation is multiple atmospheric injections of platinum-rich dust by an extraterrestrial impact, followed by fallout of Pt-rich dust during the next 21 years. Geochemical data for meteorites (n = 167), including chondrites, achondrites, irons, and urelites, show Pt abundances ranging from 39,300 to 0.2 ppb (avg: 16,077 to 1198 ppb)^4^. This indicates that all classes of meteorites are possible sources of YDB Pt enrichment. However, Bunch *et al*.^8^ and Moore *et al*.^33,34^ conclude that the evidence is inconsistent with the normal influx of meteoritic dust and consistent only with a rare impact by an asteroid or comet.

A later study by Moore *et al*.^4^ found the same Pt anomaly at the YD onset in 11 widely-spaced sedimentary sequences across North America. A few displayed smaller secondary peaks interpreted as a result of redeposition or depositional variability. Moore *et al*. also show that volcanism is an unlikely contributor to the Pt anomaly given the lack of evidence for continental-scale volcanism at the YD onset, and geochemical studies demonstrating a lack of tephra or sulfur anomalies associated with Pt in the YDB layer (see discussion of Potential Sources of YDB Platinum in SI from Moore *et al*.^4^). At White Pond, analyses of cryptotephra and Hg confirm this and provide no evidence in support of a volcanic source.

As found at many terrestrial archaeological sites by Moore *et al*.^4^, only the YDB layer at White Pond contains both a large Pt anomaly and a coeval Pt/Pd anomaly. The Pt anomaly is ~5.5x higher than the natural Pt background for muddy stratigraphic units (Units I and II) containing the YDB and earlier muddy Pleistocene sediments, and the anomaly is more than 3x background for the entire core sequence tested for Pt, including Holocene peaty sediments that have a slightly higher Pt background level. Pt also is elevated throughout Unit III, likely due to preferential uptake and redeposition of Pt upward in the solum by aquatic plants rooted and intrusive into Unit II, as observed in previous studies^35^. The Pt and Pt/Pd anomalies also display no relationship with fine grain sizes, loss-on-ignition (LOI), total organic carbon (TOC), and mercury (Hg) contents from correlative intervals in core WP-16-3 (Supplementary Table 12). While trace amounts of both Pt and Pd are present at very low levels throughout, the presence of an anomalous ratio of platinum to palladium only in the YDB indicates an influx of Pt-enriched material from a exogenic source at the YD onset^4^.

The combination of Pt, other PGEs, microspherules with scanning-electron microscope (SEM) confirmed melt textures, soot, and/or nanodiamonds, among other impact proxies, found at White Pond and at YDB-dated terrestrial sites on 4 continents suggests an extraterrestrial source via impact and/or atmospheric airbursts^3–18^. The usefulness of the Pt anomaly as a precise chronostratigraphic datum is demonstrated here, and its presence is best explained as the result of an extraterrestrial event.

### Coprophilous fungi

Coprophilous spore frequencies are widely used as indicators of megaherbivore population sizes. For example, declines in *Sporormiella* observed in sediments younger than ~14,800 cal yr BP in Ohio and northern Indiana^27,36^ and New York^37,38^ were interpreted as demonstrating evidence of a sharp decline in megafaunal abundance. Alternatively, it is possible that declining numbers of coprophilous fungal spores may signify microenvironmental fluctuations or a decline in small herbivores. At White Pond, we used multiple taxa of strongly and semi-coprophilous fungal spores to minimize these possibilities^39,40^. All fungi types in this study generally followed the same temporal pattern as *Sporormiella* (see Supplementary Figs 1–4).

Bayesian analysis of 22 AMS dates from cores 2016-1, 2016-2, and 2016-3 reveals a significant hiatus or interval of very slow sedimentation (~2,400 years) immediately above the stratigraphic unit containing the large soot anomaly, Pt and Pt/Pd anomaly, and pre-Holocene *Sporormiella* and strongly coprophilous spore minimum (Fig. 2 and Supplementary Fig. 3). This hiatus occurs at the top of Unit IIB and base of Unit IIIA, with a 1-cm section of core representing a modeled age range of ca. 12,733 to 10,383 cal yr BP (see Fig. 2 and Supplementary Table 2). A sharp decline in *Sporormiella* occurs in Units IIA and IIB; however, given the lengthy hiatus separating Units II and III, we may or may not be seeing the final pre-Holocene *Sporormiella* decline at White Pond that has been attributed elsewhere to megaherbivore extinction (Supplementary Figs 2, 3). On the other hand, at the Page-Ladson site in Florida, the major decline indicating megaherbivore extinction occurs ca. 12,700 cal yr BP^41^. This timeframe is consistent with the observed pre-Holocene minimum in *Sporormiella* and strongly coprophilous spores at White Pond with a Bayesian modeled age of 12,763 to 12,745 cal yr BP (Supplementary Figs 1–3). The Bayesian age/depth statistical model places the upper part of Unit II (containing the soot peak, Pt anomaly, and pre-Holocene spore minimum) as all being deposited during the YD onset—between ca. 12,835 to 12,735 cal BP (Fig. 2 and Supplementary Fig. 3). Based on a recent study from the Page-Ladson site, it appears that the final extinction event did not occur until sometime after the YD onset during the early YD—a time for which we apparently lack a sedimentological record in the White Pond core. Based on evidence from White Pond, Page-Ladson, and other sites^3,41^, we speculate that the proposed YD impact was just one of several coeval factors, along with overhunting and climate change, that contributed to the megafaunal declines at 12.8 ka, followed by a long, multi-century slide into full extinction^41^.

An examination of spore influx (spores/annum) based on the Bayesian age/depth model, shows relatively uniform spore input through most of the late Pleistocene record, with a large spike and then rapid decline to near zero during the YD onset (Supplementary Fig. 3d). One possible interpretation is that there was a period during the YD onset with a sudden increase in megaherbivore abundance at White Pond, followed by a rapid decline represented by the lowest spore abundance in the entire pre-Holocene record. However, there are considerable difficulties with any interpretive model for multiple reasons, including the presence of the hiatus in deposition, uncertainties in the age-depth model, rapid changes in sedimentation, variability in spore preservation, potential sampling and processing errors, and high variability in the lake level. In particular, an episode of drought is suggested by the presence of oxidized sediments in Unit IIb, possibly indicative of subaerial exposure from low water levels and drought across the YD interval. If drought is confirmed, this could explain the hiatus in sedimentation immediately above the YDB layer. All of these issues potentially affect the spore record at White Pond during this time and therefore, spores may not reflect actual megaherbivore abundance. Additional research is necessary to resolve this issue.

### Organic and pyrogenic carbon

In general, the possible routes of entry for soot into a watershed tend to be from fluvial input, local shoreline erosion, and eolian deposition. In the White Pond system, however, the punctuated interval of elevated soot in the absence of a similarly elevated influx of OC, suggests an eolian source. Thus, the presence of a soot anomaly of this magnitude in the White Pond core is consistent with large-scale regional fires coincident with the YD onset. This finding is also consistent with the results reported by Wolbach *et al*.^18^, who presented multiple lines of evidence for large-scale biomass burning on a widespread but discontinuous continental scale at the YD onset.

The largest soot anomaly slightly precedes/predates the Pt and Pt/Pd anomaly in Unit IIA based on nearly identical core lithologies between duplicate cores analyzed for soot and Pt, respectively (Fig. 2 and Supplementary Fig. 11). Under the YDIH scenario, we speculate that this is the order of events at the YD onset: multiple impacts and airbursts occurred over a brief period of time^3,17–20^, along with subsequent climate changes, regional wildfires, an impact winter, and soot deposition immediately after the impact^17,18^. This was followed by deposition of atmospheric Pt over several decades, as indicated by GISP2 ice core data reported by Petaev *et al*.^5^. Furthermore, the chronological sequence in the cores from the pyrogenic carbon peak, Pt anomaly, and extended *Sporormiella* decline is consistent with expected environmental and ecological changes resulting from multiple impacts and airbursts of a fragmented comet or asteroid^3,17–20^. Secondary soot peaks occur in Unit II, including several that overlap stratigraphically with the Pt and Pt/Pd anomalies. Exact correlation by depth for soot peaks and the Pt and Pt/Pd anomalies are impossible due to use of duplicate cores; however, the Bayesian age/depth model for White Pond is consistent with a brief interval of rapid sedimentation in Unit II—implying all proxies could reasonably have been deposited in the core within a ~100-year window (ca. 12,835 to 12,735 cal yr BP).

### C and N isotopes and magnetic susceptibility

At White Pond, sudden increases in carbon and nitrogen content are contemporaneous with decreases in δ^15^N and δ^13^C values at the mud-to-peat transition (Unit IIB to Unit III), indicating a general increase in nutrient availability and primary productivity within and around White Pond (see Supplementary Fig. 13). The concomitant rise in C:N ratios indicates a general increase in terrestrial organic matter (litter) contributions to the SOM pool for White Pond that may have also increased overall N availability. Similar patterns were reported by Spencer *et al*.^42^ and Lane *et al*.^43^ in southeastern North Carolina at roughly this time and are interpreted as a significant increase in terrestrial biomass during the latest Pleistocene and early Holocene as a result of increased moisture delivery to the region. Close correspondence between δ^15^N and magnetic susceptibility during the Pleistocene-Holocene transition supports this interpretation; increased terrestrial biomass likely stabilized soils in the White Pond watershed, thus leading to decreases in allochthonous mineral influx to the core site.

Of relevance to this study, the observed shift in δ^15^N that occurs in the middle of Unit II is interpreted as signaling an overall change in watershed biogeochemical cycles indicative of wholesale ecological transitions concurrent with the Pt and soot anomalies (Supplementary Fig. 13). While the δ^15^N values are not directly indicative of the cause of the YD or subsequent changes, they are direct evidence of dramatic changes in nitrogen cycling in the watershed concurrent with the Pt anomaly.

### Environmental disruption and extinction

A “one-two punch” of human overhunting and rapid climate change has been proposed as a cause of the extinction of Pleistocene megafauna^44–46^. A related possibility is that megafaunal overhunting was directly triggered by the widespread environmental disruption of traditional human food sources through geographically heterogeneous, impact-related biomass burning^17,18^ and by abrupt, impact-related YD climate change. Presumably, some areas less severely affected than others became refugia for remnant herds of megafauna. If so, stressed animal populations in these refugia were more easily targeted by equally stressed human populations, all struggling to survive in the aftermath of an environmental calamity. Afterward, intensive overhunting continued for decades to few centuries as Paleoindians adapted to the sudden loss of vegetative biomass for subsistence. All of these interrelated influences contributed to the final extinction of most of the remaining Pleistocene megafauna across North America before the end of the Younger Dryas (~11,700 cal BP). Small remaining populations of megafauna persisted into the Holocene in isolated refugia away from human predation, before declining into full extinction.

### Hiawatha crater, Patagonia, and YD environmental change

The recent discovery of a massive (31 km) impact crater under the Hiawatha Glacier in northwestern Greenland by Kjaer *et al*.^19^ has garnered great interest because of the presumed recent age. While its exact age is yet to be determined, the crater represents the largest known impact event in the last 5 million years (after Kara-Kul) and the second largest in the last 36 million years (after Chesapeake Bay). If a YD age is eventually confirmed, the Hiawatha impact was energetic enough to have triggered a brief impact winter, abrupt YD climate and oceanographic change, widespread biomass burning, and deposition of multiple impact proxies, as found at more than 50 sites across large portions the globe. In addition, data from Pilauco in Patagonia^20^ add to a growing body of evidence supporting multiple airburst/impacts at the YD onset with synchronous deposition of YDB impact proxies and *Sporormiella* decline. When viewed in the context of rapid environmental change, these new data, along with all previous paleo-environmental reconstructions of the YD, suggest that the trigger was geologically instantaneous. An impact event in the high-latitude Northern Hemisphere (i.e., Greenland) may have been the mechanism to generate the 1^st^- and 2^nd^-order environmental feedbacks within the Earth system that have been identified over the past two decades, suggesting that Earth’s global environment may be sensitive to abrupt (<10^1^ yr) stimuli with lasting effects on the order of at least 10^3^ yrs. As suggested by Moore *et al*.^4^, regardless of whether the YD triggered the observed widespread environmental change, the Pt anomaly at White Pond, as later supported by Bayesian-modeled radiocarbon ages, represents a precise chronostratigraphic datum for the YD onset. Multiple lines of synchronous evidence indicate that a cosmic impact event was a causative mechanism of catastrophic environmental change at the YD onset.

## Conclusions

Bayesian analysis of 22 AMS dates from an ~1-meter section of core obtained from deeply buried lacustrine sediments at White Pond confirm the presence of the YD onset within stratigraphic Unit II dating to ca.12,835 to 12,735 cal yr BP. A large Pt and Pt/Pd anomaly within the YDB unit has a Bayesian modeled age of 12,785 ± 58 cal yr BP and is penecontemporaneous with a large increase in pyrogenic carbon (12,838 ± 103 cal yr BP), that indicates regional biomass burning^17,18^ and significant excursions in δ^15^N indicating rapid climatic and environmental/hydrological changes at this boundary^47,48^. Subsequent declines in coprophilous spores are observed during the YD onset, including a pre-Holocene *Sporormiella* minimum at 12,752 ± 54 cal yr BP; however, a significant core hiatus that spans the later YD to early Holocene precludes a robust assessment of the timing of megaherbivore extinction at White Pond. Immediately after the core hiatus, spore abundance increases again during the early Holocene. A secondary spore concentration peak occurs in these post-YD, early Holocene-age sediments (ca. 10,335 ± 58 cal yr BP) also observed at Page-Ladson, may have resulted from bison filling an ecological niche vacated by the extinction of other megaherbivores^41^. The penecontemporaneous nature of the Pt and Pt/Pd anomalies and pyrogenic carbon peaks occurring together in Unit II at White Pond are consistent with an extraterrestrial impact event that triggered widespread biomass burning, as observed globally and reported elsewhere during the YD; however, the severity of environmental disruption at White Pond, its role in local megaherbivore extinction, and its impact on human life are yet to be determined. Cryptotephra and Hg analyses suggest no relationship between increased volcanic activity and the observed Pt and Pt/Pd anomalies. At White Pond, the synchronicity of multiple lines of evidence is intriguing and deserves further investigation into causality. In summary, the combination of proxy evidence within core sediments currently supports the cause-and-effect linkage of an extraterrestrial impact with large-scale regional biomass burning, abrupt YD climate change, and megafauna declines leading to eventual extinction.

## Materials and Methods

In 2016, multiple duplicate cores were collected from White Pond from the same general vicinity as that tested by Krause *et al*.^2^ (Supplementary Figs 5–9). Sediment cores were recovered using a vibracore tripod system and 4-in. core barrels extracted with a come-along winch. Core depths varied but ranged from 2.41 to 3.28 m below the sediment/water interface. Duplicate cores for various analyses were correlated using the peat to mud transition and lithologic Unit II (common to all cores) as a common datum for sample reference (see Supplemental Fig. 9). All cores were cut into subsections and stored in refrigeration prior to splitting lengthwise. Each core was then photographed, described lithologically, and subsampled for various analyses. A brief description of specific analytical methods is provided below. Core samples for most analyses were collected in 2-cm continuous intervals. Samples for high-resolution AMS dating were collected directly from split cores or were collected by wet-sieving of the 2-cm interval subsamples.

### Radiocarbon dating and Bayesian age/depth model

In all, a total of 30 AMS radiocarbon dates for all cores (2015 and 2016) were obtained for this study from bulk sediment, seeds, charcoal, wood, and plant remains (Supplementary Table 1). Twenty-four dates were provided by Beta Analytic, Inc., Miami, Florida, and six from the W. M. Keck Carbon Cycle Accelerator Mass Spectrometry Laboratory, University of California, Irvine. The initial dates (n = 7) were obtained on a core collected in 2015 (Supplementary Fig. 7) in order to identify the time frame of interest. Following this, larger vibracores were obtained and more intensively dated (n = 23) (see Supplementary Fig. 8). One sample produced a modern date from the very bottom of core 2016-3 that was likely due to contamination immediately after exhuming the core from the lake. Omitting this date from the analysis, 22 AMS dates were utilized from cores 2016-3 (n = 19), 2016-2 (n = 2), and 206-1 (n = 1). These dates were calibrated within the OxCal program 4.3.2, using the Northern Hemispheric calibration curve, and then use to create a Bayesian age-depth model. Uncertainties were calculated at confidence intervals (CI) of 68% and 95%. Of the 22 AMS dates, 19 were accepted for inclusion in the Bayesian age/depth model (Supplementary Table 2).

### PGE analysis

Geochemical analyses of sediment samples for PGE concentrations across the YDB were conducted by Actlabs Inc. using fire-assay and inductively coupled plasma mass spectrometry (FA and ICP-MS) elemental analyses to determine the presence or absence of the Pt anomaly observed elsewhere at terrestrial archaeological sites across North America^4^ and Greenland^5^. In all, 33 samples were tested for Pt, Au, and Pd from samples collected from core 2016-3 (see Supplementary Information, “PGE Analysis” and Supplementary Figs 9, 10, and Supplementary Table 3).

### Palynology

1 cm^3^ of sediment was processed and analyzed from each of the 35 samples which were collected from Core WP 2016-3. After the addition of 3 *Lycopodium* tablets containing 9,666 tracer spores each, the samples were heated for 10 minutes to 80 degrees C in 5% KOH to remove humates. Screening through 250-micron and 150-micron screen followed. After decanting supernatant liquid, 48% HF was added for 24 hours, followed by an HCl wash. The samples were then subject to Acetolysis with a 9:1 Acetic Anhydride: Sulfuric Acid solution for 10 minutes at 80 degrees C. Each sample was then screened through 70-micron mesh, stained, and curated in glycerine. Samples were mounted on slides and scanned using light microscopy at 40x for 20 transects. *Lycopodium, Cercophora, Coniochaeta, Podospora, Sordaria*, and *Sporormiella* were tallied (see Supplementary Information; “Coprophilous Spores” and Supplementary Figs 1–6 and Supplementary Tables 4, 5).

### Organic and pyrogenic carbon

Soot, a pool of pyrogenic carbon consisting of condensed aromatic molecules, was also analyzed from a duplicate core (2016-1) collected immediately adjacent to separate cores (2016-2 and 2016-3) collected for Pt and coprophilous fungi analyses (Supplementary Figs 9, 11). Sediments collected from WP2016-1 were acidified dropwise with 2 N HCl until effervescence ceased. Subsequently, chemo-thermal oxidation (375 degrees C for 24 h) was used to isolate just the soot portion of pyrogenic carbon. Soot carbon quantification and isotopic values were determined in a manner similar to that of OC (Supplementary Table 8).

Ten samples from White Pond were tested for aciniform carbon/soot from WP2016-3 (Supplementary Fig. 9). A standard protocol was used to extract AC/soot from bulk sediment^17,18^. The process consists of multiple steps: (1) demineralization through treatments of hydrofluoric acid (HF) and hydrochloric acid (HCl); (2) oxidation to separate organic material from elemental carbon by using sodium dichromate; (3) SEM analysis to differentiate between AC/soot and non-soot elemental carbon; and (4) quantitative examination of SEM micrographs to determine ratios of soot to non-soot particle residue components.

### Stable C and N isotope analysis of sediments

Homogenized sediments were transferred to tin capsules and combusted in a Thermo Flash HT Plus or Costech 4010 elemental analyzer interfaced with a Thermo Delta V Plus stable isotope mass spectrometer at the University of North Carolina Wilmington (UNCW). Carbon and nitrogen isotopic signatures (δ^13^C and δ^15^N) of the samples were expressed in the standard delta unit notation (δ) as δ^13^C or δ^15^N (‰) = [(Rsample/Rstandard) − 1)] × 1000, where R is ^13^C/^12^C or ^15^N/^14^N, respectively. Repeated measurements of USGS 40 and 41 glutamic acid standards indicate that the precision of these analyses is better than 0.3‰. Results of this analysis are shown in Supplementary Fig. 13 and Supplementary Table 9.

### Cryptotephra

Contiguous 2-cm samples from White Pond (see Supplementary Fig. 9) were sieved between 80 and 25 μm to remove coarse particles >80 μm and obscuring silt and clay <25 μm. Each >25 μm fraction was then subjected to the centrifuge density flotation method of Blockley *et al*.^49^ using lithium heteropolytungstate (LST) to float out rhyolitic volcanic glass shards from the relatively heavier background mineral assemblage. Floated residues were then mounted onto slides and inspected by light microscope for the presence of glass shards.

### Magnetic susceptibility

Thirty-five samples, containing the YD interval, were collected from core WP2016-3 at 2.32 cm intervals between 180 and 260 cm depth (Note, these are the same samples used in the spore analysis) (Supplementary Fig. 9). Sample volume was 7 cc with an average Mass of 8.5 g. Magnetic susceptibility was measured with a Bartington MS2B magnetic susceptibility meter at 0.465 kHz ± 1% (low frequency). Both K (Volume Specific Susceptibility in 10^−3^ SI units) and X (Mass Specific Susceptibility in 10^−6^ m^3^ kg^−1^ units) were calculated (see Fig. 2 in main paper and Supplementary Fig. 14). To improve the signal to noise ratio, a moving average filter of three consecutive measurements was applied to the data.

### Elemental geochemistry (Ultratrace 4)

Continuous 2-cm samples (n = 17) were collected from WP2016-3 and sent to Actlabs for Ultratrace 4 (“Near Total” Digestion-ICP/MS) testing. The section of the core tested is shown in Supplementary Fig. 9 and Supplementary Table 10). Samples were selected to bracket the YD onset interval in Unit II and include continuous sampling through portions of Units III and I. A 0.25 g sample is digested with four acids beginning with hydrofluoric, followed by a mixture of nitric and perchloric acids, heated using precise programmer-controlled heating in several ramping and holding cycles which takes the samples to dryness. After dryness is attained, samples are brought back into solution using hydrochloric and nitric acids. This digestion may not be completely total if resistate minerals are present. As, Sb, and Cr may be partially volatilized. An in-lab standard (traceable to certified reference materials are used for quality control. Digested samples are diluted and analyzed by Perkin Elmer Sciex ELAN 6000, 6100 or 9000 ICP/MS. One blank is run for every 40 samples. In-house control is run every 20 samples. Digested standards are run every 80 samples. After every 15 samples, a digestion duplicate is analyzed. The instrument is recalibrated every 80 samples. The Ultratrace 4 analysis provides ppm detection limits for 58 elements.

### Hg

All mercury (Hg) analyses were performed at the Geochemistry of Ancient and Modern Environmental Systems (GAMES) Laboratory at the College of Charleston. Briefly, samples from core WP-2016-3 were dried in a gravity oven at low temperature in order to minimize Hg volatilization. Samples were then lightly ground and homogenized using a mortar and pestle. Approximately 50-100 mg of each powder were added into a nickel measuring boat and then placed into a DMA-80 (Milestone, Inc., Shelton, CT). Samples were heated in stages, and volatilized Hg was collected by gold amalgamation and measured via spectral analysis. The raw Hg data were calibrated using international reference standards (DORM-4, and TORT-3). Approximately 10% of the samples were replicated at least two times, and the average 2-sigma standard deviation of these replicate analyses is ±0.003 mg/kg or ppm (3 ppb), with a range of 0.000 to 0.004 mg/kg or ppm (0 to 4 ppb) (see Supplementary Table 11).

### Grain size analysis

An approximately 0.1- to 0.5-g aliquot of each sample was placed in a small vial with approximately 2 ml of deionized water and disaggregated for approximately 30 seconds using a Virtas Virsonic 475 Sonicator at 25% power setting. The sample was then analyzed for particle distributions using a Cilas 1180 laser particle size analyzer (PSA).

The system is drained, flushed, and rinsed three times at the start of each analytical cycle. After cleaning the system, a background reading was taken prior to each sample being introduced. Once introduced to the PSA, the sample was stirred, and ultrasound was applied for dispersion for 60 seconds, and then measured for 90 seconds to gather approximately 10,000 readings in the instrument. The resulting measurement provides 100 classes or bins from 0.04 to 2500 microns and calculates key grain size statistics within the program. For the purposes of this paper, the d50 (median) value is presented and there has been no differentiation between inorganic, calcitic, and organic constituents.

### LOI

Samples were taken at 2-cm intervals from cores WP2016-1 and WP2016-3. These were placed in a pre-weighed ceramic crucible and dried at 80 °C for 48 hours. They were then weighed (to 0.1 mg) and combusted in a muffle furnace at 550 °C for 4 hours, then allowed to cool to 80 °C and weighed again. Percent LOI is calculated as (dry sample wt. − combusted wt./dry sample wt.) × 100.

## Supplementary information

Supplementary Information

## Data Availability

All data needed for the evaluation of this paper are present in the paper and/or Supplementary Information. Additional data related to this paper may be requested from the authors.
