# Supplementary material for: Sediment Cores from White Pond, South Carolina, contain a Platinum Anomaly, Pyrogenic Carbon Peak, and Coprophilous Spore Decline at 12.8 ka
Source: Sci Rep. 2019 Oct 22;9:15121. doi: 10.1038/s41598-019-51552-8 (PMC6805854; doi:10.1038/s41598-019-51552-8)
Supplement: Supplementary file 1 — Supplementary Information [file 41598_2019_51552_MOESM1_ESM.pdf]

**Supplementary Information:** ***Sediment Cores from White Pond, South Carolina, contain a Platinum Anomaly, Pyrogenic Carbon Peak, and Coprophilous Spore Decline at 12.8 ka***

Christopher R. Moore,[1]* Mark J. Brooks,[1] Albert C. Goodyear,[2] Terry A. Ferguson,[3] Angelina G. Perrotti,[4] Siddhartha Mitra,[5] Ashlyn M. Listecki,[6,7] Bailey C. King,[6,7] David J. Mallinson,[5] Chad S. Lane,[8] Joshua D. Kapp,[9] Allen West,[10] David L. Carlson,[11] Wendy S. Wolbach,[12] Theodore R. Them II,[13] M. Scott Harris,[13] and Sean Pyne-O'Donnell[14]

[1]Savannah River Archaeological Research Program, South Carolina Institute of Archaeology and Anthropology, University of South Carolina, P.O. Box 400, New Ellenton, SC 29809, USA. [2]South Carolina Institute of Archaeology and Anthropology, Columbia, SC 29208, USA. [3]Department of Environmental Studies, Wofford College, 429 N Church Street, Spartanburg, SC 29303-3663, USA. [4]ORCID: 0000-0001-6003-0082, University of Wisconsin, Geography Department, 550 N Park Street, Madison, WI 53707-1404. [5]Department of Geological Sciences East Carolina University, Greenville, NC 27858-4353. [6]Department of Biology, East Carolina University Greenville, NC 27858-4353. [7]Department of Chemistry, East Carolina University, Greenville, NC 27858-4353. [8]Department of Earth and Ocean Sciences, University of North Carolina Wilmington, NC 28411. [9]Department of Ecology and Evolutionary Biology, University of California, Santa Cruz, CA 95064. [10]Comet Research Group, Prescott, AZ USA. [11]Department of Anthropology, Texas A&M University, College Station, TX 77843-4352. [12]Department of Chemistry, DePaul University, Chicago, IL 60614. [13]Department of Geology and Environmental Sciences, College of Charleston, Charleston, SC 29424. [14] Earth Observatory of Singapore and Asian School of the Environment, Nanyang Technological University, Singapore.

*To whom correspondence should be addressed. E-mail: MOORECR@mailbox.sc.edu

## Materials and Methods

**Bayesian Analysis (see main manuscript and Supplementary Tables 1 and 2)**

**PGE Analysis**

Activation Laboratories (Actlabs), using fire-assay (FA) and inductively coupled plasma mass spectrometry (ICP-MS) after Hoffman and Dunn[1], measured the elemental concentrations of sediment samples from all sites. Core samples were collected at 2-cm intervals and tested using the "1C-Research" analysis. In order to provide a large sample size for the analysis, both halves of the core (2016-2) were combined. Prior to analysis, each sample is mixed with fire assay fluxes (borax, soda ash, silica, litharge) and silver (Ag) added as a collector. The mixture is placed in a crucible and preheated at 850°C, intermediate at 950°C, and finished at 1060°C for a total of 60 minutes. After the crucibles are removed from the assay furnace, the molten slag is poured into a mold leaving a lead button. The lead button is then preheated to 950°C to recover the Ag (doré bead) + Au, Pt and Pd.

The Ag doré bead is digested in hot (95°C) $HNO_3$ + HCl with a special complexing agent to prevent the Au, Pd, and Pt from adsorbing onto the test tube. After cooling for 2 hours the sample solution is analyzed for Au, Pt, and Pd using a Perkin Elmer Sciex ELAN 9000 ICP-MS. On each tray of 42 samples, there are 2 method blanks, 3 sample duplicates, and 2 certified reference materials. The ICP-MS is recalibrated every 45 samples. Smaller sample splits are used for high chromite or sulfide samples. Measurements are reported in parts per billion (ppb) with a lower limit of detection for Pt at 0.1 ppb (see Supplementary Table 3).

## Coprophilous Fungi

**1. Background**

Owen Davis[2] first suggested using the fossil spores of coprophilous fungi (also referred to as “dung fungi”), *Sporormiella,* as a proxy for North American herbivore abundance and disappearance at the end of the Pleistocene. Davis[2-3] recognized the potential of coprophilous fungi in paleoecology when he found *Sporormiella* spores in direct association with fossilized dung from Bechan Cave, Utah, and also noted the frequency of *Sporormiella* spores increased

with evidence of overgrazing of domestic livestock in historic-age deposits at Wildcat Lake in Washington State.

Some researchers have even suggested that declines in dung fungi can indicate human colonization, citing evidence that extinction events worldwide have followed human settlement into a new area[4-6]. However, Fiedel[7] refutes that due to problems in both cultural chronology and inconsistencies between the dung fungi and faunal records. For example, declines in *Sporormiella* observed in sediments younger than ~14,800 cal yr BP from Ohio and northern Indiana[8,9] and New York[10,11] were interpreted as demonstrating a sharp decline in megafaunal abundance, followed by extinction or near extinction within 1000 years. However, Fiedel[7] points out that a number of megafaunal remains were recovered from the Northeastern United States dating between 10,000 and 12,000 rcybp (~12,000-14,000 cal yr BP).

Despite disparities such as these, in the nearly three decades that *Sporormiella* and other dung fungi have been used as proxy evidence for megafauna extinctions[3,5,8,12], methodologies have become increasingly reliable, and dung fungi have been demonstrated to reflect relative large herbivore abundance and activity in many modern settings[13-16].

Although less commonly incorporated into paleoecological studies, other coprophilous fungi taxa are frequently observed alongside in *Sporormiella*, often in even higher concentrations. These other fungi types, including *Cercophora, Conichaeta, Podospora,* and *Sordaria* (see Supplementary Table 4) are not necessarily obligate to herbivore dung[17-18] but have still been deemed as indicators of herbivore populations[19]. Counting the abundance of these fungi alongside *Sporormiella* may provide a more complete picture of ancient herbivore abundance and disappearance, as different fungi may have species-specific reactions to microenvironmental fluctuations.

## 2. Palynological Methods

One $cm^3$ of sediment was processed and analyzed from each of the 35 samples received by C. Moore in 2016, which were collected from Core WP 2016-3 in 2.32 cm diameter plastic cubes (see samples cubes from core image in Fig. 2 in the main paper). After the addition of 3 *Lycopodium* tablets containing 9,666 tracer spores each, the samples were heated for 10 minutes to 80° C in 5% KOH to remove humates. Screening through 250-micron and 150-micron screens followed. After decanting supernatant liquid, 48% HF was added for 24 hours, followed by an

HCl wash. The samples were then subject to Acetolysis with a 9:1 Acetic Anhydride: Sulfuric Acid solution for 10 minutes at 80° C. Each sample was then screened through 70-micron mesh, stained and curated in glycerine. Samples were mounted on slides and scanned using light microscopy at 40x magnification for 20 transects. *Lycopodium, Cercophora, Coniochaeta, Podospora, Sordaria,* and *Sporormiella* were tallied.

Concentrations (spores/$cm^3$) of each dung fungi taxa were then calculated using the following formula:

(Spores Counted x [9,666*3])/ *Lycopodium* counted

All dates are presented and discussed in modeled calendar years before present (Supplemental Table 2). These were determined by adding the length of each sample cube to the provided starting core depths. Modeled ages were provided to me for every 0.5 centimeters. Each of these values was then grouped into original sample numbers (1-35). .5 centimeter values were rounded when they did not fall discretely into sample values. Each age within a sample was then averaged.

Fungal spore types are grouped based on how strongly each taxon's abundance reflects megaherbivore presence. *Podospora, Sporormiella,* and *Sordaria* are strongly coprophilous. The total spore concentration of these two taxa is presented in Supplemental Figure 1. Other types such as *Coniochaeta* and *Cercophora* may not be strong indicators of megaherbivore presence (e.g. Perrotti and van Asperen[18]).

## 3. Results

Based on visual comparison of images of Core WP 2016-3 to the 2015 core and sedimentary units, I have divided up WP 2016-3 into 4 spore zones. Based on Bayesian modeled median ages (Supplemental Table 5), samples 35-24 (263 to 234.4 cm) are in Zone I, which dates from ~14,800-13,300 cal yr BP. Spore sample 23 straddles the contact of lithostratigraphic Unit I and Unit II, which includes spore samples 23-15 (232 to 213 cm). This zone dates from ~13,200-12,700 cal yr BP. Although top and bottom spore zone boundaries were determined using the modeled age based on the depth of the top of the upper sample cube, and the modeled age of the bottom of the sample cube in each zone, modeled sample midpoint depth was used to

determine the age for the top of Zone II (spore sample 15). This is because there is evidence of a ca. 2,400-year hiatus in sedimentation between spore samples 15 and 14, between 212-211 centimeters in core 2016-3. Samples 14-10 (210.04 to 200.08 cm) are in Zone III, which dates to ~10,400-10,300 cal yr BP. Once again, the bottom age boundary for this zone was determined by taking the modeled age for the midpoint of sample 14, due to a probable hiatus in sedimentation directly below this sample. Zone IV includes spore samples 9-1 (198.6 to 178.5 cm) and extends to the top of the section, dated to ~9,900 cal yr BP. It should be noted that zones were used for spore data visualization and may not reflect concrete patterns of megaherbivore abundance and decline, based on differences in lithology throughout the core.

3.1 *Zone I (>13,300 cal yr BP)*

All fungal spores remain profuse throughout this zone. Strongly coprophilous *Sporormiella* (Supplemental Figure 1 and Supplementary Table 5) and *Podospora* are both abundant, with a total concentration of up to 16,000 spores/$cm^3$. Total spore concentration reaches 36,000 spores/$cm^3$.

3.2 *Zone II (13,300-12,700 cal yr BP)*

Fungi concentrations remain moderate to high throughout this zone. Total spore concentration remains high primarily due to a peak in *Coniochaeta*. *Sporormiella* and *Podosopora* abundance is more variable than in Zone II. Total spore concentration declines toward the end of this unit, primarily as a result of declining *Coniochaeta* and *Podospora. Sporormiella* and strongly coprophilous spores exhibit a pre-Holocene minimum near the top of this zone at the YD onset and just before a core hiatus discussed in the main paper.

3.3 *Zone III (10,400-10,300 cal yr BP)*

This zone is characterized by rapid sedimentation, after a probable hiatus in sedimentation. Throughout this zone, total spore concentration remains low, despite the rapid sedimentation rate. Total fungal spore concentration reaches a high of only ~10,000 spores/$cm^3$. At the end of this zone, strongly coprophilous fungal spores are nearly absent.

3.4 *Zone IV (10,300-9,900 cal yr BP)*

This zone marks a slight increase in total spore concentration but is likely due to an increase in *Coniochaeta*. Although strongly coprophilous fungal spore abundance remains relatively low, a small peak near the top of the section in conjunction with increased *Coniochaeta* and *Cercophora* may indicate an increase in herbivore population.

## 4. Discussion

Although dung fungi as a proxy for large herbivore abundance are often better understood in the context of the ancient plant community, we are unable to make inferences about past vegetation based on the existing studies[20-21] due to discrepancies between age-depth models. It is also not possible to accurately correlate interpretations based off depth since the analyses were completed on different cores.

In Zone I, total spore concentration is high, despite each taxon having seemingly independent fluctuations in concentration (Supplemental Figure 1 and Supplementary Table 5). *Coniochaeta* and *Sporormiella* are most abundant, followed by *Podospora.* Zone II is characterized by moderate-high total spore concentrations. Because total spore concentration is moderate, and *Coniochaeta* (which is also associated with decaying wood, in addition to herbivore dung)[17] is the only fungi with consistently high concentration, this could represent a decline in herbivore population during this unit during the YD onset. A pre-Holocene minimum in *Sporormiella* and strongly coprophilous spores occurs in Zone II (spore sample 16) and has a modeled age of 12,763 to 12,745 cal yr BP. This is consistent with the timing of megafaunal extinction elsewhere in North America. For example, sediments from Page-Ladson, Florida demonstrated a dramatic decrease in *Sporormiella* between 12,600-12,700 cal yr BP[22-23] (Supplementary Figure 2 and 3). At White Pond, however, the end of this zone (Zone II) coincides with a ca. 2,400 year hiatus in sedimentation immediately following the YD onset and continuing until the Early Holocene, so changes in spore abundance during this time are unknown (Supplementary Figures 1-3).

Additionally, it is unclear precisely when the sedimentary hiatus began and ended, resulting in uncertainty regarding the time period over which the two samples immediately preceding and following the sedimentary hiatus were deposited. The modeled total time of deposition for each sample immediately above and below the sedimentary hiatus is likely too high, due to this uncertainty. Therefore, spore influx estimates for these samples are likely artificially low because spore concentration for these samples is minimized due to the extremely slow sedimentation rate predicted by the age-depth model. Especially for these samples, spore concentration is likely a more accurate reflection of total spore abundance. Regardless, concentration values for Sporomiella and strongly coprophilousas spores show a decline to a pre-Holocene minimum during the YD onset (Supplementary Figure 3).

Zone III consists of low total spore concentration. After 10,400 cal yr BP, total spore concentration does not rise above 10,000 spores/$cm^3$, which may reflect a near absence of large herbivores at White Pond. Zone IV marks a slight increase in total spore concentration but is primarily due to an increase in *Coniochaeta*. Although strongly coprophilous fungal spore abundance remains relatively low, a small peak near the top of the section in conjunction with increased *Coniochaeta* and *Cercophora* may indicate an increase in herbivore population.

Ultimately, the fungal spore record from White Pond could be used in conjunction with studies on the herbivore faunal record. Although the presence of fungal spores is likely to reflect large herbivore abundance in the region, we cannot draw any strong conclusions about the timing and significance of the pre-Holocene decline of dung fungal spores at White Pond.

## Magnetic Susceptibility

In the study of Environmental Magnetism as it relates to lacustrine sediments, magnetic susceptibility is often a good indicator of changes in composition, texture, depositional history, and climate change. In the White Pond core, magnetic susceptibility provides a good proxy for these core characteristics and formation processes. Even though the susceptibility readings were relatively weak with relatively lower levels of magnetic minerals combined with a negative diamagnetic component there are some clear patterns in the data (see Figure 12). The lowest susceptibility readings are associated with Unit III (sub-units IIIc, IIb, IIIa) which are predominated by peat (a diamagnetic substance). Unit III also has the lowest average density of 1.11 g/cc. The mud (silt and clay) Units II and I exhibit increasingly higher susceptibility with marked increases at the transitions between units. Unit II has a higher average density at 1.21 g/cc and Unit I (sub-units IIID and IIIC) have the highest average densities at 1.32 g/ $cm^3$. Unit I (sub-unit IIIB) has a density of 1.26 g/$cm^3$.

Bayesian modeling of radiocarbon dates from the core indicates Unit II contains the deposits dating to the YD interval. The two most marked decreases in magnetic susceptibility are between Unit I to Unit II and Unit II and Unit III. These decreases in susceptibility might indicate significant reductions in the input of magnetic minerals into the sediments or changes in the chemical processes affecting magnetic minerals within the sediments. The reductions also suggest the possibility of climatic shifts at these boundaries. There is an increase in susceptibility

during the later portion of the YD interval. In Fig. 2 in the main paper, it can be seen that this increase mirrors an increase in $\delta^{15}N$. The marked correspondence in general between $\delta^{15}N$ and magnetic susceptibility during the Pleistocene-Holocene suggest that as increased terrestrial biomass may likely have stabilized soils in the White Pond watershed leading to decreases in allochthonous mineral influx to the core site.

Further study of other magnetic properties such as hysteresis and remanence, along with Mossbauer analysis will be required to refine the interpretations suggested in the present investigations.

## Elemental Geochemistry

Using the Ultratrace 4 analysis performed by Actlabs Inc., sedimentary abundances were obtained for 58 elements in 17 samples (core 2016-3) that bracket the YDB (see Supplementary Figure 9 for sample locations). Results are presented in Supplementary Table 10. Among the more interesting findings, 6 elements (Na, Fe, As, Nb, Cu, and Ta) have anomalies directly or closely associated with the Pt anomaly and YDB zone (see Supplementary Figure 15). Similar anomalies were also reported in the YDB layer in North America and Europe by Firestone *et al.*[24-25]. The causes are unclear, but we speculate that most result from environmental disruptions caused by the impact event; some, such as Pt, may derive from the impactor.

We also measured Ni concentrations but did not observe anomalously high concentrations in the YDB layer (see Supplemental Table 10). This is consistent with Firestone *et al.*[25], who note that Ni concentrations at 10 YDB sites ranged from below detection to 256 ppm.

We considered whether this high geochemical variability can be considered as evidence against a single impactor or a single impact event, based on the proposition that a single impactor could have homogeneous concentrations of Ni and Pt. However, high variability is typical of known impact sites, such as at the KT boundary, where Ni concentrations in the impact layer vary from below detection to 5.9 ppm, below crustal abundance (35 ppm), up to 1278 ppm, a difference of >200× (Table 1 in Goderis *et al.*[26]). In addition, Pt varied from 0.25 ppb, also below crustal abundance (0.5 ppb), up to 81.09 ppb, a difference of >300× (Table 1 in Goderis *et al.*[26]). There also can be very high variability among meteorites. Moore and West[27]

report Pt concentrations for >150 meteorites. Some are relatively heterogeneous, but others are not. For example, the Cumberland Falls meteorite has Pt concentrations ranging from 120-6700 for a difference of 56×; Mayo Belwa ranges from 0.7-3600 ppb, differing by >5000×; and Pt in Mt. Edgerton ranges from 3.7-5200 for a difference of>1400×.

Many factors can produce highly variable concentrations of Ni, Pt, and other impact-related elements, including composition of the impactor, potential fractionation during vaporization and condensation, terrestrial mineral input, sedimentation rate, reworking, diagenesis, bioturbation, and chemical diffusion. Therefore, high geochemical variability is not an argument against either a single impactor or a single impact event.

## Cryptotephra

Only single shards were found in three, 2-cm sample intervals from core 2016-3. These include sample intervals 245–247 cm, 239–241 cm, and 226.5–228.5 cm intervals. All three shards were clear glass with fluting and vesicles. There is no evidence of a discrete cryptotephra ash layer.

## Grain Size

Forty-eight samples were received for grain size analysis. Of these, ten (~20%) were randomly selected for subsampling. Seventy (n=70) analytical runs were completed for this dataset. The first sample was run three times to make sure the system was running properly and that the samples were correctly behaving in the system.

General grain size distribution and ranges are displayed in Supplementary Figure 16. The most prominent changes in grain size occur at the transitions between Unit I, Unit II, and Unit III, which represent lithological variations.

## Hg, Loss-on-Ignition (LOI), Total Organic Carbon (TOC), and Grain Size

The Hg and Hg/LOI and Hg/TOC values were explored to determine if a perturbation of the mercury (Hg) cycle occurred across the YD (see Supplementary Figure 17). Although Hg

values increased across the YD interval, this was driven by local increases in carbon content (i.e., there is no perturbation to Hg/LOI or Hg/TOC; see Supplementary Figure 18). Therefore, the raw Hg values were associated with lithology.

Mercury contents were compared to grain sizes to determine if there was any relationship (see Supplementary Figure 19). The strongest relationship occurred between Hg and grain size in the D10 and D50 fractions ($R^2$ = 0.25 and 0.23, respectively), but there was also a lesser correlation in the D90 fraction ($R^2$ = 0.20) if three anomalous grain sizes are not included. Their inclusion decreases the correlation significantly ($R^2$ = 0.04).

Loss-on-ignition values were also compared to grain sizes to determine if there was any relationship (see Supplementary Figure 20). The strongest relationship occurred between LOI and D10 ($R^2$ = 0.76), with two populations observed. There are also strong relationships between LOI and D50 ($R^2$ = 0.67) and LOI and D90 ($R^2$ = 0.40; $R^2$ = 0.13 if the three anomalous D90 values are included). The two populations of data represent different lithologies (e.g., mud-dominated versus peat-dominated), but nothing more can be determined.

Mercury/LOI values were also compared to grain size to determine if any relationship exists (see Supplementary Figure 20). There is a strong relationship between Hg/LOI and the D10 fraction ($R^2$ = 0.47; power-law correlation) and also between Hg/LOI and D50 ($R^2$ = 0.38; power-law correlation). The same trends are observed with Hg/TOC and grain size. These relationships suggest that sedimentary Hg/LOI and Hg/TOC anomalies may be related to the relative contribution of fine grains. Therefore, studies that document sedimentary Hg and Hg/TOC anomalies should also generate grain size data to rule out the potential control of minor variations in grain size. Furthermore, as many studies focus on ancient time intervals with distinct lithological variations and facies changes associated with Hg and Hg/TOC anomalies, it may become standard (and necessary) to generate ancillary grain size data.

## Sedimentary Ancient DNA

To further explore changes in faunal biodiversity of White Pond over time, we attempted to extract and characterize environmental DNA from several layers throughout the cores. Although the setting of White Pond in a warm and wet environment is not ideal for the preservation of ancient DNA[28], we were motivated to attempt ancient DNA recovery based on

the preservation of large amounts of fungal DNA spores, which indicated a mammalian presence in the ecosystem.

To prevent contamination of the samples by exogenous sources of DNA, we performed all DNA processing steps in our purpose-built ancient DNA facility at the UC Santa Cruz Paleogenomics Laboratory, using sterile protocols developed for ancient DNA research[29]. We processed a total of 25 sediment samples, following two DNA extraction protocols that have been optimized for the recovery of ancient sediment DNA. First, we attempted to recover DNA using the DNeasy Powersoil Kit (Qiagen) using 250mg of sediment from each sample, and eluting into 50uL of buffer EBT (10mM Tris-HCl, 0.05% Tween-20). After processing these extracts as described below and failing to recover sufficient DNA to pass our authentication criteria, we re-extracted DNA from 9 of the 25 samples following the protocol described in the supplement of Seersholm *et al.*[30], which has been suggested to be more efficient than the Powersoil Kit in recovering DNA from some types of sediment. These second extractions used 500mg of sediment per extraction.

After the first round of DNA extractions, we transformed a fraction of each of the 25 DNA extracts into Illumina sequencing libraries using a single-stranded approach similar to that described by Gansauge *et al.*[31]. We then attempted to enrich these libraries for mammalian mitochondrial DNA using an approach described by Slon *et al.*[32], and a bait set (synthesized by Arbor Biosciences) comprising mitochondrial genomes from 179 Late Pleistocene and Holocene mammals from North America, as described in Kirillova *et al.*[33]. We sequenced the enriched libraries on an Illumina NextSeq, generating between 80 and 530 thousand reads per enrichment (Supplementary Table 6).

Because our bait set only captured mitochondrial DNA, we performed an additional set of experiments designed to test for the presence of mammalian nuclear DNA. We prepared eight additional single-stranded libraries from select DNeasy PowerSoil extracts and single-stranded libraries from the nine Seersholm protocol extracts. We then sequenced these 17 shotgun libraries on an Illumina X Ten, generating between 9.5 and 37 million reads per library (Supplementary Table 7).

To process the recovered sequence data, we first removed adapted dimers and all sequences shorter than 30 base-pairs (as these would not be uniquely mappable to a reference nuclear genome with high confidence) from each library and then merged overlapping reads

using SeqPrep (available from https://github.com/jstjohn/seqprep). We then removed low complexity reads from each library using PrinSeq[34]. We mapped each remaining read using BWA[35] from each of the 17 shotgun libraries separately to 20 mammalian nuclear genomes, which we selected based on genome availability and evolutionary similarity to taxa that are likely to be preserved in the White Pond sediments (mouse, human, cattle, horse, bison, mammoth, two-toed sloth, Bactrian camel, beaver, bighorn sheep, goat, armadillo, caribou, domestic dog, polar bear, white rhino, western roe deer, domestic cat, African elephant, and pig). For sequence data generated from the enriched libraries, we mapped each read to the corresponding mitochondrial genomes. Following mapping, we removed duplicate reads from individual mapped files using samtools[36], so as to retain only uniquely mapped reads to each reference used. Uniquely mapped reads were locally aligned to a non-redundant nucleotide database using BLASTn to confirm species identity.

Unfortunately, among the 17 shotgun libraries and 25 enriched libraries, no reads were confidently mapped to a non-human mammalian genome (Supplementary Tables 6 and 7). This result reflects the poor state of DNA preserved in the White Pond core samples. The major component of all libraries was prokaryotic with, as is expected with poorly preserved ancient sediment samples, a small number of human reads passing all filtering steps.

## Critical Overview of YDIH

The following is adapted from Pino *et al.*[41].

First proposed in 2007[24], the YDB impact hypothesis is still controversial a decade later because a number of independent studies have raised questions about the proposed YDB impact event. The authors of several studies[42-44] have argued that dating accuracy and precision are insufficient to determine whether YDB proxies are coeval across the many sites. (2) Five of 13 independent studies were unable to confirm the presence of peaks in YDB magnetic spherules. (3) Three studies[42-44] noted that sediment sampling at YDB sites is typically discontinuous, except at and around the YDB layer, where sampling spanned at most a few thousand years, making it difficult to know whether the "impact assemblage" of indicators is unique to the YDB. (4) In one study[42], it was argued that the proposed YDB impact mechanism of a fragmented comet is so rare as to be statistically implausible. (5) Several investigators[44-47] proposed that the rate and timing of

megafaunal extinctions were not simultaneous but varied across the continents, with a large percentage of them occurring prior to the YDB. (6) Another study[48] concluded that a major peak in wildfire activity preceded the YD onset by several hundred years and did not correlate with the YD onset. (7) It was argued in several studies[42,44,46] that individual "impact indicators" are also produced by non-impact processes, thus not requiring an impact. Finally, (8) another study[43] argued that YD climate change had little effect on people and their animal prey across the central portion of the US. For a limited number of selected contributions relevant to this hypothesis, see Supplementary Table A3 of Wolbach et al.[49].

## References


1. Hoffman, E. L. & Dunn, B. Sample preparation and bulk analytical methods for PGE. CIM 461 Special Volume 54: The Geology, Geochemistry and Mineral Beneficiation of Platinum Group 462 Elements. Edited by Louis J. Cabri, pp.1–11 (2002).
2. Davis, O. K. Spores of the dung fungus Sporormiella: increased abundance in historic sediments and before Pleistocene megafaunal extinction. *Quaternary Research* **28,** 290-294 (1987).
3. Davis, O. K. "Pollen analysis of Wildcat Lake, Whitman County, Washington: the introduction of grazing." PhD dissertation, Washington State University (1975).
4. Burney, D. A., G. S. Robinson, and L. P. Burney *Sporormiella* and the late Holocene extinctions in Madagascar. *Proceedings of the National Academy of Science* **100,** 10800-10805 (2003).
5. Burney, D. A., and T. F. Flannery Fifty Millennia of Catastrophic Extinctions after Human Contact. Trends in Ecology & Evolution **20,** 395–401 (2005).
6. Gill, J. L. Ecological Impacts of the Late Quaternary Megaherbivore Extinctions. *New Phytologist* **201,** 1163–1169 (2014).
7. Fiedel, S. J. The spore conundrum: Does a dung fungus decline signal humans' arrival in the Eastern United States? *Quaternary International.* doi:10.1016/j.quaint.2015.11.130 (2016).
8. Gill, J. L. *et al.* Pleistocene Megafaunal Collapse, Novel Plant Communities and Enhanced Fire Regimes in North America. *Science* **236,** 1100-1103 (2009).
9. Gill, J. L., John W. Williams, Stephen T. Jackson, Jeffrey P. Donnelly, and Grace C. Schellinger. "Climatic and Megaherbivory Controls on Late-Glacial Vegetation Dynamics: A New, High-Resolution, Multi-Proxy Record from Silver Lake, Ohio." *Quaternary Science Reviews* **34,** 66–80 (2012).
10. Robinson, G. S., L. Pigott Burney, and D. A. Burney. "Landscape Paleoecology and Megafaunal Extinction in Southeastern New York State." *Ecological Monographs* **75,** 295–315 (2005). https://doi.org/10.1890/03-4064

11. Robinson, G. S., and D. A. Burney. "The Hyde Park Mastodon and Palynological Clues to Megafaunal Extinction." In *Mastodon Paleobiology, Taphonomy, and Paleoenvironment in the Late Pleistocene of New York State: Studies on the Hyde Park, Chemung, and North Java Sites*, 291–299 (2008).
12. Davis, O. K., and D. S. Shafer Sporormiella fungal spores, a palynological means of detecting herbivore density. *Paleogeography, Paleoclimatology, Paleoecology* **237,** 40-50 (2006).
13. Baker, A..G. Cornelissen, P., Bhagwat, S.A., Vera, F.W.M., Willis, K.J., Quantification of population sizes of large herbivores and their long-term functional role in ecosystems using dung fungal spores. *Methods in Ecology and Evolution* **7,** 1273–1281. doi:10.1111/2041-210X.12580 (2016).
14. Gill, J. L. *et al.* Linking abundances of the dung fungus Sporormiella to the density of bison: implications for assessing grazing by megaherbivores in palaeorecords. *Journal of Ecology* **101**, 1125-1136 (2013).
15. Parker, N. E., and J. W. Williams, Influences of climate, cattle density, and lake morphology on *Sporormiella* abundances in modern lake sediments in the US Great Plains. *The Holocene* **22,** 475-483 (2011).
16. Raper, D. and M. Bush A test of Sporormiella representation as a predictor of megaherbivore presence and abundance. *Quaternary Research* **17,** 490-496 (2009).
17. Bell, A. Dung fungi: an illustrated guide to coprophilous fungi in New Zealand. Victoria University Press, Wellington, New Zealand (1983).
18. Perrotti, A. G., and E. van Asperen. "Dung fungi as a proxy for megaherbivores: opportunities and limitations for archaeological applications." *Vegetation History and Archaeobotany*: **1-12** (2018).
19. Johnson, C. N., S. Rule, S. G. Haberle, C.S.M. Turney, A. Peter Kershaw and B. W. Brook, Using dung fungi to interpret decline and extinction of megaherbivores: problems and solutions. *Quaternary Science Reviews* **110,** 107-113 (2015).
20. Watts, W. A. Late-Quaternary Vegetation History at White Pond on the Inner Coastal Plain of South Carolina. *Quaternary Research* **13,** 187–199 (1980).
21. Krause, T. R. *et al.* Late Quaternary vegetation, climate, and fire history of the Southeast Atlantic Coastal Plain based on a 30,000-yr multi-proxy record from White Pond, South Carolina, USA. *Quaternary Research* **91,** 861-880 (2018).
22. Halligan, J. J. *et al.* Pre-Clovis occupation 14,550 years ago at the Page-Ladson site, Florida, and the peopling of the Americas. *Science Advances* **2,** e1600375–e1600375. doi:10.1126/sciadv.1600375 (2016).
23. Perrotti, A. G., Pollen and *Sporormiella* evidence for terminal Pleistocene vegetation change and megafaunal extinction at Page-Ladson, Florida. *Quaternary International* **466,** 256-268 (2018).
24. Firestone, R. B. *et al.* Evidence for an extraterrestrial impact 12,900 years ago that contributed to the megafaunal extinctions and the Younger Dryas cooling. *Proc. Natl. Acad. Sci. U.S.A.***104,** 16016–16021 (2007).

25. Firestone R. B. *et al.* Analysis of the Younger Dryas impact layer. *J. of the Siberian Federal University, Eng Techn* **1,** 30–62 (2010).
26. Goderis, S., Tagle, R., Belza, J., Smit, J., Montanari, A., Vanhaecke, F., et al. 2013. Reevaluation of siderophile element abundances and ratios across the Cretaceous-Paleogene (K-Pg) boundary: implications for the nature of the projectile. Geochimica Et Cosmochimica Acta 120: 417–446.
27. Moore, C.R. and West, A. 2019. Pt, Pd data from GERM+other Papers--2c. https://www.researchgate.net/publication/333433185_Moore_et_al_2017--Pt_Pd_data_from_GERMother_Papers--2c. DOI: 10.13140/RG.2.2.18101.73447
28. Shapiro, B. and M. Hofreiter, A Paleogenomic Perspective on Evolution and Gene Function: New Insights from Ancient DNA. *Science* **343,** Issue 6169, 1236573 (2014).
29. Fulton, T. L. and Beth Shapiro, Setting Up an Ancient DNA Laboratory. *Methods Mol Biol.* **1963,** 1-13. doi: 10.1007/978-1-4939-9176-1 (2019).
30. Seersholm, F.V. *et al.* Subsistence practices, past biodiversity, and anthropogenic impacts revealed by New Zealand-wide ancient DNA survey. *Proc. Natl. Acad. Sci. USA* **115**, 7771–7776 (2018).
31.Gansauge, Marie-Theres *et al.* Single-stranded DNA library preparation from highly degraded DNA using T4 DNA ligase, *Nucleic Acids Research*, Volume **45,** e79 (2017).
32. Slon, V. *et al.* Mammalian mitochondrial capture, a tool for rapid screening of DNA preservation in faunal and undiagnostic remains, and its application to Middle Pleistocene specimens from Qesem Cave (Israel). *Quaternary International* **398,** 210-218 (2016).
33. Kirillova, I. V. *et al.* Discovery of the skull of Stephanorhinus kirchbergensis (Jäger, 1839) above the Arctic Circle. *Quaternary Research* **88,** 570 (2017).
34. Schmieder R. and Edwards R. Quality control and preprocessing of metagenomic datasets. *Bioinformatics* **27,** 863-864 (2011).
35. Li H. and R. Durbin Fast and accurate short read alignment with Burrows-Wheeler Transform. *Bioinformatics* **25,** 1754-1760 (2009).
36. Li H. *et al.* The Sequence alignment/map (SAM) format and SAMtools. *Bioinformatics* **25,** 2078-9 (2009).
37. Kennett, J. P. *et al*. Bayesian chronological analyses consistent with synchronous age of 12,835–12,735 Cal BP for Younger Dryas boundary on four continents. *Proc. Natl. Acad. Sci. U.S.A.***112,** E4344-E4353 (2015).
38. Hanlin, R. T. Illustrated Genera of Ascomycetes, Volumes I & II. , Book, Whole. St. Paul, Minnesota: The American Phytopatholocial Society (1990).
39. Mighall, T. M,, A. Martínez Cortizas, H. Biester, and S. E. Turner Proxy Climate and Vegetation Changes during the Last Five Millennia in NW Iberia: Pollen and Non-Pollen Palynomorph Data from Two Ombrotrophic Peat Bogs in the North Western Iberian Peninsula. *Review of Palaeobotany and Palynology* **141,** 203–223 (2006).
40. Ahmed, S. I., and R.F. Cain Revision of the Genera Sporormia and Sporormiella. Canadian *Journal of Botany* **50,** 419–477 (1972).
41. Pino, M. *et al.* Northwestern Chilean Patagonian sediment record supports YDB cosmic impact triggering of biomass burning, climate change, and megafaunal extinctions (~12.8 ka). *Scientific Reports* **9**, Article number: 4413 (2019).
42. Boslough, M. et al. In *Arguments and evidence against a Younger Dryas impact event in*

*Climates, landscapes, civilizations*, Geophysical Monograph Series Vol. 198 (eds Giosan, L. et al.) 13–26 (Am Geophys Union, 2013).
43. Meltzer, D. J., Holliday, V. T., Cannon, M. D. & Miller, D. S. Chronological evidence fails to support claim of an isochronous widespread layer of cosmic impact indicators dated to 12,800 years ago. *Proc Nat Acad Sci* **111**, E2162–E2171 (2014).
44. Holliday, V., Surovell, T. & Johnson, E. A blind test of the Younger Dryas impact hypothesis. *PloS One* **11**, e0155470 (2016).
45. Meltzer, D. J. & Holliday, V. T. Would North American Paleoindians have noticed younger Dryas age climate changes? *J World Prehist* **23**, 1–41 (2010).
46. Stuart, A. J. Late Quaternary megafaunal extinctions on the continents: a short review. *Geol J* **50**, 338–363 (2015).
47. Holliday, V. T., Surovell, T., Meltzer, D. J., Grayson, D. K. & Boslough, M. The Younger Dryas impact hypothesis: a cosmic catastrophe. *J Quat Sci* **29**, 515–530 (2014).
48. Marlon, J. R. *et al.* Wildfre responses to abrupt climate change in North America. *Proc Nat Acad Sci* **106**, 2519–2524 (2009).
49. Wolbach, W. S. *et al.* Extraordinary biomass-burning episode and impact winter triggered by the Younger Dryas cosmic impact <12,800 years ago. 2. Lake, marine, and terrestrial sediments. *J Geol* **126,** 185–205 (2018b).

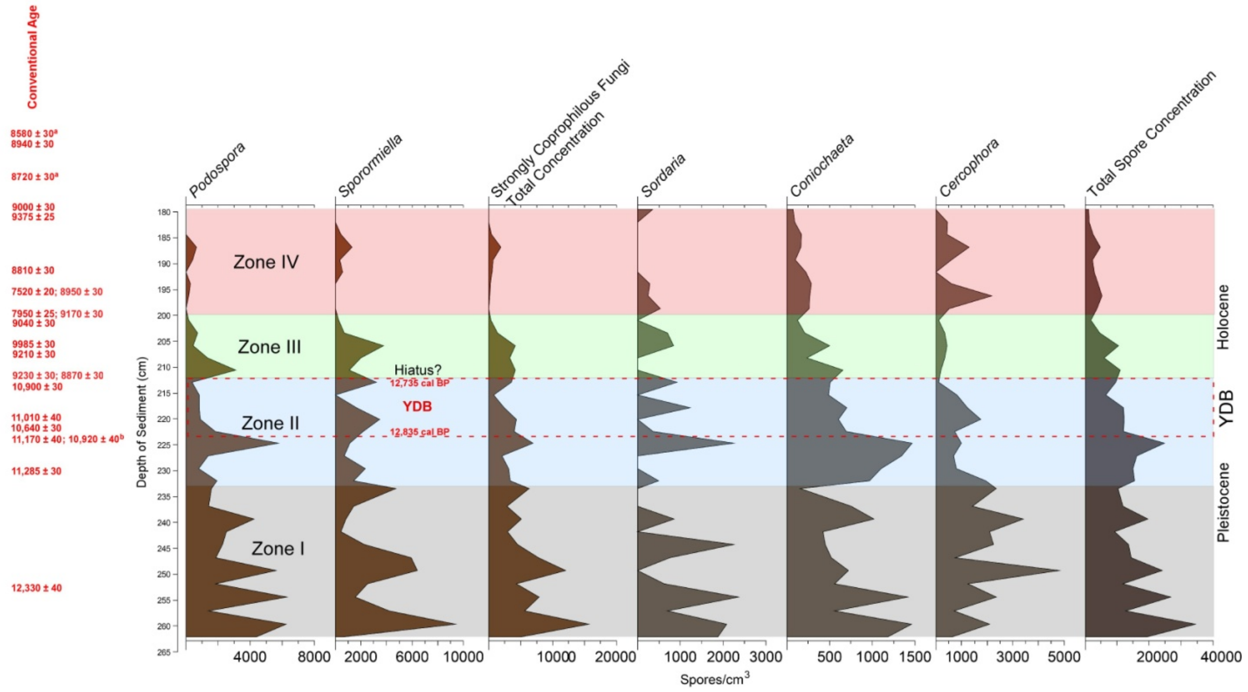


**Supplementary Figure 1**. Spore concentrations (spores/cm$^3$) plotted by depth and spore zone. Brown graphs represent taxa that strongly prefer dung as substrate. Gray graphs represent fungi that can be found on dung, but are not strongly coprophilous. Conventional radiocarbon dates on are shown on the left for 2016 cores (n=22). The Bayesian modeled age range for the YD onset (12,835–12,735 cal yr BP at 95% Confidence Interval) based on Kennett *et al.*[35] is shown as red dashed line. Note the x-axis scale variability.

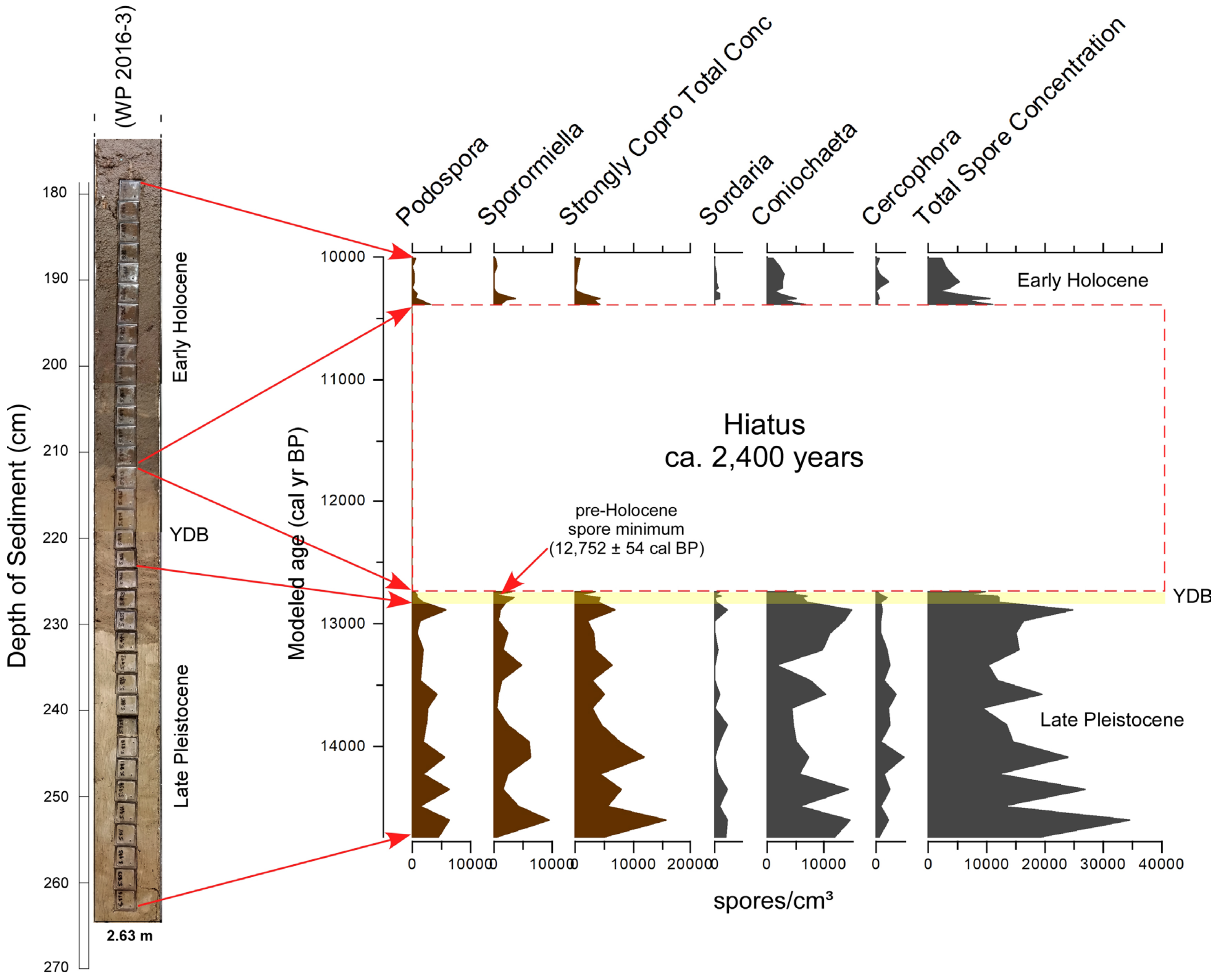


**Supplementary Figure 2.** Spore concentrations (spores/cm$^3$) plotted by modeled age BP shown relative to spore samples locations from core 2016-3. Brown graphs (3 on left) represent taxa that strongly prefer dung as substrate. Gray graphs (4 on right) represent fungi that can be found on dung, but are not strongly coprophilous. A ca. 2,400 year hiatus occurs in the core in a 1-cm interval (211 to 212 cm in core 2016-3). This hiatus occurs between lithologic units IIb and IIIa (see Supplementary Figure 8). Increasing spore counts (spores/cm$^3$) at the top of the modeled YDB, and after the pre-Holocene spore minimum, are likely due to inclusion of Early Holocene spores present in the core during or post-hiatus. This is because two spore samples (samples 14 and 15; see Fig. 2 in main paper) overlap the modeled hiatus between 211-212 cm in Core 2016-3.

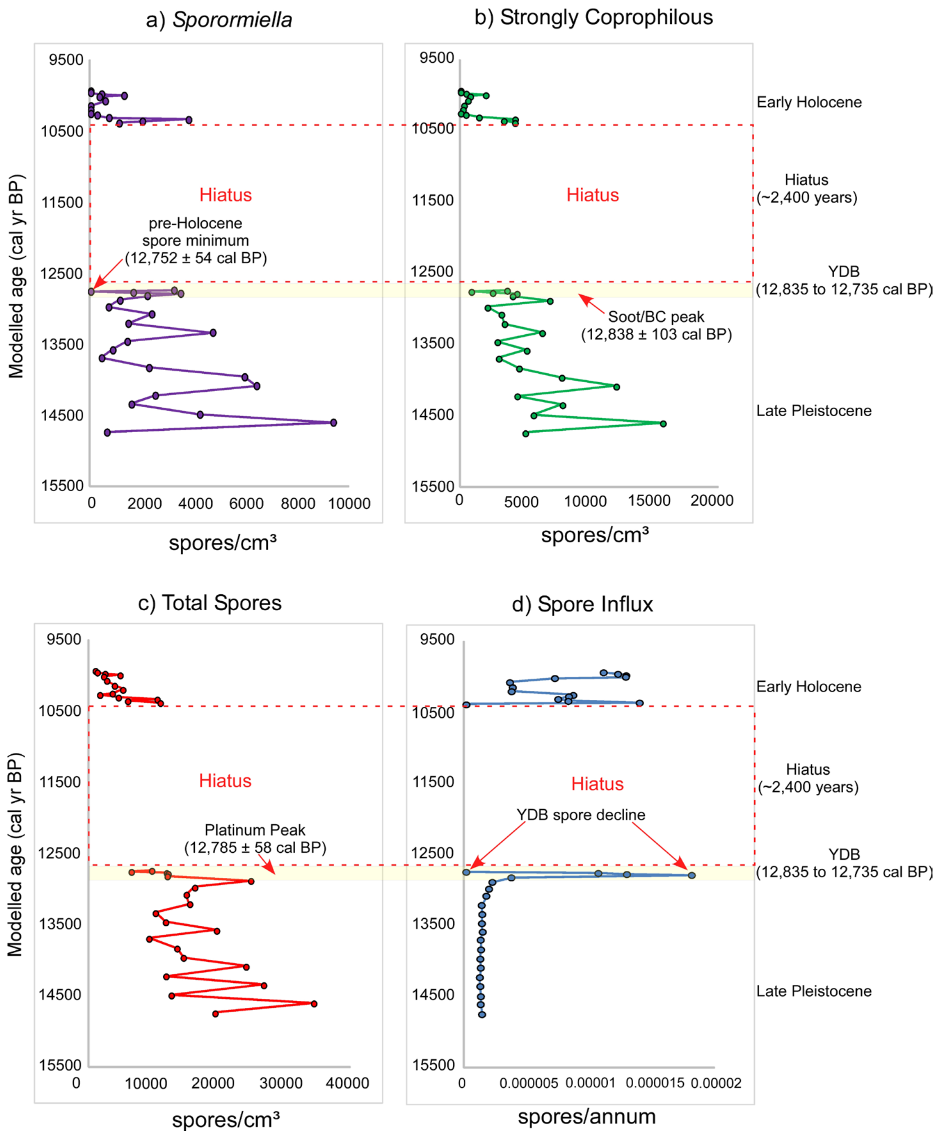


**Supplementary Figure 3.** Spore concentrations (spores/cm$^3$) by modeled year for a) *Sporormiella*, b) strongly coprophilous, and c) total spores. Also shown is d) spore influx (spores/annum) plotted by modeled age BP. Note ca. 2,400 year hiatus above the YDB. The Bayesian modeled age range for the YD onset (12,835–12,735 cal yr BP at 95% Confidence Interval) based on Kennett *et al.*[35] is shown as light-yellow zone. All impact proxies (soot/BC and Pt) are confined to the YDB and temporally constrained to the YD onset by Bayesian statistical analysis of 22 AMS dates (see Fig. 2 and Supplementary Table 2). The pre-Holocene spore minimum (12,752 +- 54 cal BP) occurs below the temporal hiatus and represents a dramatic decline in overall spore abundance during the YD onset as indicated by concentration data (spores/cm$^3$) and influx calculated as spores/annum based on the Bayesian age/depth model. The two very low spore influx estimates immediately above and below the hiatus are likely artificially low because spore concentration (spores/annum) for these samples is minimized due to the extremely slow sedimentation rate predicted by the age-depth model.

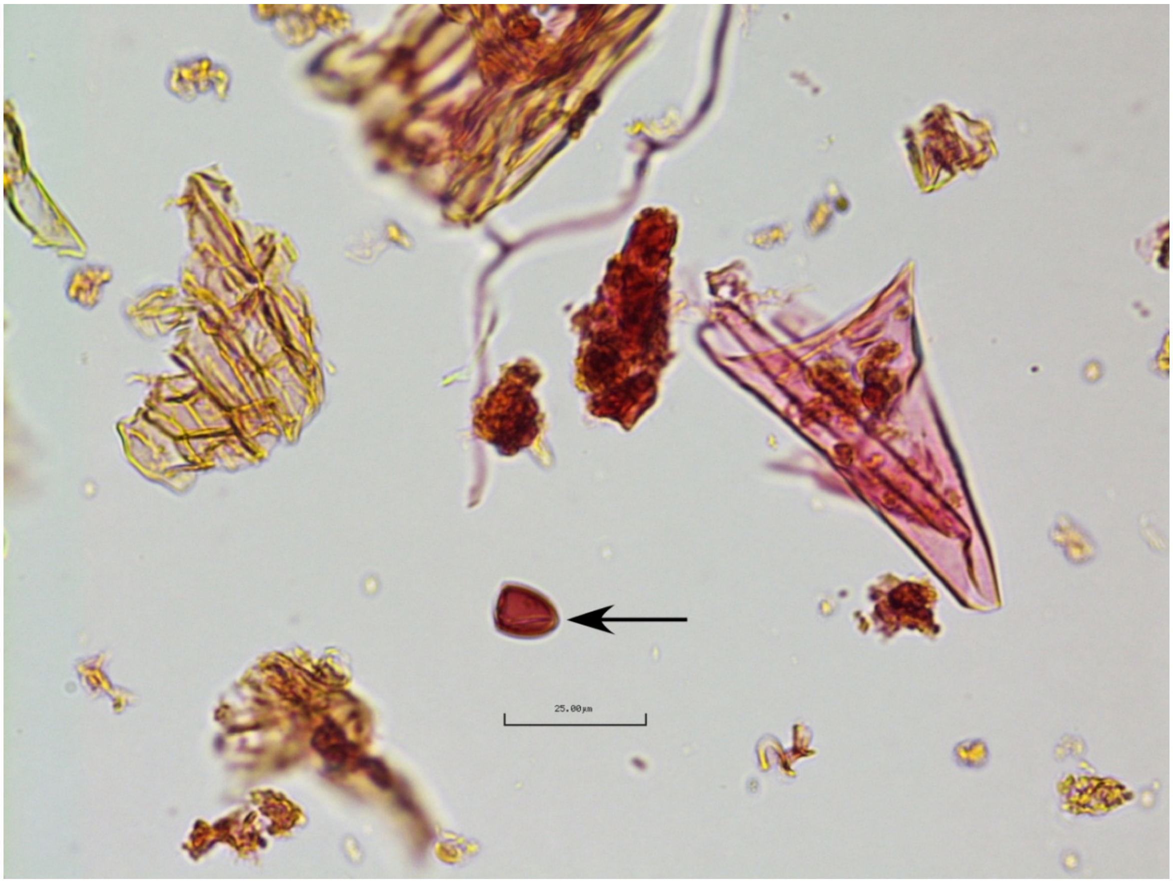


**Supplementary Figure 4.** Photomicrograph showing *Sporormiella* at White Pond (WP2016-3).

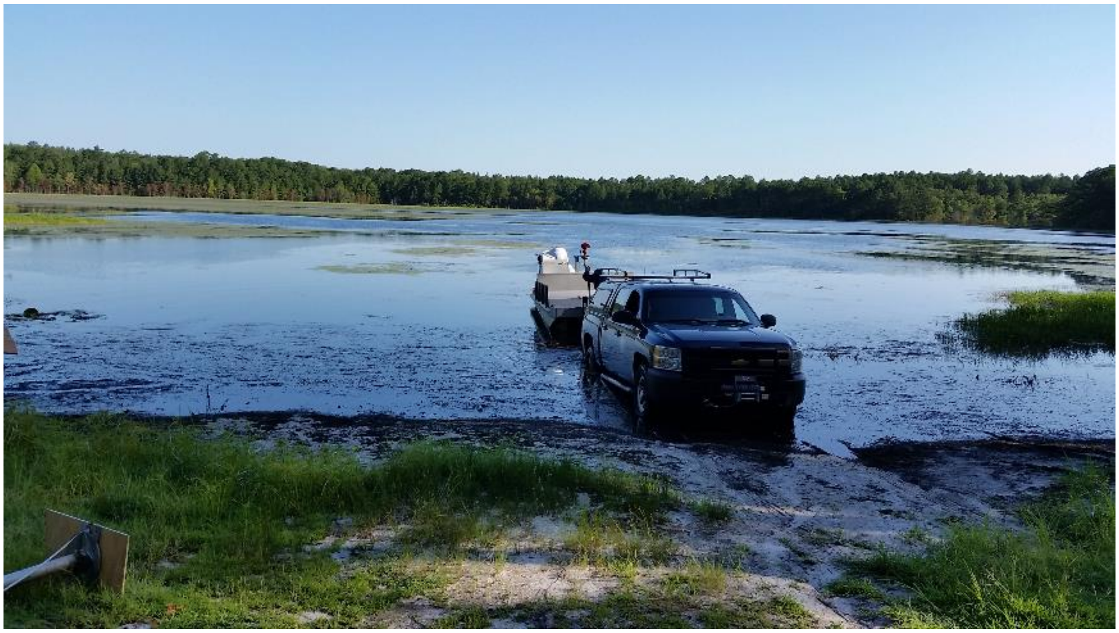

**Supplementary Figure 5.** Preparing to collect vibracores at White Pond.

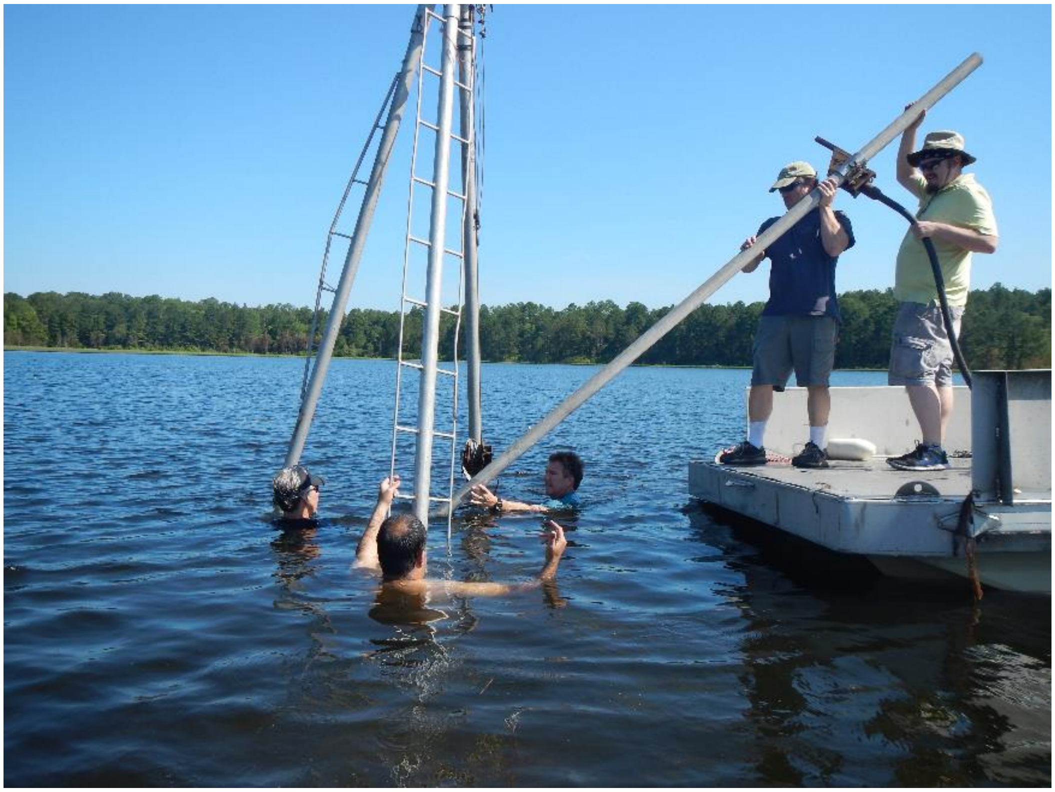

**Supplementary Figure 6.** Collecting one of four duplicate vibracores from White Pond in 2016.

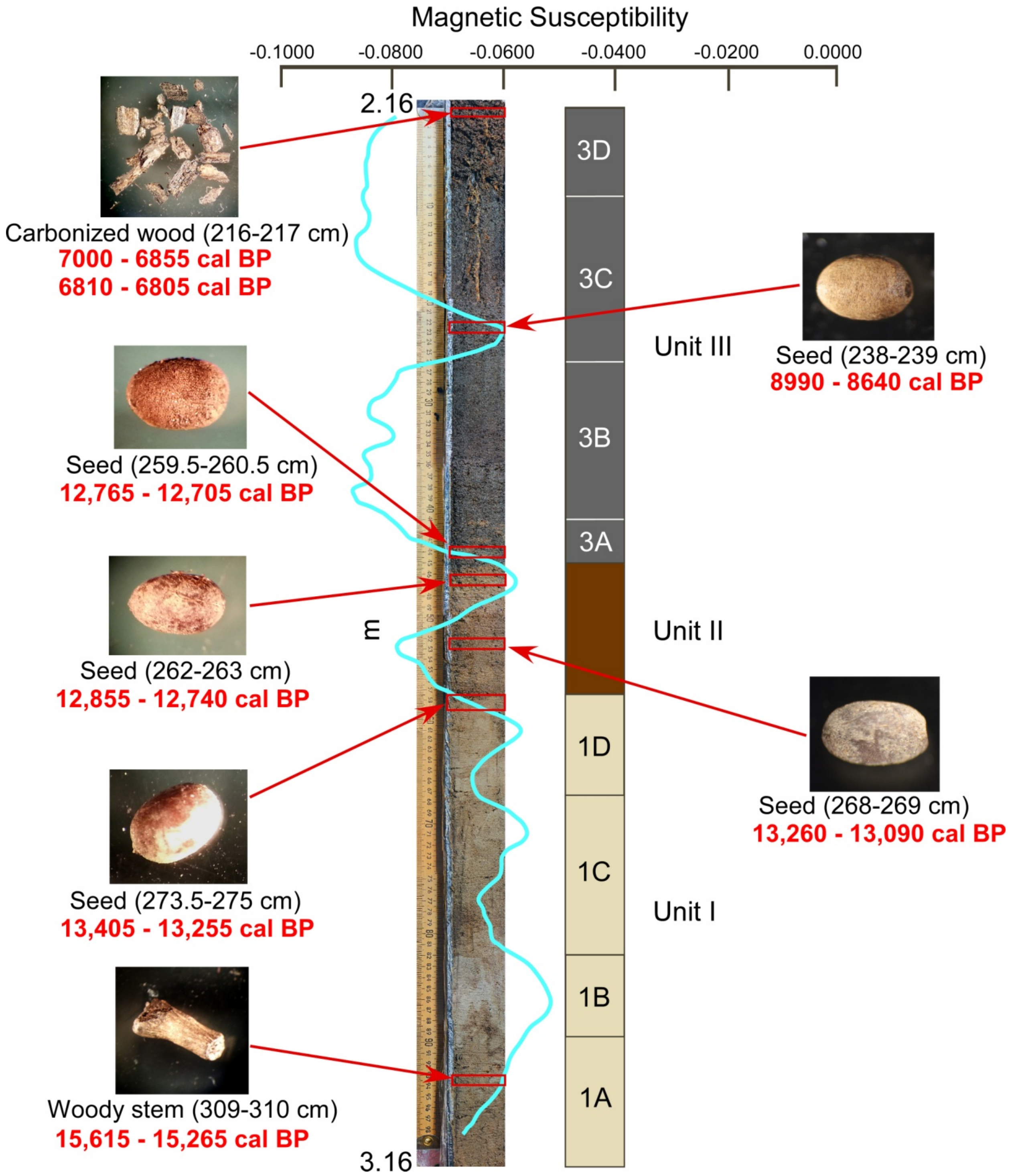


**Supplementary Figure 7.** Core collected in 2015 from ~2.16 to 3.16 meters below the sediment/water interface, showing core lithology, mass specific magnetic susceptibility, calibrated AMS dates (2-sigma range based on INTCAL13), and samples used for dating (see Supplementary Table 1).

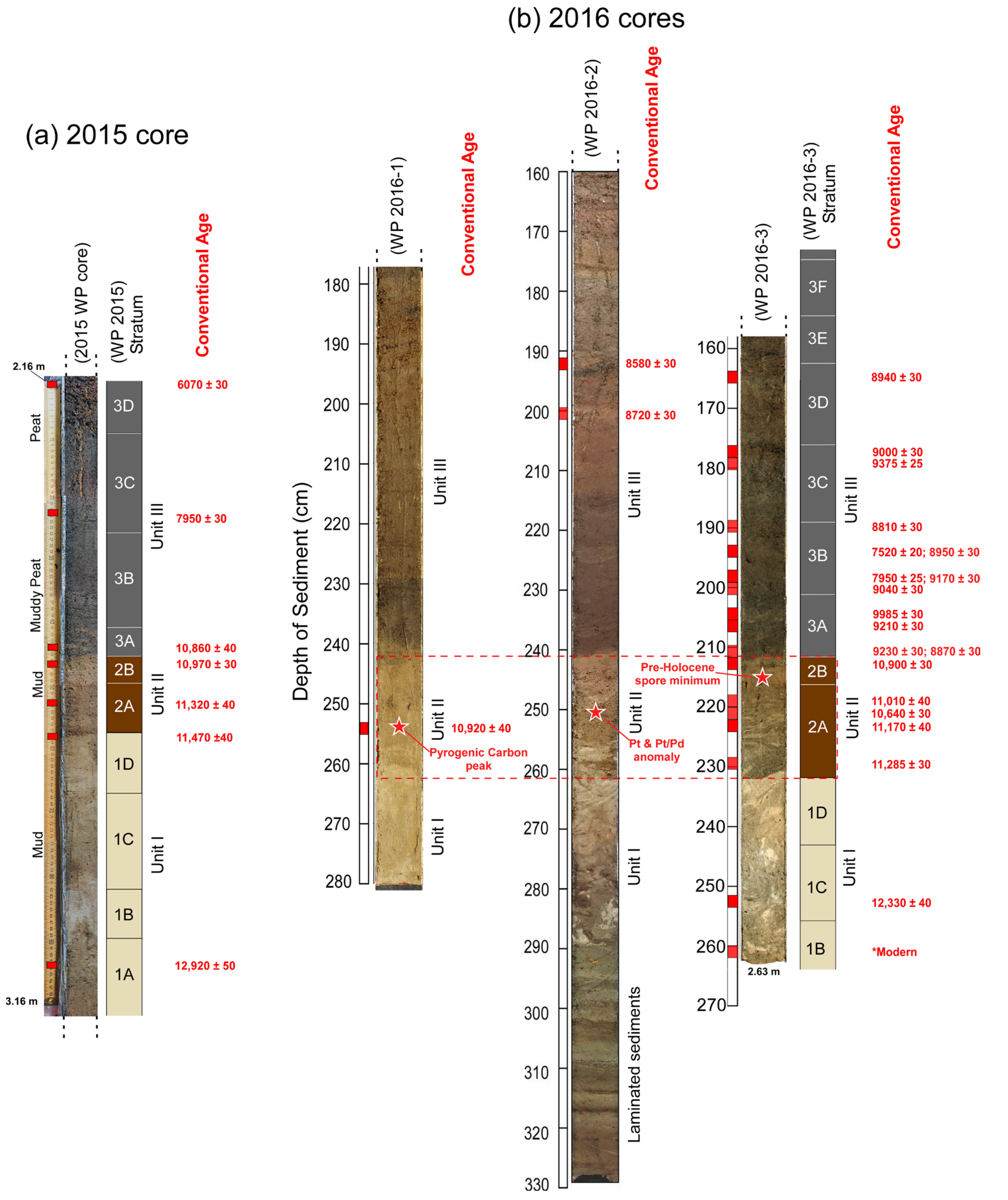


**Supplementary Figure 8.** Core sections with depth of sediment (cm), lithology, location of radiocarbon samples, and conventional radiocarbon dates ($^{14}C$/BP) for 2015 and 2016 cores (see Supplementary Table 1). The stratigraphic position of the Pt and Pt/Pd anomaly, pyrogenic carbon peak, and pre-Holocene spore minimum are shown for each 2016 core.

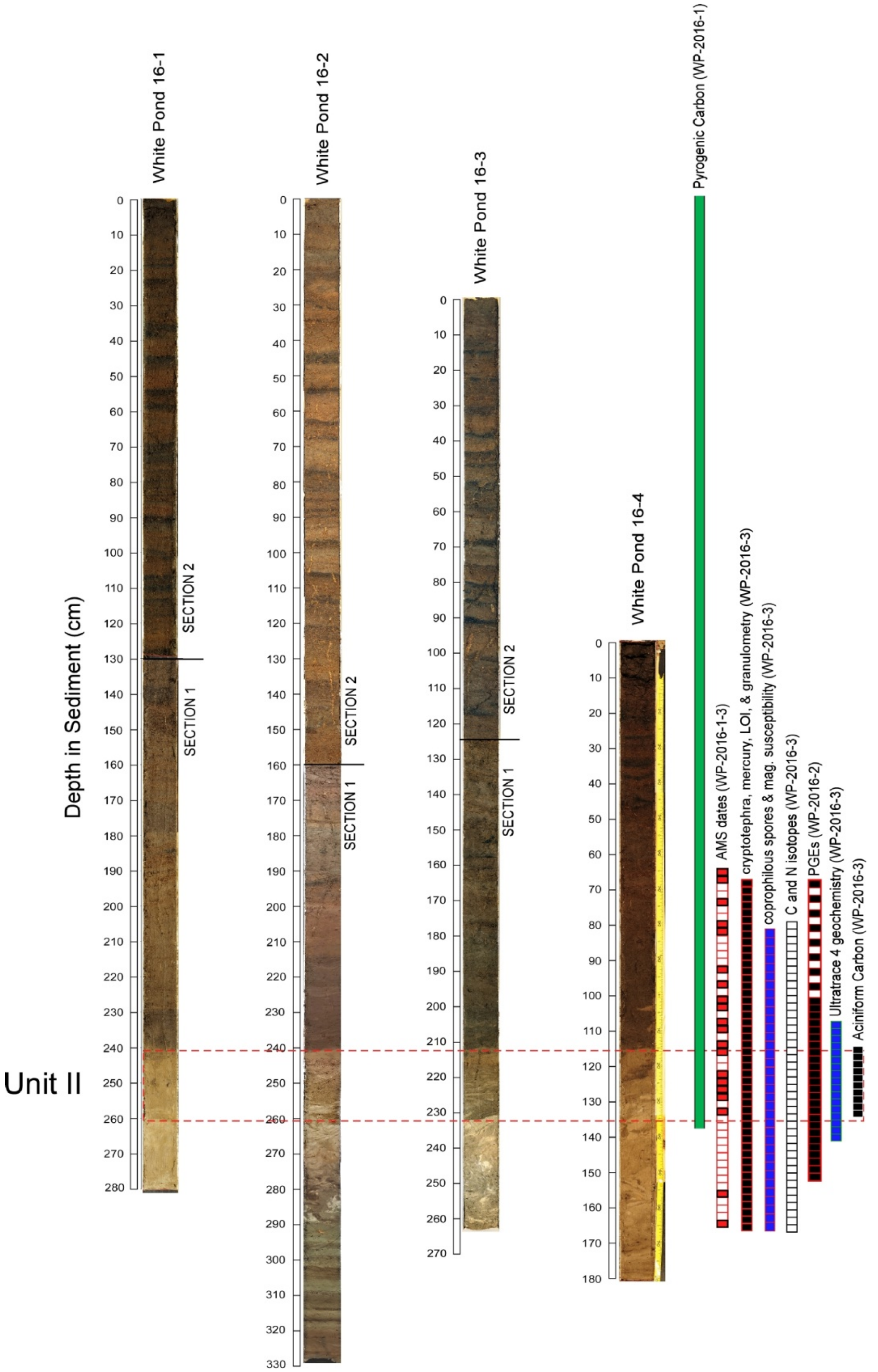


**Supplementary Figure 9.** Core photo mosaics for 2016 vibracores aligned using the mud to peat transition unit as a common core datum (top of Unit II). Analyses performed and range of tested samples are shown on the right for respective duplicate cores (2016-1, 2, 3, and 4).

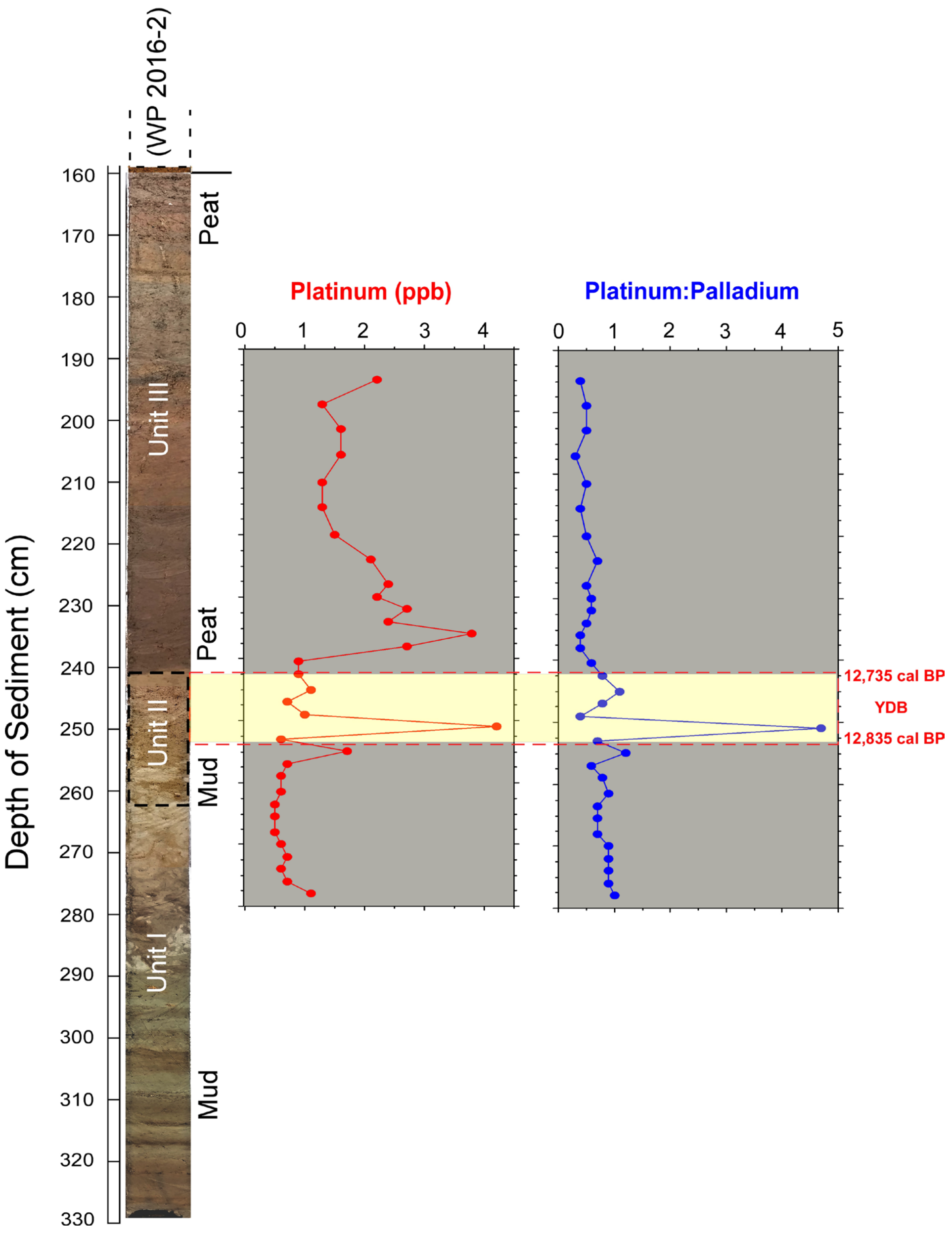


**Supplementary Figure 10.** Platinum (Pt) abundance (error = +/-0.1 ppb), the ratio of platinum to palladium (Pt/Pd) shown as data points (n=33) for 2-cm interval samples (core 2016-2). See Supplemental Table 3. The Bayesian modeled age range for the YD onset (12,835–12,735 cal yr BP at 95% Confidence Interval) based on Kennett *et al.*[35] is shown as light-yellow zone within stratigraphic Unit II.

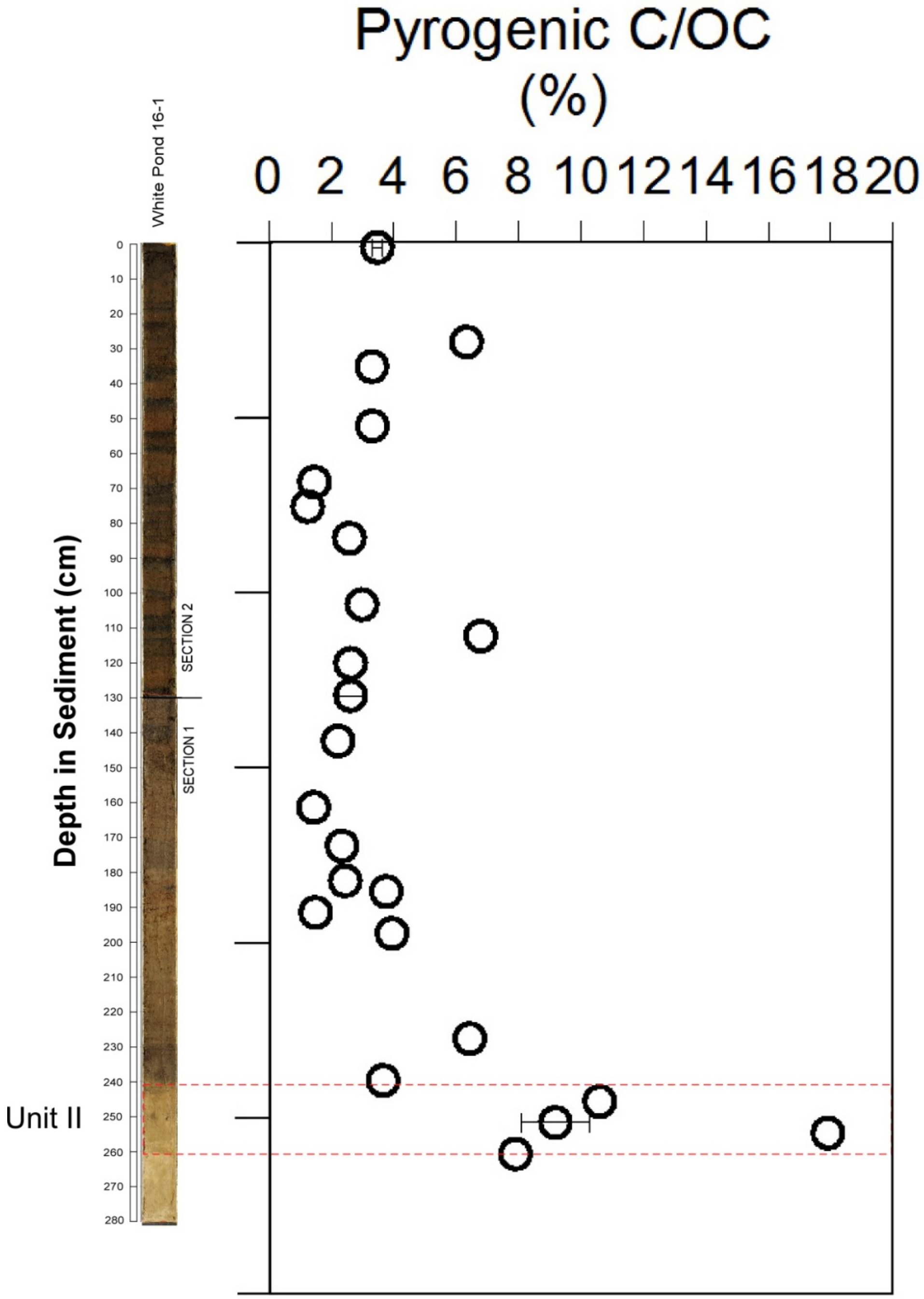


**Supplementary Figure 11.** Pyrogenic carbon analysis [Soot C/OC (%)] for White Pond vibracore 2016-1 (see Supplementary Table 8).

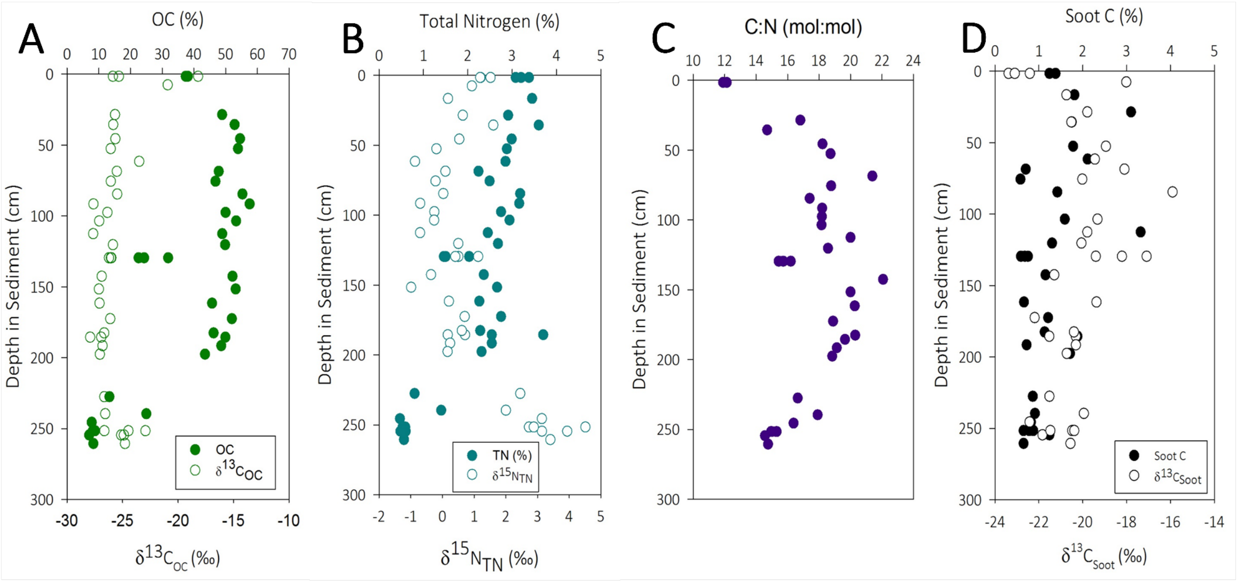


**Supplementary Figure 12.** A) Organic carbon concentration (top panel) & stable isotope ratio (bottom panel); B) Total nitrogen concentration (top panel) & stable isotope ratio (bottom panel); C) Carbon to nitrogen ratio; D) Soot carbon concentration (top panel) & stable isotope ratio (bottom panel). All data was collected from core 2016-1. Note: Panel D depicts the raw values of soot carbon, not normalized to OC, which is shown in Supplementary Fig 12, panel A. Figure 2 and Supplementary Fig. 11 show soot carbon normalized to OC.

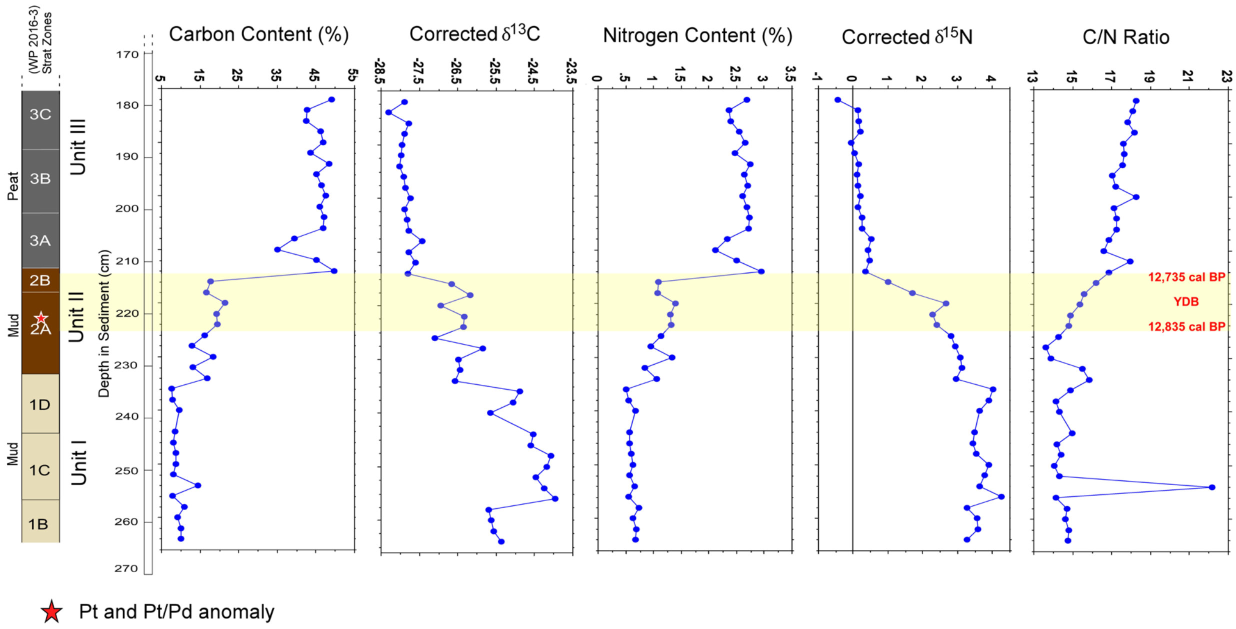


**Supplementary Figure 13.** Carbon and Nitrogen stable isotope analysis for bulk sediments from vibracore 2016-3 (see Supplementary Table 9). The Bayesian modeled age range for the YD onset (12,835–12,735 cal yr BP at 95% Confidence Interval) based on Kennett *et al.*[35] is shown as light-yellow zone within stratigraphic Unit II. The stratigraphic position of the Pt and Pt/Pd anomaly is also shown.

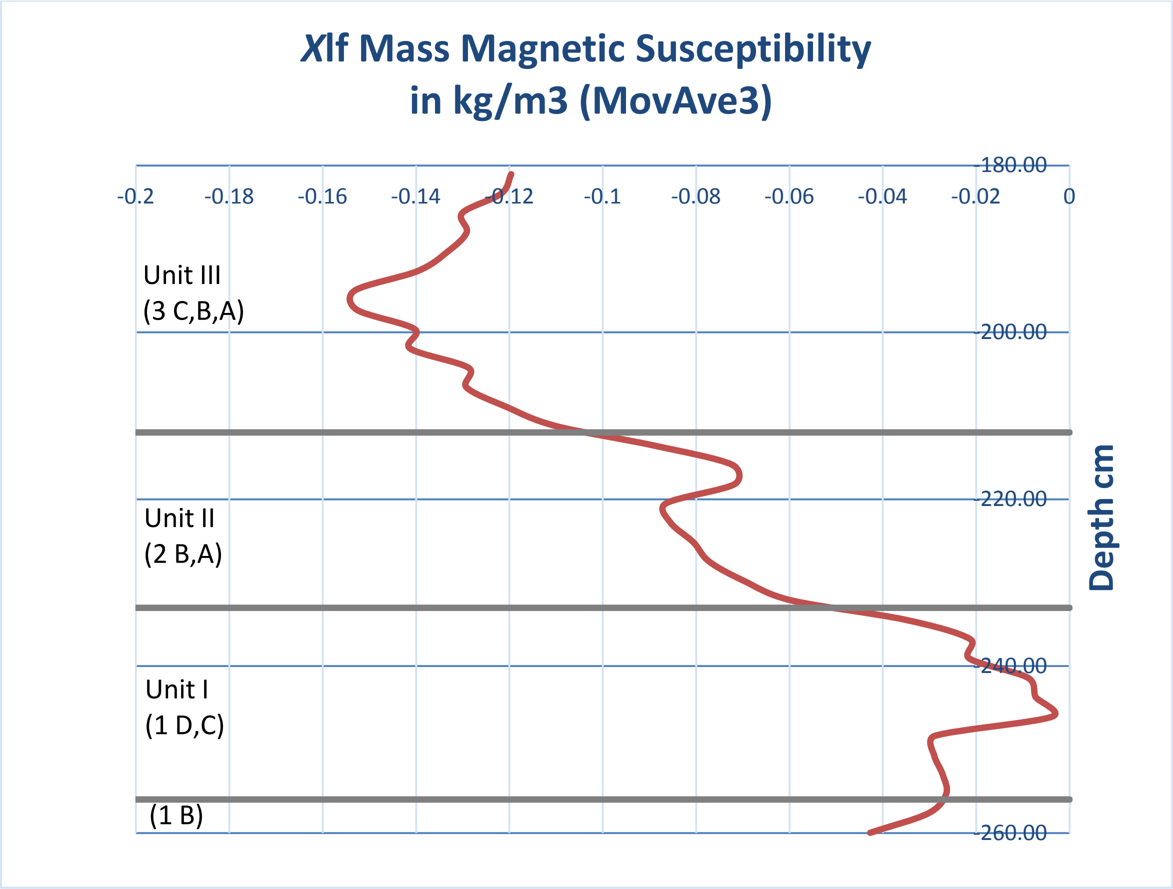


**Supplementary Figure 14.** Mass Specific Magnetic Susceptibility with stratigraphic units.

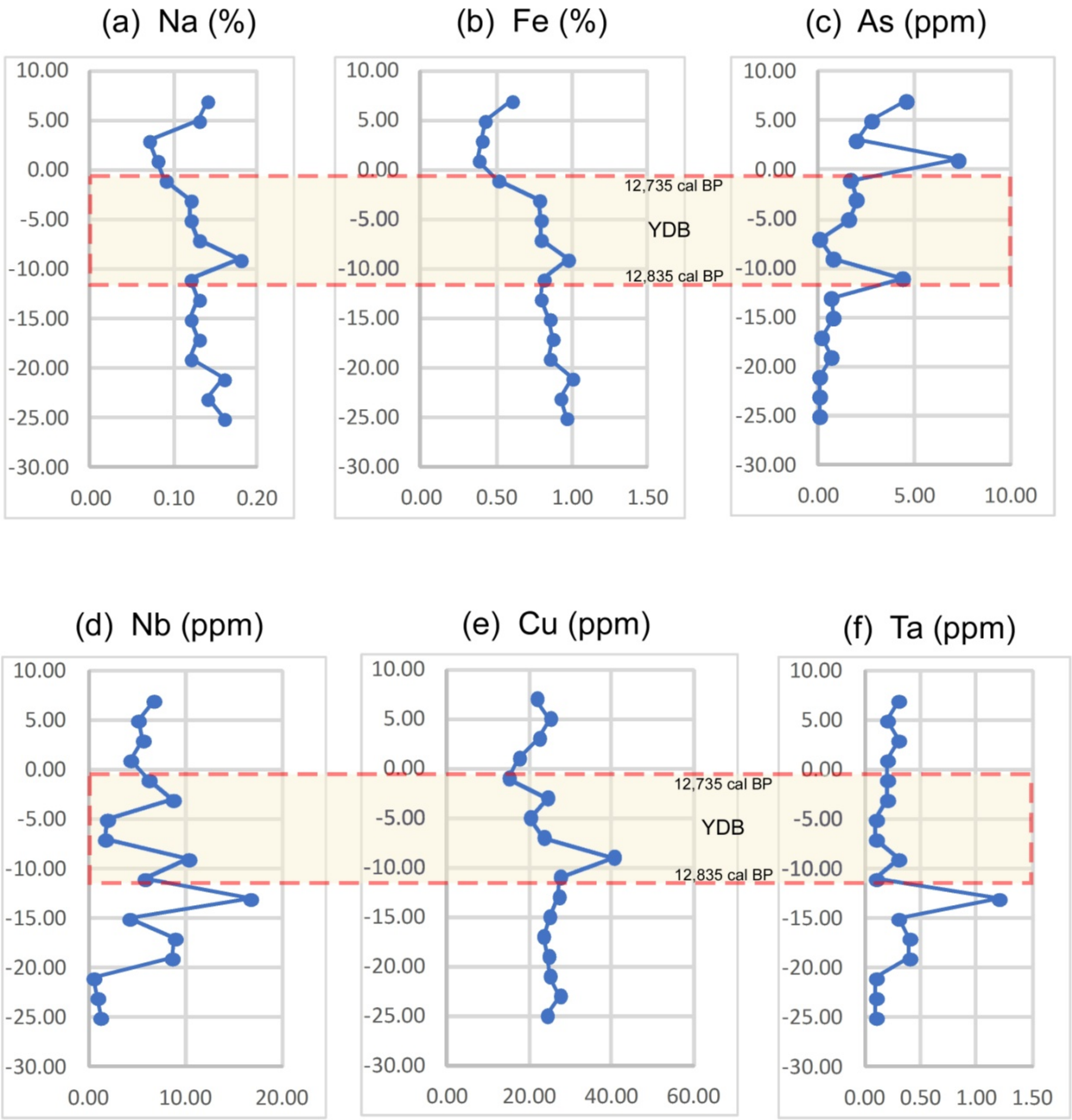


**Supplementary Figure 15.** Graphs (% and ppm) for select elements (a) Na [sodium]; (b) Fe (iron); (c) As [arsenic]; (d) Nb [niobium]; (e) Cu [copper], and (f) Ta [tantalum] analyzed from core samples collected from WP2016-3 and analyzed using the Ultratrace 4 technique by Actlabs, Inc. (see Supplementary Figure 9 for location of samples and Supplementary Table 10 for data). Bayesian modeled age range for the YD onset (12,835–12,735 cal yr BP at 95% Confidence Interval) based on Kennett *et al.*[35] is shown as light-yellow zone.

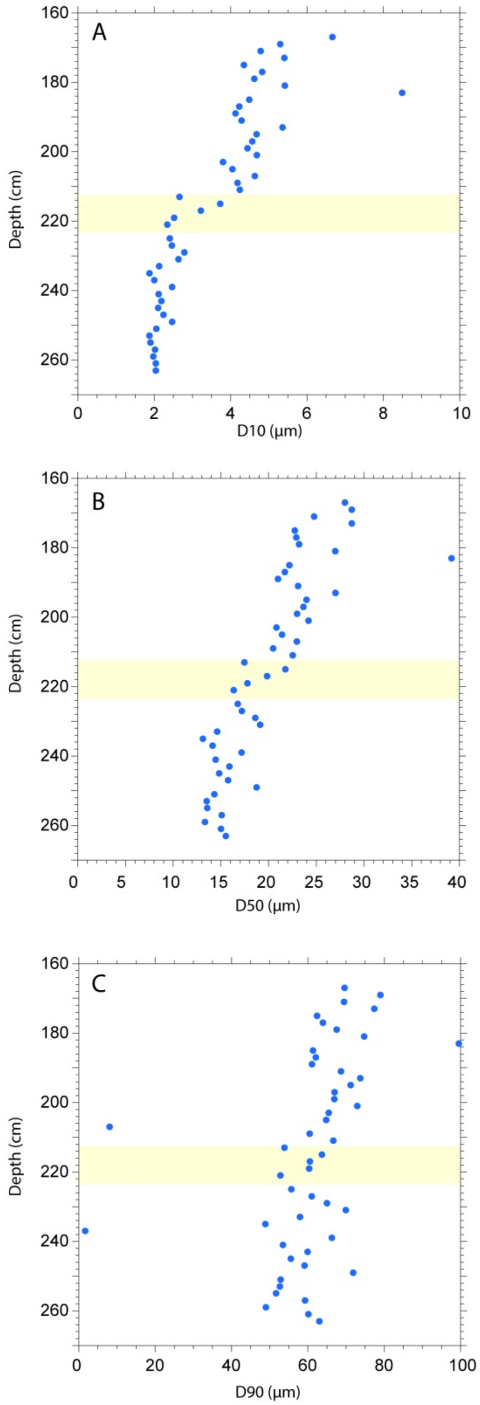


**Supplementary Figure 16.** Grain size distributions in core WP 2016-3. Down-core variations in D10, D50, and D90 are displayed in A, B, and C, respectively. The yellow shaded bar represents the modeld YD interval.

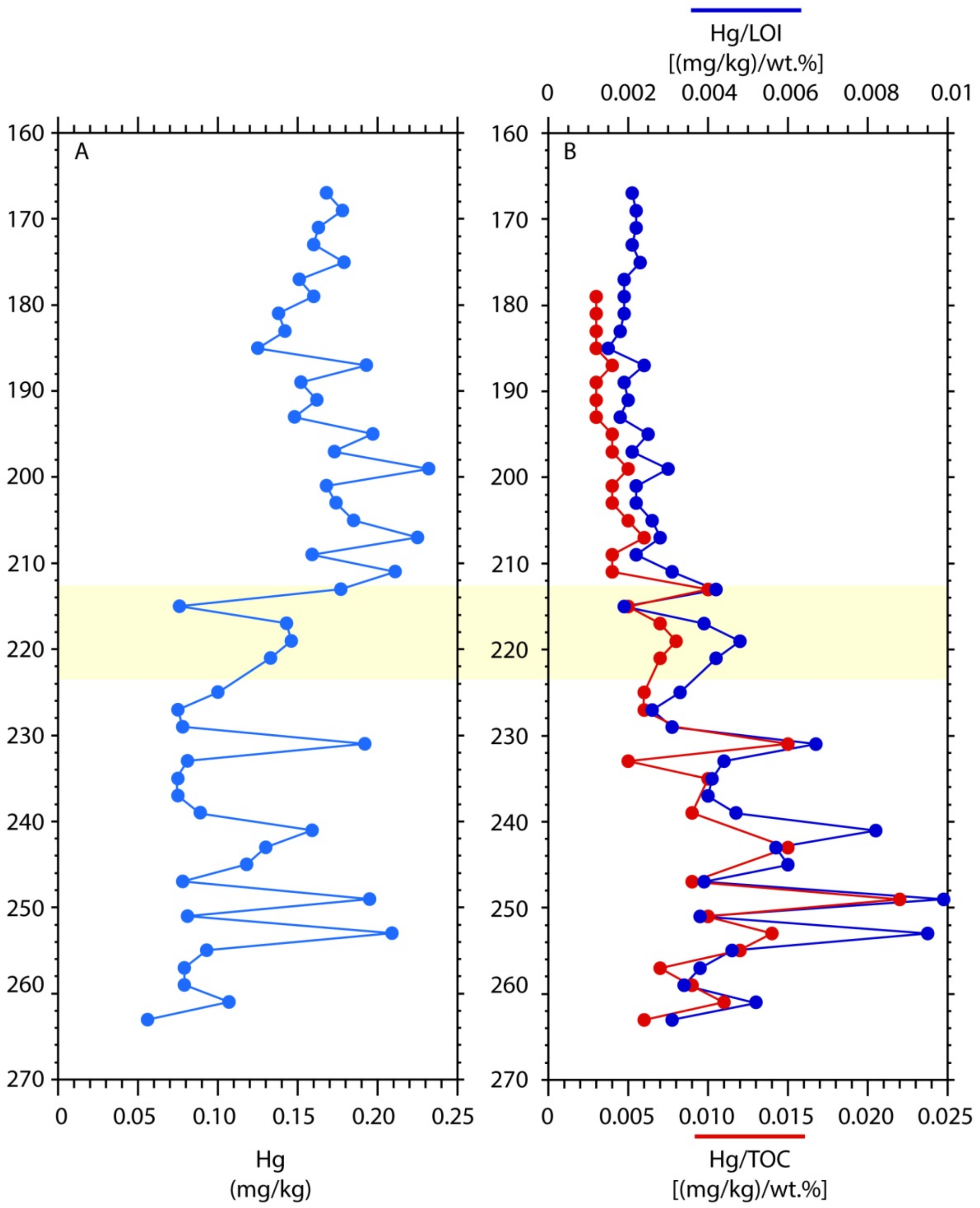


**Supplementary Figure 17.** Sedimentary Hg data in core WP 2016-3. (A) Raw Hg contents during the interval of interest. (B) Hg/LOI and Hg/TOC values during the interval of interest. Note that the increases in [Hg] beginning ~223 cm are muted when normalized to LOI and TOC. The yellow shaded bar represents the modeled YD interval.

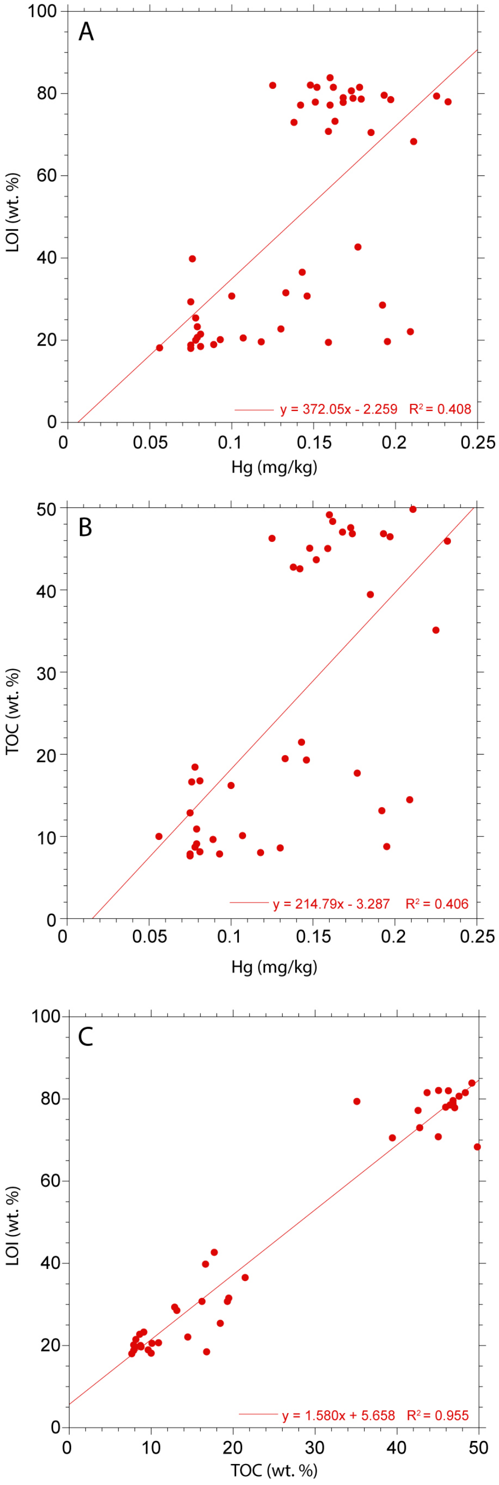


**Supplementary Figure 18.** Mercury, LOI, and TOC data in core WP 2016-3. (A) Loss-on-ignition is compared to [Hg] and suggests some control of LOI on [Hg]. (B) Total organic carbon contents are compared to [Hg] and suggest some control of TOC on [Hg]. (C) Loss-on-ignition is compared to TOC to show the strong relationship ($R^2$ = 0.95) between these two variables.

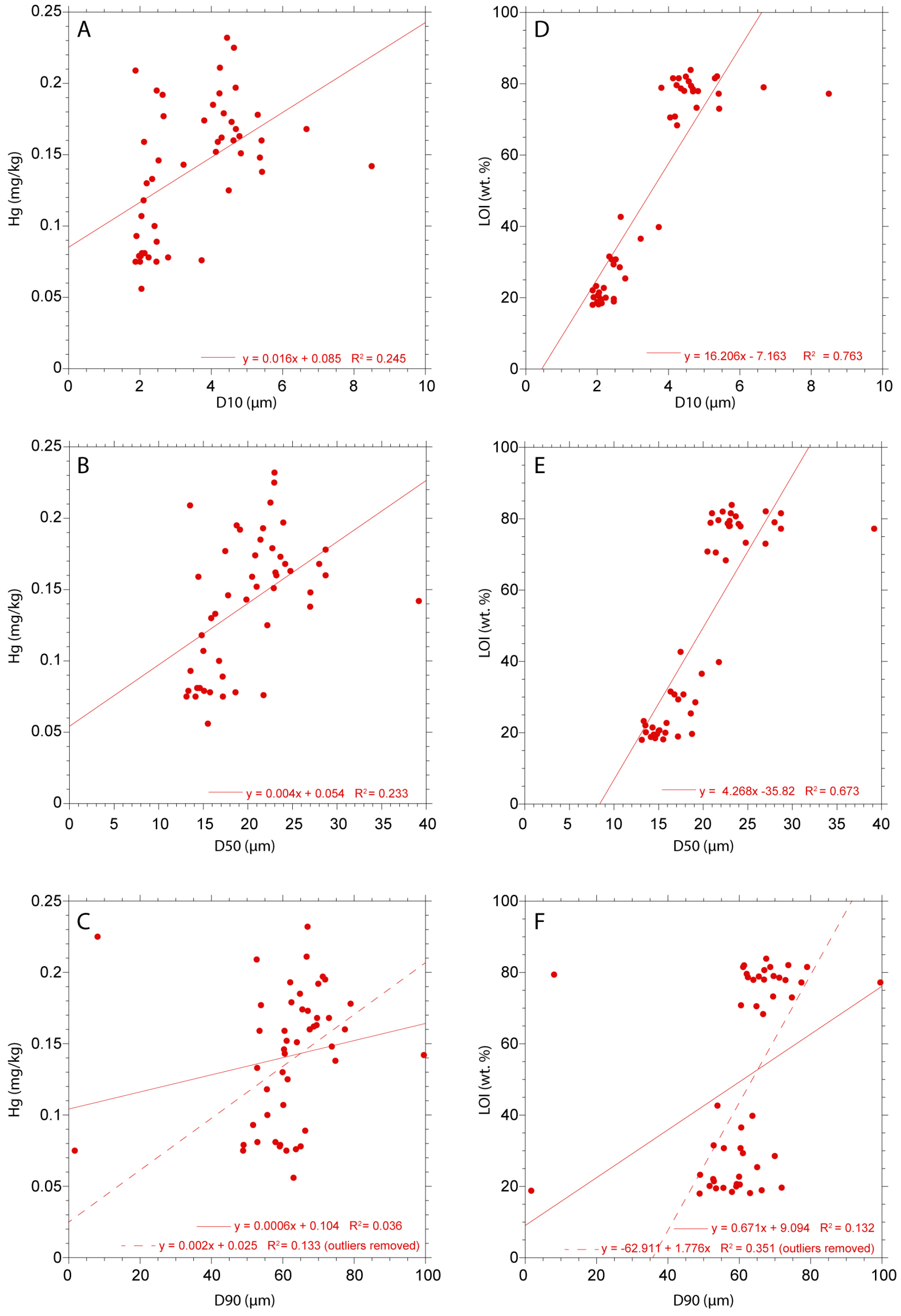


**Supplementary Figure 19.** Mercury content and LOI values compared to grain size in core WP 2016-3. (A, B, C) Raw Hg contents have a weak but significant correlation to D10, D50, and D90. (D, E, F) Loss-on-ignition values have a strong correlation to D10, D50, and D90, and have two major populations of data, which are consistent with the observed variations in lithology.

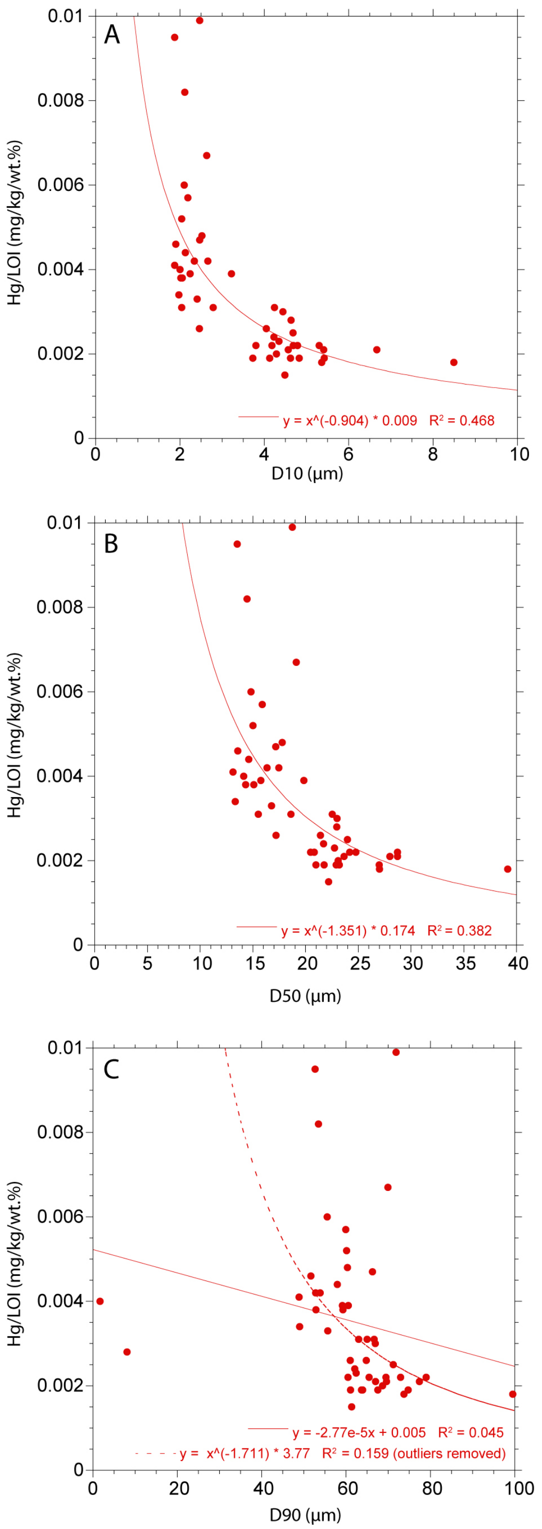


**Supplementary Figure 20.** Mercury/LOI values compared to grain size in core WP 2016-3. (A, B) Note the strong correlation between Hg/LOI with D10 and D50. (C) The relationship disappears in the D90 fraction.

Supplementary Table 1. Radiocarbon (AMS) dates for 2015 and 2016 cores from White Pond.

| Depth (cm)[a] | Sample interval[b] | Core[c] | Uncalibrated $^{14}$C age ($^{14}$C yr BP) | Calibrated age (2 sigma range)[d] | Material dated[e] | Lab number[f] |
|---|---|---|---|---|---|---|
| 162 | +46 to +48 | WP-2016-2 | 8580 +/- 30 | 9583 to 9495 | seed | Beta-470437 |
| 164 | +44 to +46 | WP-2016-3 | 8940 +/- 30 | 10,200 to 9919 | Alkali Insoluble Organics | Beta-499322 |
| 165 | +43.3 to +42.3 | WP-2015 | 6070 +/- 30 | 7000 to 6805 | charcoal | Beta-412233 |
| 170 | +38 to +40 | WP-2016-2 | 8720 +/- 30 | 9882 to 9552 | seed | Beta-470436 |
| 177 | +32 to +34 | WP-2016-3 | 9000 +/- 30 | 10,236 to 9975 | seed | Beta-469117 |
| 179 | +30 to +32 | WP-2016-3 | 9375 +/- 25 | 10,680 to 10,520 | seed | UCI-190027 |
| 188 | +21.8 to +22.8 | WP-2015 | 7950 +/- 30 | 8990 to 8640 | seed | Beta-430153 |
| 189.5 | +20 to +22 | WP-2016-3 | 8810 +/- 30 | 10,120 to 9694 | seed | Beta-470900 |
| 193.5 | +16 to +18 | WP-2016-3 | 7520 +/- 20 | 8388 to 8326 | seed | UCI-190028 |
| 193.5 (dup) | +16 to +18 (dup) | WP-2016-3 | 8950 +/- 30 | 10,208 to 9924 | Alkali Insoluble Organics | Beta-480583 |
| 198 | +12 to +14 | WP-2016-3 | 7950 +/- 25 | 8980 to 8649 | seed | UCI-190029 |
| 198 (dup) | +12 to +14 (dup) | WP-2016-3 | 9170 +/- 30 | 10,416 to 10,241 | Alkali Insoluble Organics | Beta-480582 |
| 200.2 | +10 to +12 | WP-2016-3 | 9040 +/- 30 | 10,241 to 10,186 | Alkali Insoluble Organics | Beta-502745 |
| 204 | +6 to +8 | WP-2016-3 | 9985 +/- 30 | 11,610 to 11,275 | seed | UCI-190030 |
| 206.4 | +4 to +6 | WP-2016-3 | 9210 +/- 30 | 10,490 to 10,257 | Alkali Insoluble Organics | Beta-502744 |
| 210 | +1.1 to +2.1 | WP-2015 | 10,860 +/- 40 | 12,765 to 12,705 | seed | Beta-415415 |
| 210.5 | 0 to +2 | WP-2016-3 | 8870 +/- 30 | 10,169 to 9887 | seed | Beta-470901 |
| 210.5 (dup) | 0 to +2 (dup) | WP-2016-3 | 9230 +/- 30 | 10,500 to 10,276 | Alkali Insoluble Organics | Beta-499326 |
| 212.5 | 0.6 to -1.6 | WP-2015 | 10,970 +/- 30 | 12,855 to 12,740 | seed | Beta-414623 |
| 212.5 | 0 to -2 | WP-2016-3 | 10,900 +/- 30 | 12,811 to 12,705 | seed | Beta-450657 |
| 219 | -6 to -8 | WP-2016-3 | 11,010 +/- 40 | 13,001 to 12,742 | seed | Beta-453269 |
| 219.5 | -6.8 to -7.8 | WP-2015 | 11,320 +/- 40 | 13,260 to 13,090 | seed | Beta-430154 |
| 221 | -8 to -10 | WP-2016-3 | 10,640 +/- 30 | 12,694 to 12,650 | plant macrofossils | Beta-450658 |
| 223.5 | -12 to -14 | WP-2016-1 | 10,920 +/- 40 BP | 12,859 to 12,701 | seed | Beta-502827 |
| 223 | -10 to -12 | WP-2016-3 | 11,170 +/- 40 | 13,126 to 12,935 | seed | Beta-453270 |
| 225 | -12.2 to 13.6 | WP-2015 | 11,470 +/- 40 | 13,405 to 13,255 | seed | Beta-412234 |
| 229.5 | -16 to -18 | WP-2016-3 | 11,285 +/- 30 | 13,206 to 13,069 | seed | UCI-190031 |
| 253 | -38 to -40 | WP-2016-3 | 12,330 +/- 40 | 14,628 to 14,099 | seed | Beta-470902 |
| 261 | -46 to -48 | WP-2016-3 | Modern | – | seed | UCI-190032 |
| 263 | -48.6 to 47.6 | WP-2015 | 12,920 +/- 50 | 15,615 to 15,265 | plant macrofossil | Beta-412235 |

[a]Core depth for all samples referenced to depths for WP-2016-3 based on lithology.

[b]Due to differential depths for core lithologies between cores, the relative depth for AMS samples is based on using the peat to mud transition (top of Unit II) common to all cores as a datum with samples indexed as above (+) or below (-) this transition.

[c]Only dates from 2016 cores were used to construct the Bayesian age/depth model.

[d]2-Sigma calibrated age ranges derived from INTCAL 13.

[e]Materials dated include aquatic seeds, plant fragments, and charcoal recovered from core samples and bulk dating of peat using the Alkali Insoluble fraction.

[f]Beta Analytic, Inc and UCIAMS (Keck Carbon Cycle AMS Lab, University of California, Irvine).

Supplementary Table 2. Bayesian age/depth model for White Pond.

| Depth (cm) | IntCal13_t | IntCal13_dt | Unmodeled age[1] (cal yr BP) | err |
|---|---|---|---|---|
| 162.0 | 9567 | 56 | 9540 | 17 |
| 162.5 | 9576 | 64 | | |
| 163.0 | 9586 | 73 | | |
| 163.5 | 9595 | 79 | | |
| 164.0 | 9605 | 86 | 10068 | 89 |
| 164.5 | 9611 | 87 | | |
| 165.0 | 9616 | 87 | | |
| 165.5 | 9622 | 88 | | |
| 166.0 | 9628 | 89 | | |
| 166.5 | 9634 | 89 | | |
| 167.0 | 9639 | 90 | | |
| 167.5 | 9645 | 90 | | |
| 168.0 | 9651 | 90 | | |
| 168.5 | 9657 | 90 | | |
| 169.0 | 9662 | 89 | | |
| 169.5 | 9668 | 89 | | |
| 170.0 | 9674 | 89 | 9667 | 66 |
| 170.5 | 9690 | 104 | | |
| 171.0 | 9706 | 118 | | |
| 171.5 | 9723 | 131 | | |
| 172.0 | 9739 | 142 | | |
| 172.5 | 9756 | 150 | | |
| 173.0 | 9772 | 158 | | |
| 173.5 | 9789 | 166 | | |
| 174.0 | 9805 | 169 | | |
| 174.5 | 9821 | 173 | | |
| 175.0 | 9838 | 177 | | |
| 175.5 | 9854 | 180 | | |
| 176.0 | 9870 | 181 | | |
| 176.5 | 9887 | 183 | | |
| 177.0 | 9903 | 184 | 10192 | 43 |
| 177.5 | 9909 | 183 | | |
| 178.0 | 9915 | 181 | | |
| 178.5 | 9921 | 180 | | |
| 179.0 | 9928 | 178 | 10605 | 41 |
| 179.5 | 9932 | 177 | | |
| 180.0 | 9936 | 175 | | |
| 180.5 | 9940 | 174 | | |
| 181.0 | 9944 | 173 | | |
| 181.5 | 9949 | 172 | | |
| 182.0 | 9953 | 170 | | |
| 182.5 | 9957 | 169 | | |
| 183.0 | 9961 | 167 | | |
| 183.5 | 9965 | 166 | | |

[1]Unmodeled ages accepted for inclusion in the Bayesian age/depth model.

Supplementary Table 2. (cont.)

| Depth (cm) | IntCal13_t | IntCal13_dt | Unmodeled age[1] (cal yr BP) | err |
|---|---|---|---|---|
| 184.5 | 9973 | 163 | | |
| 185.0 | 9977 | 162 | | |
| 185.5 | 9981 | 160 | | |
| 186.0 | 9986 | 159 | | |
| 186.5 | 9990 | 157 | | |
| 187.0 | 9994 | 156 | | |
| 187.5 | 9998 | 154 | | |
| 188.0 | 10002 | 153 | | |
| 188.5 | 10006 | 151 | | |
| 189.0 | 10011 | 150 | | |
| 189.5 | 10015 | 148 | 9852 | 100 |
| 190.0 | 10029 | 145 | | |
| 190.5 | 10043 | 142 | | |
| 191.0 | 10057 | 139 | | |
| 191.5 | 10071 | 135 | | |
| 192.0 | 10085 | 129 | | |
| 192.5 | 10099 | 124 | | |
| 193.0 | 10113 | 116 | | |
| 193.5 | 10128 | 109 | 10085 | 93 |
| 194.0 | 10141 | 108 | | |
| 194.5 | 10154 | 107 | | |
| 195.0 | 10167 | 105 | | |
| 195.5 | 10180 | 103 | | |
| 196.0 | 10193 | 100 | | |
| 196.5 | 10206 | 95 | | |
| 197.0 | 10219 | 90 | | |
| 197.5 | 10233 | 84 | | |
| 198.0 | 10246 | 77 | 10323 | 53 |
| 198.5 | 10250 | 74 | | |
| 199.0 | 10254 | 72 | | |
| 199.5 | 10258 | 69 | | |
| 200.0 | 10262 | 66 | | |
| 200.5 | 10268 | 65 | 10214 | 15 |
| 201.0 | 10274 | 66 | | |
| 201.5 | 10281 | 66 | | |
| 202.0 | 10287 | 67 | | |
| 202.5 | 10294 | 67 | | |
| 203.0 | 10300 | 67 | | |
| 203.5 | 10306 | 66 | | |
| 204.0 | 10313 | 65 | | |
| 204.5 | 10319 | 64 | | |
| 205.0 | 10326 | 62 | | |
| 205.5 | 10332 | 60 | | |
| 206.0 | 10339 | 58 | | |
| 206.5 | 10345 | 56 | 10364 | 62 |
| 207.0 | 10348 | 56 | | |

[1]Unmodeled ages accepted for inclusion in the Bayesian age/depth model.

Supplementary Table 2. (cont.)

| Depth (cm) | IntCal13_t | IntCal13_dt | Unmodeled age[1] (cal yr BP) | err |
|---|---|---|---|---|
| 208.0 | 10356 | 57 | | |
| 208.5 | 10359 | 57 | | |
| 209.0 | 10363 | 57 | | |
| 209.5 | 10367 | 57 | | |
| 210.0 | 10370 | 57 | | |
| 210.5 | 10383 | 60 | 10390 | 66 |
| 211.0 | 10976 | 892 | | |
| 211.5 | 11563 | 1056 | | |
| 212.0 | 12138 | 897 | | |
| 212.5 | 12733 | 55 | 12757 | 28 |
| 213.0 | 12738 | 55 | | |
| 213.5 | 12742 | 54 | | |
| 214.0 | 12745 | 54 | | |
| 214.5 | 12749 | 54 | | |
| 215.0 | 12752 | 54 | | |
| 215.5 | 12756 | 54 | | |
| 216.0 | 12759 | 54 | | |
| 216.5 | 12763 | 53 | | |
| 217.0 | 12766 | 53 | | |
| 217.5 | 12770 | 53 | | |
| 218.0 | 12773 | 53 | | |
| 218.5 | 12777 | 52 | | |
| 219.0 | 12779 | 52 | 12873 | 69 |
| 219.5 | 12781 | 54 | | |
| 220.0 | 12782 | 55 | | |
| 220.5 | 12784 | 56 | | |
| 221.0 | 12785 | 58 | 12625 | 36 |
| 221.5 | 12795 | 71 | | |
| 222.0 | 12806 | 82 | | |
| 222.5 | 12816 | 91 | | |
| 223.0 | 12826 | 100 | 13044 | 43 |
| 223.5 | 12838 | 103 | 12775 | 41 |
| 224.0 | 12855 | 114 | | |
| 224.5 | 12875 | 123 | | |
| 225.0 | 12895 | 132 | | |
| 225.5 | 12915 | 137 | | |
| 226.0 | 12935 | 142 | | |
| 226.5 | 12954 | 147 | | |
| 227.0 | 12975 | 146 | | |
| 227.5 | 12995 | 144 | | |
| 228.0 | 13015 | 143 | | |
| 228.5 | 13034 | 138 | | |
| 229.0 | 13054 | 133 | | |
| 229.5 | 13074 | 128 | 13132 | 36 |
| 230.0 | 13101 | 165 | | |
| 207.0 | 10348 | 56 | | |

[1]Unmodeled ages accepted for inclusion in the Bayesian age/depth model.
Area in brown represents YDB zone.

Supplementary Table 2. (cont.)

| Depth (cm) | IntCal13_t | IntCal13_dt | Unmodeled age[1] (cal yr BP) | err |
|---|---|---|---|---|
| 231.0 | 13154 | 229 | | |
| 231.5 | 13180 | 251 | | |
| 232.0 | 13206 | 273 | | |
| 232.5 | 13231 | 295 | | |
| 233.0 | 13257 | 309 | | |
| 233.5 | 13282 | 323 | | |
| 234.0 | 13308 | 338 | | |
| 234.5 | 13333 | 350 | | |
| 235.0 | 13359 | 362 | | |
| 235.5 | 13385 | 374 | | |
| 236.0 | 13410 | 380 | | |
| 236.5 | 13436 | 387 | | |
| 237.0 | 13461 | 394 | | |
| 237.5 | 13487 | 400 | | |
| 238.0 | 13513 | 406 | | |
| 238.5 | 13538 | 411 | | |
| 239.0 | 13564 | 414 | | |
| 239.5 | 13590 | 417 | | |
| 240.0 | 13616 | 421 | | |
| 240.5 | 13641 | 423 | | |
| 241.0 | 13667 | 426 | | |
| 241.5 | 13692 | 426 | | |
| 242.0 | 13717 | 424 | | |
| 242.5 | 13743 | 422 | | |
| 243.0 | 13769 | 419 | | |
| 243.5 | 13795 | 417 | | |
| 244.0 | 13820 | 415 | | |
| 244.5 | 13846 | 410 | | |
| 245.0 | 13871 | 405 | | |
| 245.5 | 13897 | 400 | | |
| 246.0 | 13922 | 393 | | |
| 246.5 | 13948 | 387 | | |
| 247.0 | 13974 | 382 | | |
| 247.5 | 14000 | 371 | | |
| 248.0 | 14025 | 359 | | |
| 248.5 | 14051 | 348 | | |
| 249.0 | 14077 | 334 | | |
| 249.5 | 14103 | 321 | | |
| 250.0 | 14129 | 308 | | |
| 250.5 | 14154 | 289 | | |
| 251.0 | 14180 | 269 | | |
| 251.5 | 14205 | 250 | | |
| 252.0 | 14231 | 224 | | |
| 252.5 | 14257 | 196 | | |
| 253.0 | 14283 | 167 | 14319 | 142 |

[1]Unmodeled ages accepted for inclusion in the Bayesian age/depth model.

Supplementary Table 2. (cont.)

| Depth (cm) | IntCal13_t | IntCal13_dt[1] |
|---|---|---|
| 253.5 | 14308 | 167 |
| 254.0 | 14334 | 167 |
| 254.5 | 14360 | 167 |
| 255.0 | 14385 | 167 |
| 255.5 | 14411 | 167 |
| 256.0 | 14437 | 167 |
| 256.5 | 14463 | 167 |
| 257.0 | 14488 | 167 |
| 257.5 | 14514 | 167 |
| 258.0 | 14540 | 167 |
| 258.5 | 14566 | 167 |
| 259.0 | 14591 | 167 |
| 259.5 | 14617 | 167 |
| 260.0 | 14643 | 167 |
| 260.5 | 14669 | 167 |
| 261.0 | 14694 | 167 |
| 261.5 | 14720 | 167 |
| 262.0 | 14746 | 167 |
| 262.5 | 14772 | 167 |
| 263.0 | 14798 | 167 |

[1]Ages interpolated below 253.5 cm.

Supplementary Table 3. Au, Pt, Pd, and Pt/Pd concentration data (ppb) for White Pond core 2016-2.

| Analyte Symbol | | Au | Pt | Pd | Pt/Pd |
|---|---|---|---|---|---|
| Units | | | ppb | | |
| Detection Limit | | | 0.1 | | |
| Analysis Method | [2]Depth | | FA-MS | | |
| [1]/Depth (cm) | Midpoint (cm) | | | | |
| +44 to +46 | 165 | 20 | 2.2 | 5 | 0.4 |
| +40 to +42 | 169 | 16 | 1.3 | 2.7 | 0.5 |
| +36 to +38 | 173 | 12 | 1.6 | 3.4 | 0.5 |
| +32 to +34 | 177 | 12 | 1.6 | 5.9 | 0.3 |
| +28 to +30 | 181.5 | 12 | 1.3 | 2.8 | 0.5 |
| +24 to +26 | 185.5 | 15 | 1.3 | 3.1 | 0.4 |
| +20 to +22 | 190 | 12 | 1.5 | 3 | 0.5 |
| +16 to +18 | 194 | 13 | 2.1 | 3.2 | 0.7 |
| +12 to +14 | 198 | 1120 | 2.4 | 5 | 0.5 |
| +10 to +12 | 200 | 400 | 2.2 | 4 | 0.6 |
| +8 to +10 | 202 | 84 | 2.7 | 4.5 | 0.6 |
| +6 to +8 | 204 | 263 | 2.4 | 5 | 0.5 |
| +4 to +6 | 206 | 68 | 3.8 | 8.6 | 0.4 |
| +2 to +4 | 208 | 230 | 2.7 | 6.1 | 0.4 |
| 0 to +2 | 210.4 | 60 | 0.9 | 1.6 | 0.6 |
| -2 to 0 | 212.5 | 31 | 0.9 | 1.1 | 0.8 |
| -4 to -2 | 215 | 23 | 1.1 | 1 | 1.1 |
| -6 to -4 | 217 | 17 | 0.7 | 0.9 | 0.8 |
| -8 to -6 | 219 | 38 | 1 | 2.3 | 0.4 |
| -10 to -8 | 221 | 13 | **4.2** | 0.9 | **4.7** |
| -12 to -10 | 223 | 12 | 0.6 | 0.9 | 0.7 |
| -14 to -12 | 225 | 12 | 1.7 | 1.4 | 1.2 |
| -16 to -14 | 227 | 14 | 0.7 | 1.1 | 0.6 |
| -18 to -16 | 229 | 61 | 0.6 | 0.8 | 0.8 |
| -20 to -18 | 231.5 | 29 | 0.6 | 0.7 | 0.9 |
| -22 to -20 | 233.5 | 27 | 0.5 | 0.7 | 0.7 |
| -24 to -22 | 235.5 | 59 | 0.5 | 0.7 | 0.7 |
| -26 to -24 | 238 | 44 | 0.5 | 0.7 | 0.7 |
| -28 to -26 | 240 | 26 | 0.6 | 0.7 | 0.9 |
| -30 to -28 | 242 | 75 | 0.7 | 0.8 | 0.9 |
| -32 to -30 | 244 | 76 | 0.6 | 0.7 | 0.9 |
| -34 to -32 | 246 | 70 | 0.7 | 0.8 | 0.9 |
| -36 to -34 | 248 | 62 | 1.1 | 1.1 | 1.0 |

[1]Depths 2-cm contiguous samples above and below the peat to mud transition in core 2016-2.

[2]Approximate midpoint for each 2-cm continuous sample referenced to depths for core 2016-3.

**Supplementary Table 4.** Dung fungi spore types in this study.

| TYPE | HABITAT |
|---|---|
| *Cercophora* | Herbivore dung, decaying wood[17,36] |
| *Coniochaeta* | Herbivore dung, decaying wood [36] |
| *Podospora* | Herbivore dung |
| *Sordaria* | Herbivore and omnivore dung, decaying wood [17,37] |
| *Sporormiella* | Herbivore dung[38] |

**Supplementary Table 5.** Generalized dung fungal spore patterns from Core WP-2016-3.

| Spore Zones-Dates | General Spore Characteristics | Interpretation and Relation to pollen record |
|---|---|---|
| **III**<br>**>13,300** | Concentration high | Abundance of megaherbivores (MHs)<br>Little vegetation change immediately preceding OR following decline of MHs |
| **II**<br>**13,300-12,700** | Concentration moderate-high | Possible decline of MHs, but not disappearance<br>Decline not likely result of changing water levels<br>Further decline coincides with onset of YD |
| **II & I**<br>**<12,700** | Concentration low | Probable disappearance of MHs<br>Noteable *Sporormiella* peak after ~8700 cal yr BP |

Table 6. Results from Enriched Libraries.

| Meta Information | | | Sequence Stats | |
|---|---|---|---|---|
| **Extract ID** | **Whitepond ID** | **Extraction Method** | **Total Reads** | **Mapped to (# of unique reads)** |
| **JK653** | Whitepond 1 | Qiagen PowerSoil | 342302 | Pig (1) |
| **JK654** | Whitepond 2 | Qiagen PowerSoil | 304553 | Human (1) |
| **JK655** | Whitepond 3 | Qiagen PowerSoil | 475862 | |
| **JK656** | Whitepond 4 | Qiagen PowerSoil | 345371 | |
| **JK657** | Whitepond 5 | Qiagen PowerSoil | 299887 | Human (1) |
| **JK658** | Whitepond 6 | Qiagen PowerSoil | 156778 | Human (1) |
| **JK659** | Whitepond 7 | Qiagen PowerSoil | 522341 | Pig (1) |
| **JK660** | Whitepond 8 | Qiagen PowerSoil | 382973 | |
| **JK661** | Whitepond 9 | Qiagen PowerSoil | 231213 | |
| **JK662** | Whitepond 10 | Qiagen PowerSoil | 331304 | Pig (1) |
| **JK663** | Whitepond 11 | Qiagen PowerSoil | 214289 | |
| **JK664** | Whitepond 12 | Qiagen PowerSoil | 275895 | Pig (1) |
| **JK665** | Whitepond 13 | Qiagen PowerSoil | 332894 | Pig (1) |
| **JK666** | Whitepond 14 | Qiagen PowerSoil | 428578 | |
| **JK667** | Whitepond 15 | Qiagen PowerSoil | 285723 | |
| **JK668** | Whitepond 16 | Qiagen PowerSoil | 239296 | |
| **JK669** | Whitepond 17 | Qiagen PowerSoil | 306907 | Human (1) |
| **JK670** | Whitepond 18 | Qiagen PowerSoil | 80776 | |
| **JK671** | Whitepond 19 | Qiagen PowerSoil | 293590 | Human (1), Pig (2) |
| **JK672** | Whitepond 20 | Qiagen PowerSoil | 406338 | |
| **JK673** | Whitepond 21 | Qiagen PowerSoil | 298364 | |
| **JK674** | Whitepond 22 | Qiagen PowerSoil | 288501 | Pig (1) |
| **JK675** | Whitepond 23 | Qiagen PowerSoil | 376949 | |
| **JK676** | Whitepond 24 | Qiagen PowerSoil | 384278 | |
| **JK677** | Whitepond 25 | Qiagen PowerSoil | 309254 | |
| **JK-EC89** | Extraction Control | Qiagen PowerSoil | 137051 | |

Table 7. Results from Shotgun Libraries.

| Shotgun Libraries | | | | |
|---|---|---|---|---|
| Meta Information | | | Sequence Stats | |
| **Extract ID** | **Whitepond ID** | **Extraction Method** | **Total Reads** | **Mapped to (# of unique reads)** |
| **JK653** | Whitepond 1 | Qiagen Powersoil | 9575082 | Human (151) |
| **JK655** | Whitepond 3 | Qiagen Powersoil | 12664827 | Human (30) |
| **JK661** | Whitepond 9 | Qiagen Powersoil | 13409930 | Human (30) |
| **JK664** | Whitepond 12 | Qiagen Powersoil | 10264998 | Human (18) |
| **JK666** | Whitepond 14 | Qiagen Powersoil | 12601380 | Human (32) |
| **JK670** | Whitepond 18 | Qiagen Powersoil | 10911045 | Human (41) |
| **JK672** | Whitepond 20 | Qiagen Powersoil | 9646484 | Human (32) |
| **JK673** | Whitepond 21 | Qiagen Powersoil | 11614696 | Human (32) |
| **JK677** | Whitepond 25 | Qiagen Powersoil | 11281343 | Human (45) |
| **JK718** | Whitepond 1 | Seersholm | 33682488 | Human (1232) |
| **JK719** | Whitepond 3 | Seersholm | 33477150 | Human (211) |
| **JK720** | Whitepond 9 | Seersholm | 33196215 | Human (150) |
| **JK721** | Whitepond 12 | Seersholm | 35363957 | Human (113) |
| **JK722** | Whitepond 14 | Seersholm | 36267491 | Human (126) |
| **JK723** | Whitepond 18 | Seersholm | 36448939 | Human (216) |
| **JK724** | Whitepond 20 | Seersholm | 29969513 | Human (200) |
| **JK725** | Whitepond 21 | Seersholm | 34388946 | Human (120) |
| **JK-EC92** | Extraction Control | Seersholm | 10525596 | Human (789) |

Supplementary Table 8. Carbon and soot data for core 2016-1.

| Midpoint (cm) | OC (%) | $d^{13}C_{OC}$ (‰) | TN (%) | $\delta^{15}N_{TN}$ (‰) | OC/TN (mol:mol) | Soot Carbon (%) | Soot Carbon/OC (%) | $\delta^{13}C_{Soot}$ ‰ |
|---|---|---|---|---|---|---|---|---|
| 1.5 | 38.07 | -25.34 | 3.20 | 1.19 | 14.28 | 1.38 | 3.63 | -23.38 |
| 1.5 | 37.41 | -25.88 | 3.08 | 1.52 | 14.56 | 1.24 | 3.31 | -23.10 |
| 1.5 | NQ | -18.19 | 3.38 | 1.20 | 0.00 | NQ | NQ | -22.42 |
| 7.5 | NQ | -20.94 | 5.38 | 0.93 | 0.00 | NQ | NQ | -18.02 |
| 16.5 | NQ | -1.38 | 3.45 | 0.18 | 0.00 | 1.81 | NQ | -20.75 |
| 28.5 | 48.90 | -25.68 | 2.91 | 0.64 | 20.17 | 3.10 | 6.33 | -19.79 |
| 35.5 | 52.84 | -25.85 | 3.60 | 1.60 | 17.64 | 1.75 | 3.30 | -20.52 |
| 45.5 | 54.55 | -25.65 | 2.99 | 0.53 | 21.86 | NQ | NQ | NQ |
| 52.5 | 53.91 | -26.07 | 2.88 | -0.19 | 22.47 | 1.78 | 3.31 | -18.95 |
| 61.5 | NQ | -23.51 | 2.85 | -0.87 | 0.00 | 2.11 | NQ | -19.45 |
| 68.5 | 47.83 | -25.52 | 2.24 | 0.09 | 25.67 | 0.70 | 1.45 | -18.10 |
| 75.5 | 46.80 | -26.06 | 2.49 | -0.22 | 22.53 | 0.58 | 1.24 | -20.02 |
| 84.5 | 55.31 | -25.48 | 3.18 | 0.03 | 20.87 | 1.42 | 2.58 | -15.91 |
| 91.5 | 57.59 | -27.63 | 3.16 | -0.70 | 21.84 | NQ | NQ | NQ |
| 97.5 | 49.99 | -26.37 | 2.75 | -0.26 | 21.82 | NQ | NQ | NQ |
| 103.5 | 53.32 | -27.11 | 2.94 | -0.27 | 21.79 | 1.59 | 2.98 | -19.33 |
| 112.5 | 48.93 | -27.65 | 2.45 | -0.71 | 24.00 | 3.32 | 6.78 | -19.79 |
| 120.25 | 49.85 | -25.87 | 2.68 | 0.50 | 22.29 | 1.30 | 2.62 | -20.06 |
| 129.5 | 31.86 | -26.06 | 2.03 | 1.13 | 18.86 | 0.68 | 2.13 | -19.41 |
| 129.5 | 22.54 | -26.23 | 1.46 | 0.50 | 18.51 | 0.59 | 2.61 | -17.09 |
| 129.5 | 24.29 | -26.01 | 1.50 | 0.41 | 19.44 | 0.75 | 3.08 | -18.21 |
| 142.5 | 52.18 | -26.88 | 2.36 | -0.36 | 26.49 | 1.15 | 2.21 | -21.30 |
| 151.5 | 53.18 | -27.14 | 2.66 | -0.99 | 24.00 | NQ | NQ | NQ |
| 161.5 | 45.72 | -27.08 | 2.26 | 0.20 | 24.32 | 0.66 | 1.43 | -19.38 |
| 172.5 | 51.96 | -26.12 | 2.75 | 0.70 | 22.66 | 1.21 | 2.34 | -22.19 |
| 182.5 | 46.21 | -26.66 | 2.28 | 0.61 | 24.36 | 1.13 | 2.44 | -20.40 |
| 185.5 | 49.94 | -26.93 | 2.54 | 0.17 | 23.58 | 1.88 | 3.76 | -21.53 |
| 185.5 | NQ | -27.92 | 3.70 | 0.71 | 0.00 | NQ | NQ | -20.32 |
| 191.5 | 48.63 | -26.81 | 2.54 | 0.24 | 22.96 | 0.72 | 1.49 | -20.32 |
| 197.5 | 43.50 | -27.05 | 2.31 | 0.16 | 22.61 | 1.71 | 3.94 | -20.73 |
| 227.5 | 13.35 | -26.65 | 0.80 | 2.46 | 19.97 | 0.86 | 6.43 | -21.52 |
| 239.5 | 24.99 | -26.57 | 1.40 | 2.00 | 21.49 | 0.91 | 3.66 | -19.95 |
| 245.5 | 7.77 | NQ | 0.47 | 3.14 | 19.65 | 0.82 | 10.59 | -22.43 |
| 251.5 | 8.79 | -26.65 | 0.59 | 4.51 | 17.94 | 0.78 | 8.90 | -20.49 |
| 251.5 | 8.11 | -24.48 | 0.53 | 2.73 | 18.36 | 0.65 | 7.97 | -21.48 |
| 251.5 | 8.24 | -22.94 | 0.55 | 2.88 | 17.98 | 0.87 | 10.62 | -20.39 |
| 254.5 | NQ | -24.89 | 0.60 | 3.14 | 0.00 | NQ | NQ | NQ |
| 254.5 | 6.95 | -25.12 | 0.48 | 3.94 | 17.47 | 1.24 | 17.91 | -21.84 |
| 260.5 | 8.27 | -24.79 | 0.56 | 3.41 | 17.71 | 0.65 | 7.90 | -20.56 |

Supplementary Table 9. Bulk C and N isotope data for White Pond core (2016-3) sediments.

**Sample Data**

| Sample ID | Depth (cm)[a] | Tray Position | Sample Mass (mg) | Carbon Content (mg) | Carbon Content (%) | Corrected δ13C | Nitrogen Content (mg) | Nitrogen Content (%) | Corrected δ15N | C/N Ratio |
|---|---|---|---|---|---|---|---|---|---|---|
| 1 | 179 | A9 | 0.611 | 0.300239705 | 49.139068 | -27.88994991 | 0.016420818 | 2.687531513 | -0.450937792 | 18.28408997 |
| 2 | 181 | A10 | 0.63 | 0.26938676 | 42.75980325 | -28.31169093 | 0.0148875 | 2.363095274 | 0.140699996 | 18.09482831 |
| 3 | 183 | A11 | 0.643 | 0.273815993 | 42.58413584 | -27.77338101 | 0.015368734 | 2.390160735 | 0.165453118 | 17.81643185 |
| 4 | 185 | A12 | 0.635 | 0.293813732 | 46.2698791 | -27.89672309 | 0.016183884 | 2.548643119 | 0.201974898 | 18.15471094 |
| 5 | 187 | B1 | 0.623 | 0.291808718 | 46.83928063 | -27.94460254 | 0.016586548 | 2.66236732 | -0.067190736 | 17.5930948 |
| 6 | 189 | B2 | 0.62 | 0.270716082 | 43.66388419 | -27.96681529 | 0.01535523 | 2.476649924 | 0.047249951 | 17.63022047 |
| 7 | 191 | B5 | 0.631 | 0.305137786 | 48.35781082 | -28.01028697 | 0.017357504 | 2.750792993 | 0.166384293 | 17.57958921 |
| 8 | 193 | B6 | 0.628 | 0.283068843 | 45.07465657 | -27.91321904 | 0.016608646 | 2.644688829 | 0.101505808 | 17.04346314 |
| 9 | 195 | B7 | 0.635 | 0.295129264 | 46.47704947 | -27.85763099 | 0.017164765 | 2.703112589 | 0.126177916 | 17.19390071 |
| 10 | 197 | B8 | 0.587 | 0.279315303 | 47.58352687 | -27.74215211 | 0.015298758 | 2.606262057 | 0.210995452 | 18.25738388 |
| 11 | 199 | B9 | 0.518 | 0.237899492 | 45.92654285 | -27.88536319 | 0.013902322 | 2.683846016 | 0.141737887 | 17.11221231 |
| 12 | 201 | B10 | 0.51 | 0.239929327 | 47.04496613 | -27.81313846 | 0.013896798 | 2.724862353 | 0.263007613 | 17.26507986 |
| 13 | 203 | C1 | 0.508 | 0.237924313 | 46.83549474 | -27.77114131 | 0.013806567 | 2.717828102 | 0.245924429 | 17.23269206 |
| 14 | 205 | C2 | 0.511 | 0.201599638 | 39.45198403 | -27.43240174 | 0.011949154 | 2.338386212 | 0.514723358 | 16.87145769 |
| 15 | 207 | C3 | 0.51 | 0.179108732 | 35.11935927 | -27.76774917 | 0.010781672 | 2.114053282 | 0.41895347 | 16.61233403 |
| 16 | 209 | C4 | 0.508 | 0.228853487 | 45.04989898 | -27.60807147 | 0.012733613 | 2.506616717 | 0.460308209 | 17.97239229 |
| 17 | 211 | C5 | 0.523 | 0.260508989 | 49.81051414 | -27.79867171 | 0.015445461 | 2.953242977 | 0.350274854 | 16.8663786 |
| 18 | 213 | C6 | 0.57 | 0.100830451 | 17.68955273 | -26.64929548 | 0.006221004 | 1.091404233 | 1.011374397 | 16.20806681 |
| 19 | 215 | C9 | 0.573 | 0.095405605 | 16.65019292 | -26.18248908 | 0.006123407 | 1.068657429 | 1.709219803 | 15.58047739 |
| 20 | 217 | C10 | 0.562 | 0.120767515 | 21.48888166 | -26.94284547 | 0.007853146 | 1.397356965 | 2.67677114 | 15.37823348 |
| 21 | 219 | C11 | 0.579 | 0.111818037 | 19.31226896 | -26.32403906 | 0.007513091 | 1.297597752 | 2.272767815 | 14.88309373 |
| 22 | 221 | C12 | 0.572 | 0.111500876 | 19.49316006 | -26.35129611 | 0.007527823 | 1.316052904 | 2.390035817 | 14.81183621 |
| 23 | 225 | D1 | 0.584 | 0.09454789 | 16.18970714 | -27.09298575 | 0.006621827 | 1.133874533 | 2.818889203 | 14.27821745 |
| 24 | 227 | D2 | 0.59 | 0.075948421 | 12.87261365 | -25.84476901 | 0.005576495 | 0.945168686 | 2.938548418 | 13.61938228 |
| 25 | 229 | D5 | 0.621 | 0.114617335 | 18.45689769 | -26.4874594 | 0.008258266 | 1.329833496 | 3.085720845 | 13.87910421 |
| 26 | 231 | D6 | 2.314 | 0.304045647 | 13.139397 | -26.4417398 | 0.019610216 | 0.847459628 | 3.114315353 | 15.50445186 |
| 27 | 233 | D7 | 2.422 | 0.406643341 | 16.78956817 | -26.57147225 | 0.025623791 | 1.057959974 | 2.968130259 | 15.86975744 |
| 28 | 235 | D8 | 2.568 | 0.196047929 | 7.634265129 | -24.8851163 | 0.013154078 | 0.512230461 | 4.009071587 | 14.90396552 |
| 29 | 237 | D9 | 2.651 | 0.208665451 | 7.871197696 | -25.05891729 | 0.014758598 | 0.556718161 | 3.908831353 | 14.13856822 |
| 30 | 239 | D10 | 2.67 | 0.257715207 | 9.652254962 | -25.64394675 | 0.01798237 | 0.673497022 | 3.639720301 | 14.33154809 |
| 32 | 243 | A10 | 2.603 | 0.223750655 | 8.595876119 | -24.52574355 | 0.014946869 | 0.574217015 | 3.491462758 | 14.96973425 |
| 33 | 245 | A11 | 2.485 | 0.199796048 | 8.040082399 | -24.59376736 | 0.014104687 | 0.567593057 | 3.44949786 | 14.1652233 |
| 34 | 247 | A12 | 2.261 | 0.197078994 | 8.716452614 | -24.06139606 | 0.01367469 | 0.60480715 | 3.531180603 | 14.41195366 |
| 35 | 249 | B1 | 2.316 | 0.203451669 | 8.784614378 | -24.16943205 | 0.014467114 | 0.624659508 | 3.903868535 | 14.06304437 |
| 36 | 251 | B2 | 2.465 | 0.200207552 | 8.122010208 | -24.45840088 | 0.013973231 | 0.566865355 | 3.783224697 | 14.32793544 |
| 37 | 253 | B5 | 2.56 | 0.370959175 | 14.49059278 | -24.24822862 | 0.016743031 | 0.654024657 | 3.620948085 | 22.15603438 |
| 38 | 255 | B6 | 2.627 | 0.207124202 | 7.884438581 | -23.9579624 | 0.014652013 | 0.557746984 | 4.257985179 | 14.13622809 |
| 39 | 257 | B7 | 2.725 | 0.297066017 | 10.90150521 | -25.68363845 | 0.020198985 | 0.741247159 | 3.267059291 | 14.70697739 |
| 40 | 259 | B8 | 2.843 | 0.258593209 | 9.095786449 | -25.62456847 | 0.017682269 | 0.621958118 | 3.547668759 | 14.62443561 |
| 41 | 261 | B9 | 2.905 | 0.293968463 | 10.1193963 | -25.56745771 | 0.019863587 | 0.683772351 | 3.580277054 | 14.79936457 |
| 42 | 263 | B10 | 3.002 | 0.300442605 | 10.00808144 | -25.37136313 | 0.020380198 | 0.678887357 | 3.27727863 | 14.74188809 |

[a]midpoint of 2-cm interval continuous samples.

Supplementary Table 10. Ultratrace 4 elemental geochemistry of core 2016-3.

| Report Number: A18-05509 | | | | | | | | | | | | | | | | | | | |
|---|---|---|---|---|---|---|---|---|---|---|---|---|---|---|---|---|---|---|---|
| Report Date: 18/6/2018 | | | | | | | | | | | | | | | | | | | |
| Analyte Symbol | B | Li | Na | Mg | Al | K | Ca | Cd | V | Cr | Mn | Fe | Hf | Ni | Er | Be | Ho | Hg | Ag |
| Unit Symbol | ppm | ppm | % | % | % | % | % | ppm | ppm | ppm | ppm | % | ppm | ppm | ppm | ppm | ppm | ppb | ppm |
| Detection Limit | 20 | 0.5 | 0.01 | 0.01 | 0.01 | 0.01 | 0.01 | 0.1 | 1 | 1 | 1 | 0.01 | 0.1 | 0.5 | 0.1 | 0.1 | 0.1 | 10 | 0.05 |
| Analysis Method | TD-MS | TD-MS | TD-MS | TD-MS | TD-MS | TD-MS | TD-MS | TD-MS | TD-MS | TD-MS | TD-MS | TD-MS | TD-MS | TD-MS | TD-MS | TD-MS | TD-MS | TD-MS | TD-MS |
| /Depth midpoint (cm) | | | | | | | | | | | | | | | | | | | |
| 204.3 | < 20 | 7.9 | 0.14 | 0.08 | 3.12 | 0.33 | 0.27 | 0.8 | 32 | 54 | 91 | 0.6 | 1.3 | 27.5 | 0.9 | 0.8 | 0.4 | 150 | 0.08 |
| 206.3 | < 20 | 5.7 | 0.13 | 0.07 | 2.7 | 0.26 | 0.26 | 0.6 | 29 | 32 | 70 | 0.42 | 0.8 | 11.8 | 0.8 | 0.9 | 0.3 | 90 | 0.07 |
| 208.4 | < 20 | 6.3 | 0.07 | 0.06 | 2.7 | 0.26 | 0.2 | 0.4 | 28 | 35 | 91 | 0.4 | 1.1 | 20.1 | 0.8 | 0.9 | 0.3 | 60 | < 0.05 |
| 210.5 | < 20 | 4.9 | 0.08 | 0.06 | 2.31 | 0.22 | 0.22 | 0.4 | 30 | 33 | 91 | 0.38 | 0.7 | 13.1 | 0.8 | 0.9 | 0.3 | 90 | 0.07 |
| 212.6 | < 20 | 12.3 | 0.09 | 0.09 | 4.21 | 0.38 | 0.11 | 0.6 | 48 | 35 | 85 | 0.51 | 1.8 | 12.1 | 1 | 1.2 | 0.4 | 70 | < 0.05 |
| 214.8 | < 20 | 18.9 | 0.12 | 0.14 | 7.8 | 0.56 | 0.1 | 0.2 | 83 | 55 | 101 | 0.78 | 2.5 | 16.7 | 1.4 | 1.9 | 0.5 | 80 | < 0.05 |
| 216.9 | < 20 | 20.9 | 0.12 | 0.13 | 8.97 | 0.63 | 0.06 | < 0.1 | 80 | 54 | 83 | 0.79 | 2.7 | 18.1 | 1.5 | 2.1 | 0.5 | 40 | < 0.05 |
| 218.9 | < 20 | 22.7 | 0.13 | 0.14 | 9.47 | 0.69 | 0.04 | 0.1 | 78 | 62 | 86 | 0.79 | 3 | 19.5 | 1.6 | 1.9 | 0.6 | 30 | < 0.05 |
| 221.1 | < 20 | 22.6 | 0.18 | 0.16 | 8.98 | 0.71 | 0.12 | < 0.1 | 95 | 63 | 99 | 0.97 | 3.2 | 18.4 | 1.6 | 2.2 | 0.6 | 40 | < 0.05 |
| 223.2 | < 20 | 22.9 | 0.12 | 0.15 | 8.97 | 0.7 | 0.11 | 0.3 | 81 | 58 | 92 | 0.81 | 3.1 | 17.9 | 1.6 | 2 | 0.6 | 60 | < 0.05 |
| 225.2 | 20 | 23.4 | 0.13 | 0.12 | 6.31 | 0.72 | 0.1 | 0.2 | 87 | 64 | 81 | 0.79 | 3.2 | 17.7 | 1.3 | 1.9 | 0.5 | 70 | < 0.05 |
| 227.3 | 20 | 24.1 | 0.12 | 0.14 | 9.21 | 0.79 | 0.1 | 0.2 | 87 | 50 | 84 | 0.85 | 3.4 | 17.6 | 1.6 | 2.2 | 0.6 | 90 | < 0.05 |
| 229.4 | < 20 | 22.9 | 0.13 | 0.15 | 8.56 | 0.77 | 0.11 | 0.2 | 97 | 61 | 115 | 0.87 | 3.3 | 17.9 | 1.7 | 2.4 | 0.7 | 80 | < 0.05 |
| 231.4 | < 20 | 23.9 | 0.12 | 0.16 | 9.51 | 0.79 | 0.12 | 0.2 | 103 | 60 | 92 | 0.85 | 3.3 | 19.1 | 1.8 | 2.4 | 0.7 | 50 | < 0.05 |
| 233.5 | < 20 | 29.9 | 0.16 | 0.19 | > 10.0 | 0.95 | 0.12 | 0.2 | 61 | 65 | 98 | 1 | 2.5 | 20.2 | 1.6 | 2.5 | 0.7 | 70 | < 0.05 |
| 235.7 | < 20 | 29.5 | 0.14 | 0.18 | > 10.0 | 0.94 | 0.09 | 0.2 | 61 | 69 | 86 | 0.92 | 2.6 | 23.5 | 1.7 | 2.5 | 0.7 | 50 | < 0.05 |
| 237.8 | < 20 | 29.1 | 0.16 | 0.18 | > 10.0 | 0.92 | 0.12 | 0.3 | 73 | 61 | 83 | 0.96 | 2.8 | 21.2 | 1.7 | 2.5 | 0.7 | 60 | < 0.05 |

Supplemental Table 10. *(cont.)*

| Report Number: A18-05509 | | | | | | | | | | | | | | | | | | | |
|---|---|---|---|---|---|---|---|---|---|---|---|---|---|---|---|---|---|---|---|
| Report Date: 18/6/2018 | | | | | | | | | | | | | | | | | | | |
| Analyte Symbol | Cs | Co | Eu | Bi | Se | Ga | As | Rb | Y | Zr | Nb | Mo | In | Sn | Sb | Te | Ba | La | Ce |
| Unit Symbol | ppm | ppm | ppm | ppm | ppm | ppm | ppm | ppm | ppm | ppm | ppm | ppm | ppm | ppm | ppm | ppm | ppm | ppm | ppm |
| Detection Limit | 0.05 | 0.1 | 0.05 | 0.02 | 0.1 | 0.1 | 0.1 | 0.2 | 0.1 | 1 | 0.1 | 0.05 | 0.1 | 1 | 0.1 | 0.1 | 1 | 0.1 | 0.1 |
| Analysis Method | TD-MS | TD-MS | TD-MS | TD-MS | TD-MS | TD-MS | TD-MS | TD-MS | TD-MS | TD-MS | TD-MS | TD-MS | TD-MS | TD-MS | TD-MS | TD-MS | TD-MS | TD-MS | TD-MS |
| /Depth midpoint (cm) | | | | | | | | | | | | | | | | | | | |
| 204.3 | 1.32 | 4.2 | 0.6 | 0.14 | 3.7 | 8.2 | 4.6 | 19.4 | 11.8 | 52 | 6.7 | 2.2 | < 0.1 | 2 | 0.2 | < 0.1 | 131 | 31.6 | 58.1 |
| 206.3 | 1.02 | 3.8 | 0.5 | 0.09 | 3.8 | 6.1 | 2.8 | 15.7 | 10.4 | 35 | 5.1 | 20.6 | < 0.1 | 1 | 0.2 | < 0.1 | 114 | 17.9 | 34.4 |
| 208.4 | 1.06 | 3.1 | 0.5 | 0.09 | 3.5 | 6.3 | 2 | 16.2 | 11.5 | 42 | 5.6 | 1.23 | < 0.1 | 1 | 0.2 | < 0.1 | 121 | 19.1 | 37 |
| 210.5 | 0.86 | 3.2 | 0.4 | 0.07 | 3.6 | 4.9 | 7.3 | 12.7 | 10.8 | 29 | 4.3 | 1.01 | < 0.1 | 1 | 0.1 | < 0.1 | 114 | 17.5 | 35 |
| 212.6 | 1.58 | 2.9 | 0.7 | 0.14 | 4 | 10.5 | 1.7 | 24.6 | 12.7 | 66 | 6.2 | 1.4 | < 0.1 | 2 | 0.3 | < 0.1 | 156 | 33.6 | 65.4 |
| 214.8 | 2.15 | 4 | 1.1 | 0.22 | 3.9 | 18.2 | 2 | 37.4 | 16.2 | 93 | 8.7 | 2.51 | < 0.1 | 3 | 0.3 | < 0.1 | 197 | 45 | 88.9 |
| 216.9 | 2.39 | 4.3 | 1.2 | 0.24 | 3.4 | 20 | 1.6 | 41.6 | 16.5 | 96 | 1.9 | 0.93 | < 0.1 | 3 | 0.2 | < 0.1 | 218 | 49.8 | 94.9 |
| 218.9 | 2.54 | 4 | 1.2 | 0.26 | 3.5 | 21.1 | < 0.1 | 44.6 | 17.8 | 107 | 1.7 | 1.62 | < 0.1 | 3 | 0.2 | < 0.1 | 221 | 49.8 | 94.1 |
| 221.1 | 2.57 | 4.2 | 1.3 | 0.26 | 3.7 | 21.2 | 0.8 | 44.4 | 18.7 | 113 | 10.3 | 1.67 | < 0.1 | 3 | 0.3 | < 0.1 | 234 | 49.7 | 98.1 |
| 223.2 | 2.51 | 4.1 | 1.3 | 0.25 | 3.7 | 21 | 4.4 | 44.4 | 18.8 | 109 | 5.8 | 1.04 | < 0.1 | 3 | 0.1 | < 0.1 | 244 | 52.1 | 100 |
| 225.2 | 2.47 | 4 | 1 | 0.24 | 3.5 | 20 | 0.7 | 42.1 | 15.5 | 115 | 16.7 | 2.54 | < 0.1 | 3 | 0.3 | < 0.1 | 239 | 42.4 | 83.8 |
| 227.3 | 2.59 | 4.1 | 1.3 | 0.28 | 3.4 | 22.5 | 0.8 | 48.4 | 19.2 | 122 | 4.2 | 1.05 | < 0.1 | 3 | 0.1 | < 0.1 | 254 | 51.7 | 97.4 |
| 229.4 | 2.56 | 4.2 | 1.4 | 0.27 | 3.6 | 21.6 | 0.2 | 47.1 | 20.7 | 119 | 8.9 | 1.91 | < 0.1 | 3 | 0.2 | < 0.1 | 250 | 52.5 | 100 |
| 231.4 | 2.77 | 4 | 1.4 | 0.29 | 3.8 | 22.9 | 0.7 | 49.4 | 20.9 | 115 | 8.6 | 1.87 | < 0.1 | 3 | 0.2 | < 0.1 | 258 | 53.1 | 101 |
| 233.5 | 3.34 | 4.1 | 1.5 | 0.32 | 3.1 | 26.4 | < 0.1 | 59.2 | 20.4 | 81 | 0.5 | 0.15 | < 0.1 | 2 | < 0.1 | < 0.1 | 311 | 54.3 | 103 |
| 235.7 | 3.24 | 4.3 | 1.5 | 0.32 | 3.2 | 26.6 | < 0.1 | 57.9 | 20.7 | 82 | 0.9 | 0.21 | < 0.1 | 3 | < 0.1 | < 0.1 | 291 | 55.7 | 105 |
| 237.8 | 3.27 | 4.4 | 1.5 | 0.32 | 3 | 26.2 | < 0.1 | 57.2 | 21 | 89 | 1.2 | 0.16 | < 0.1 | 3 | < 0.1 | < 0.1 | 304 | 54.4 | 104 |

Supplemental Table 10. *(cont.)*

Report Number: A18-05509

Report Date: 18/6/2018

| Analyte Symbol | Cs | Co | Eu | Bi | Se | Ga | As | Rb | Y | Zr | Nb | Mo | In | Sn | Sb | Te | Ba | La | Ce |
|---|---|---|---|---|---|---|---|---|---|---|---|---|---|---|---|---|---|---|---|
| Unit Symbol | ppm | ppm | ppm | ppm | ppm | ppm | ppm | ppm | ppm | ppm | ppm | ppm | ppm | ppm | ppm | ppm | ppm | ppm | ppm |
| Detection Limit | 0.05 | 0.1 | 0.05 | 0.02 | 0.1 | 0.1 | 0.1 | 0.2 | 0.1 | 1 | 0.1 | 0.05 | 0.1 | 1 | 0.1 | 0.1 | 1 | 0.1 | 0.1 |
| Analysis Method | TD-MS | TD-MS | TD-MS | TD-MS | TD-MS | TD-MS | TD-MS | TD-MS | TD-MS | TD-MS | TD-MS | TD-MS | TD-MS | TD-MS | TD-MS | TD-MS | TD-MS | TD-MS | TD-MS |
| /Depth midpoint (cm) | | | | | | | | | | | | | | | | | | | |
| 204.3 | 1.32 | 4.2 | 0.6 | 0.14 | 3.7 | 8.2 | 4.6 | 19.4 | 11.8 | 52 | 6.7 | 2.2 | < 0.1 | 2 | 0.2 | < 0.1 | 131 | 31.6 | 58.1 |
| 206.3 | 1.02 | 3.8 | 0.5 | 0.09 | 3.8 | 6.1 | 2.8 | 15.7 | 10.4 | 35 | 5.1 | 20.6 | < 0.1 | 1 | 0.2 | < 0.1 | 114 | 17.9 | 34.4 |
| 208.4 | 1.06 | 3.1 | 0.5 | 0.09 | 3.5 | 6.3 | 2 | 16.2 | 11.5 | 42 | 5.6 | 1.23 | < 0.1 | 1 | 0.2 | < 0.1 | 121 | 19.1 | 37 |
| 210.5 | 0.86 | 3.2 | 0.4 | 0.07 | 3.6 | 4.9 | 7.3 | 12.7 | 10.8 | 29 | 4.3 | 1.01 | < 0.1 | 1 | 0.1 | < 0.1 | 114 | 17.5 | 35 |
| 212.6 | 1.58 | 2.9 | 0.7 | 0.14 | 4 | 10.5 | 1.7 | 24.6 | 12.7 | 66 | 6.2 | 1.4 | < 0.1 | 2 | 0.3 | < 0.1 | 156 | 33.6 | 65.4 |
| 214.8 | 2.15 | 4 | 1.1 | 0.22 | 3.9 | 18.2 | 2 | 37.4 | 16.2 | 93 | 8.7 | 2.51 | < 0.1 | 3 | 0.3 | < 0.1 | 197 | 45 | 88.9 |
| 216.9 | 2.39 | 4.3 | 1.2 | 0.24 | 3.4 | 20 | 1.6 | 41.6 | 16.5 | 96 | 1.9 | 0.93 | < 0.1 | 3 | 0.2 | < 0.1 | 218 | 49.8 | 94.9 |
| 218.9 | 2.54 | 4 | 1.2 | 0.26 | 3.5 | 21.1 | < 0.1 | 44.6 | 17.8 | 107 | 1.7 | 1.62 | < 0.1 | 3 | 0.2 | < 0.1 | 221 | 49.8 | 94.1 |
| 221.1 | 2.57 | 4.2 | 1.3 | 0.26 | 3.7 | 21.2 | 0.8 | 44.4 | 18.7 | 113 | 10.3 | 1.67 | < 0.1 | 3 | 0.3 | < 0.1 | 234 | 49.7 | 98.1 |
| 223.2 | 2.51 | 4.1 | 1.3 | 0.25 | 3.7 | 21 | 4.4 | 44.4 | 18.8 | 109 | 5.8 | 1.04 | < 0.1 | 3 | 0.1 | < 0.1 | 244 | 52.1 | 100 |
| 225.2 | 2.47 | 4 | 1 | 0.24 | 3.5 | 20 | 0.7 | 42.1 | 15.5 | 115 | 16.7 | 2.54 | < 0.1 | 3 | 0.3 | < 0.1 | 239 | 42.4 | 83.8 |
| 227.3 | 2.59 | 4.1 | 1.3 | 0.28 | 3.4 | 22.5 | 0.8 | 48.4 | 19.2 | 122 | 4.2 | 1.05 | < 0.1 | 3 | 0.1 | < 0.1 | 254 | 51.7 | 97.4 |
| 229.4 | 2.56 | 4.2 | 1.4 | 0.27 | 3.6 | 21.6 | 0.2 | 47.1 | 20.7 | 119 | 8.9 | 1.91 | < 0.1 | 3 | 0.2 | < 0.1 | 250 | 52.5 | 100 |
| 231.4 | 2.77 | 4 | 1.4 | 0.29 | 3.8 | 22.9 | 0.7 | 49.4 | 20.9 | 115 | 8.6 | 1.87 | < 0.1 | 3 | 0.2 | < 0.1 | 258 | 53.1 | 101 |
| 233.5 | 3.34 | 4.1 | 1.5 | 0.32 | 3.1 | 26.4 | < 0.1 | 59.2 | 20.4 | 81 | 0.5 | 0.15 | < 0.1 | 2 | < 0.1 | < 0.1 | 311 | 54.3 | 103 |
| 235.7 | 3.24 | 4.3 | 1.5 | 0.32 | 3.2 | 26.6 | < 0.1 | 57.9 | 20.7 | 82 | 0.9 | 0.21 | < 0.1 | 3 | < 0.1 | < 0.1 | 291 | 55.7 | 105 |
| 237.8 | 3.27 | 4.4 | 1.5 | 0.32 | 3 | 26.2 | < 0.1 | 57.2 | 21 | 89 | 1.2 | 0.16 | < 0.1 | 3 | < 0.1 | < 0.1 | 304 | 54.4 | 104 |

Supplementary Table 11. Error Analysis.

| Standard | Hg contents (mg/kg) | Number of analyses | 2-sigma standard deviation (± mg/kg) | Accepted value (mg/kg) |
|---|---|---|---|---|
| TORT-3 SRM | 0.298 | 4 | 0.011 | 0.292 |
| DORM-4 SRM | 0.405 | 4 | 0.025 | 0.412 |

Supplementary Table 12. Mercury, LOI, and grain size data.

| Sample ID | Depth (midpoint) | Hg [mg/kg or ppm] | LOI% | %Mineral | Sed Lab IDs | sed n | D10 (um) | D50 (um) | D90 (um) |
|---|---|---|---|---|---|---|---|---|---|
| WP-16-3-1 | 167 | 0.1679 | 79.037 | 20.963 | 4420, 4421, 4422 | 3 | 6.668 | 28.001 | 69.628 |
| WP-16-3-2 | 169 | 0.1780 | 81.577 | 18.423 | 4423, 4424, 4425 | 3 | 5.302 | 28.733 | 79.023 |
| WP-16-3-3 | 171 | 0.1629 | 73.265 | 26.735 | 4426, 4427, 4428 | 3 | 4.787 | 24.772 | 69.465 |
| WP-16-3-4 | 173 | 0.1598 | 77.211 | 22.789 | 4429 | 1 | 5.406 | 28.733 | 77.427 |
| WP-16-3-5 | 175 | 0.1789 | 78.664 | 21.336 | 4430 | 1 | 4.346 | 22.765 | 62.409 |
| WP-16-3-6 | 177 | 0.1507 | 77.923 | 22.077 | 4431 | 1 | 4.826 | 22.909 | 63.959 |
| WP-16-3-7 | 179 | 0.1605 | 83.911 | 16.089 | 4432 | 1 | 4.620 | 23.199 | 67.554 |
| WP-16-3-8 | 181 | 0.1383 | 73.008 | 26.992 | 4433 | 1 | 5.419 | 26.995 | 74.756 |
| WP-16-3-9 | 183 | 0.1419 | 77.196 | 22.804 | 4434 | 1 | 8.493 | 39.163 | |
| WP-16-3-10 | 185 | 0.1245 | 82.008 | 17.992 | 4435 | 1 | 4.492 | 22.195 | 61.350 |
| WP-16-3-11 | 187 | 0.1933 | 79.617 | 20.383 | 4436 | 1 | 4.226 | 21.707 | 62.079 |
| WP-16-3-12 | 189 | 0.1523 | 81.536 | 18.464 | 4437 | 1 | 4.126 | 20.993 | 61.081 |
| WP-16-3-13 | 191 | 0.1624 | 81.555 | 18.445 | 4438, 4439, 4440 | 3 | 4.286 | 23.094 | 68.649 |
| WP-16-3-14 | 193 | 0.1483 | 82.085 | 17.915 | 4441 | 1 | 5.361 | 27.003 | 73.717 |
| WP-16-3-15 | 195 | 0.1969 | 78.559 | 21.441 | 4442 | 1 | 4.679 | 23.974 | 71.194 |
| WP-16-3-16 | 197 | 0.1730 | 80.681 | 19.319 | 4443 | 1 | 4.568 | 23.653 | 66.970 |
| WP-16-3-17 | 199 | 0.2315 | 78.009 | 21.991 | 4444 | 1 | 4.442 | 22.993 | 66.949 |
| WP-16-3-18 | 201 | 0.1679 | 77.859 | 22.141 | 4445 | 1 | 4.691 | 24.201 | 72.919 |
| WP-16-3-19 | 203 | 0.1736 | 78.875 | 21.125 | 4446 | 1 | 3.801 | 20.823 | 65.489 |
| WP-16-3-20 | 205 | 0.1847 | 70.506 | 29.494 | 4447, 4448, 4449 | 3 | 4.048 | 21.427 | 64.807 |
| WP-16-3-21 | 207 | 0.2251 | 79.379 | 20.621 | 4450, 4451, 4452 | 3 | 4.633 | 22.975 | |
| WP-16-3-22 | 209 | 0.1588 | 70.847 | 29.153 | 4453 | 1 | 4.179 | 20.484 | 60.501 |
| WP-16-3-23 | 211 | 0.2110 | 68.363 | 31.637 | 4454 | 1 | 4.240 | 22.533 | 66.665 |
| WP-16-3-24 | 213 | 0.1773 | 42.663 | 57.337 | 4455 | 1 | 2.663 | 17.462 | 53.843 |
| WP-16-3-25 | 215 | 0.0762 | 39.793 | 60.207 | 4456 | 1 | 3.728 | 21.761 | 63.691 |
| WP-16-3-26 | 217 | 0.1427 | 36.551 | 63.449 | 4457 | 1 | 3.222 | 19.851 | 60.530 |
| WP-16-3-27 | 219 | 0.1464 | 30.771 | 69.229 | 4458 | 1 | 2.519 | 17.789 | 60.367 |
| WP-16-3-28 | 221 | 0.1327 | 31.577 | 68.423 | 4459 | 1 | 2.339 | 16.345 | 52.847 |
| WP-16-3-29 | 225 | 0.1000 | 30.764 | 69.236 | 4460, 4461, 4462 | 3 | 2.408 | 16.776 | 55.687 |
| WP-16-3-30 | 227 | 0.0755 | 29.336 | 70.664 | 4463 | 1 | 2.463 | 17.197 | 61.016 |
| WP-16-3-31 | 229 | 0.0783 | 25.449 | 74.551 | 4464, 4465, 4466 | 3 | 2.786 | 18.620 | 65.036 |
| WP-16-3-32 | 231 | 0.1922 | 28.541 | 71.459 | 4467 | 1 | 2.638 | 19.137 | 69.966 |
| WP-16-3-33 | 233 | 0.0813 | 18.496 | 81.504 | 4468 | 1 | 2.131 | 14.620 | 57.966 |
| WP-16-3-34 | 235 | 0.0746 | 18.054 | 81.946 | 4469 | 1 | 1.873 | 13.132 | 48.893 |
| WP-16-3-35 | 237 | 0.0751 | 18.832 | 81.168 | 4470, 4471, 4472 | 3 | 1.998 | 14.140 | |
| WP-16-3-36 | 239 | 0.0893 | 18.943 | 81.057 | 4473 | 1 | 2.470 | 17.189 | 66.239 |
| WP-16-3-37 | 241 | 0.1587 | 19.461 | 80.539 | 4474 | 1 | 2.114 | 14.446 | 53.504 |
| WP-16-3-38 | 243 | 0.1296 | 22.755 | 77.245 | 4475, 4476, 4477 | 3 | 2.185 | 15.903 | 59.960 |
| WP-16-3-39 | 245 | 0.1184 | 19.590 | 80.410 | 4478 | 1 | 2.099 | 14.846 | 55.526 |
| WP-16-3-40 | 247 | 0.0776 | 19.997 | 80.003 | 4479 | 1 | 2.241 | 15.749 | 59.125 |
| WP-16-3-41 | 249 | 0.1951 | 19.721 | 80.279 | 4480 | 1 | 2.466 | 18.757 | 71.870 |
| WP-16-3-42 | 251 | 0.0815 | 21.453 | 78.547 | 4481 | 1 | 2.052 | 14.322 | 52.902 |
| WP-16-3-43 | 253 | 0.2091 | 22.070 | 77.930 | 4482 | 1 | 1.872 | 13.523 | 52.671 |
| WP-16-3-44 | 255 | 0.0926 | 20.140 | 79.860 | 4483 | 1 | 1.901 | 13.567 | 51.690 |
| WP-16-3-45 | 257 | 0.0785 | 20.690 | 79.310 | 4484, 4485, 4486 | 3 | 2.019 | 15.108 | 59.305 |
| WP-16-3-46 | 259 | 0.0787 | 23.305 | 76.695 | 4487 | 1 | 1.976 | 13.344 | 49.001 |
| WP-16-3-47 | 261 | 0.1073 | 20.508 | 79.492 | 4488 | 1 | 2.041 | 15.009 | 60.131 |
| WP-16-3-48 | 263 | 0.0563 | 18.122 | 81.878 | 4489 | 1 | 2.041 | 15.533 | 63.026 |
